# TMPRSS2-induced Golgi disruption restricts the incorporation of virus envelope glycoproteins into virions

**DOI:** 10.1038/s44319-026-00797-2

**Published:** 2026-05-19

**Authors:** Sayuri Seki, Shigeyoshi Harada, Akiko Sugimoto-Ishige, Midori Unno, Machie Sakuma, Shinsuke Ito, Hiroyuki Yamamoto, Aki Tanabe, Saori Matsuoka, Chieko Makino-Okamura, Sewon Ki, Hidehiro Fukuyama, Michishige Harada, Kazuya Tsumagari, Koshi Imami, Takashi Saito, Masato Kubo, Tadaki Suzuki, Haruhiko Koseki, Tetsuro Matano, Kosuke Miyauchi

**Affiliations:** 1 AIDS Research Center, National Institute of Infectious Diseases, Japan Institute for Health Security, Tokyo, Japan; 2 Laboratory for Infectious Diseases and Immunology, Center for Integrative Medical Sciences, RIKEN, Yokohama, Japan; 3 Laboratory for Developmental Genetics, Center for Integrative Medical Sciences, RIKEN, Yokohama, Japan; 4 Division of Immunology, Near InfraRed Photo-Immuno Therapy Research Institute, Kansai Medical University, Hirakata, Japan; 5 Laboratory for Cytokine Regulation, Center for Integrative Medical Sciences, RIKEN, Yokohama, Japan; 6 Infectious Diseases Research Unit, Center for Integrative Medical Sciences, RIKEN, Yokohama, Japan; 7 Cell Integrative Science Laboratory, Graduate School of Medical Life Science, Yokohama City University, Kanagawa, Japan; 8 INSERM EST, Strasbourg, France; 9 Drug Discovery Antibody Platform Unit, Center for Integrative Medical Sciences, RIKEN, Yokohama, Japan; 10 Drug Discovery Structural Biology Platform Unit, Center for Integrative Medical Sciences, RIKEN, Yokohama, Japan; 11 Proteome Homeostasis Research Unit, Center for Integrative Medical Sciences, RIKEN, Yokohama, Japan; 12 Division of Molecular Pathology, Research Institute for Biomedical Science, Tokyo University of Science, Noda, Japan; 13 Department of Infectious Disease Pathology, National Institute of Infectious Diseases, Japan Institute for Health Security, Tokyo, Japan; 14 Department of Infectious Disease Pathobiology, Graduate School of Medicine, Chiba University, Chiba, Japan

**Keywords:** Membranes & Trafficking, Microbiology, Virology & Host Pathogen Interaction, Signal Transduction

## Abstract

Understanding the relationship between viral proteins and host factors is essential for developing strategies to control virus infections. Severe acute respiratory syndrome coronavirus 2 (SARS-CoV-2) infects host cells using angiotensin converting enzyme 2 (ACE2) and transmembrane protease serine 2 (TMPRSS2) as viral receptor and priming protease during the viral entry phase. Here, we report that TMPRSS2 reduces the infectivity of SARS-CoV-2 and HIV-1 during the viral production phase. Treatment of virus-producing cells with a TMPRSS2 inhibitor increases the production of infectious virions. TMPRSS2 enzymatic activity specifically disrupts the trans-Golgi by phosphorylating Golgi stacking proteins through ERK activation, which disturbs virion spike incorporation and structural maturation of the viral envelope glycoproteins, causing lower viral infectivity. We find that SARS-CoV-2 envelope protein (E protein) counteracts this TMPRSS2 activity to rescue SARS-CoV-2-S incorporation. These results demonstrate negative regulation of viral envelope glycoprotein incorporation by TMPRSS2 and reveal that SARS-CoV-2 regulates the Golgi system to create an optimal viral replication environment.

## Synopsis

The transmembrane protease TMPRSS2 inhibits the incorporation of SARS-CoV-2 spike into virions during the viral production phase by mediating Golgi disruption. The SARS-CoV-2 envelope protein counteracts this activity to promote SARS-CoV-2 spike incorporation.

- TMPRSS2 reduces the infectivity of SARS-CoV-2 and HIV-1 during the viral production phase.
- TMPRSS2 inhibits the incorporation of SARS-CoV-2 spike and HIV-1 envelope protein into virions.
- TMPRSS2 disrupts the trans-Golgi by phosphorylating Golgi stacking proteins through ERK activation.
- SARS-CoV-2 envelope protein counteracts this TMPRSS2 activity.

## Abstract (web summary)

The transmembrane protease TMPRSS2 inhibits the incorporation of SARS-CoV-2 spike into virions during the viral production phase by mediating Golgi disruption. The SARS-CoV-2 envelope protein counteracts this activity to promote SARS-CoV-2 spike incorporation.

## Graphical abstract

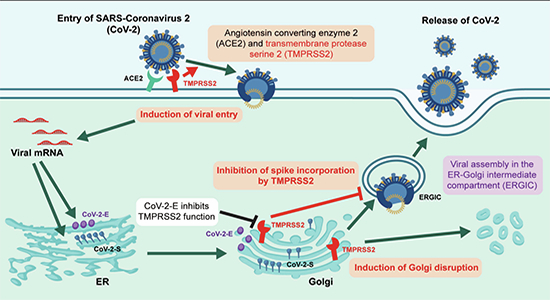

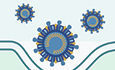

## Introduction

Viruses, including severe acute respiratory syndrome coronavirus 2 (SARS-CoV-2) and human immunodeficiency virus type 1 (HIV-1), rely on various host factors for replication. By contrast, host restriction factors inhibit viral replication using diverse processes (Baggen et al, 2021). For example, a host factor essential for a specific step in the viral replication process could restrict subsequent replication steps (Martin-Sancho et al, 2021), as in the case of CD4, a primary viral receptor for HIV-1 entry, which inhibits viral envelope protein (Env) expression but is downregulated by the viral protein u (Vpu) encoded by the HIV-1 genome to overcome this restriction (Willey et al, 1992).

SARS-CoV-2 enters host cells by means of virus–cell membrane fusion induced by a conformational change in the SARS-CoV-2 spike protein (CoV-2-S) (Jackson et al, 2022). The binding to angiotensin converting enzyme 2 (ACE2) and the cleavage of CoV-2-S by transmembrane protease serine 2 (TMPRSS2, TM2) or cathepsins elicits a CoV-2-S conformational change which induces membrane fusion (Jackson et al, 2022; Koch et al, 2021). TM2 is a membrane-bound protease that cleaves the CoV-2-S and hemagglutinin (HA) of the influenza virus and is closely related to the infectivity of these viruses (Hoffmann et al, 2020a; Sakai et al, 2014).

Many viral membrane proteins, including CoV-2-S and HIV-1 Env, are dependent on the ER-Golgi trafficking machinery in the host cell. These viral membrane proteins are transported to the Golgi via the ER and are assembled with other viral structural proteins in the ER-Golgi intermediate compartment (ERGIC) and cytoplasm (Bracquemond and Muriaux, 2021; Checkley et al, 2011). The precursor viral membrane proteins are relayed to furin and furin-like protease for cleavage to produce the mature protein (Checkley et al, 2011; Takeda, 2022). Cleavage and viral spike structural maturation are closely related and vital for the incorporation of viral membrane proteins into virions (Checkley et al, 2011; Nguyen et al, 2021; Tien et al, 2022).

Furin and furin-like proteases become active in the trans-Golgi during viral maturation, where they cleave viral membrane proteins (Anderson et al, 2002; Braun and Sauter, 2019). TM2 is thought to be activated by autodigestion during maturation in the Golgi apparatus (Fuentes-Prior, 2021; Zhang et al, 2022b); however, its physiological role in cells remains unclear. The effect of TM2 expression in virus-producing cells on enveloped virus infectivity is also unknown.

In the present study, we investigated the effects of TM2 expression on the infectivity of SARS-CoV-2 and HIV-1 in virus-producing cells. We found that TM2 inhibited the incorporation of viral envelope glycoproteins into virions, resulting in lower infectivity. We also found that the SARS-CoV-2 envelope protein (CoV-2-E) rescued the TM2-inhibited incorporation of CoV-2-S. These results demonstrate the effect of TM2 on membrane protein biosynthesis, revealing that SARS-CoV-2 regulates host membrane proteins to create an optimal environment for viral replication.

## Results

### TM2 expression in virus-producing cells reduces SARS-CoV-2 infectivity

**Figure EV1:**
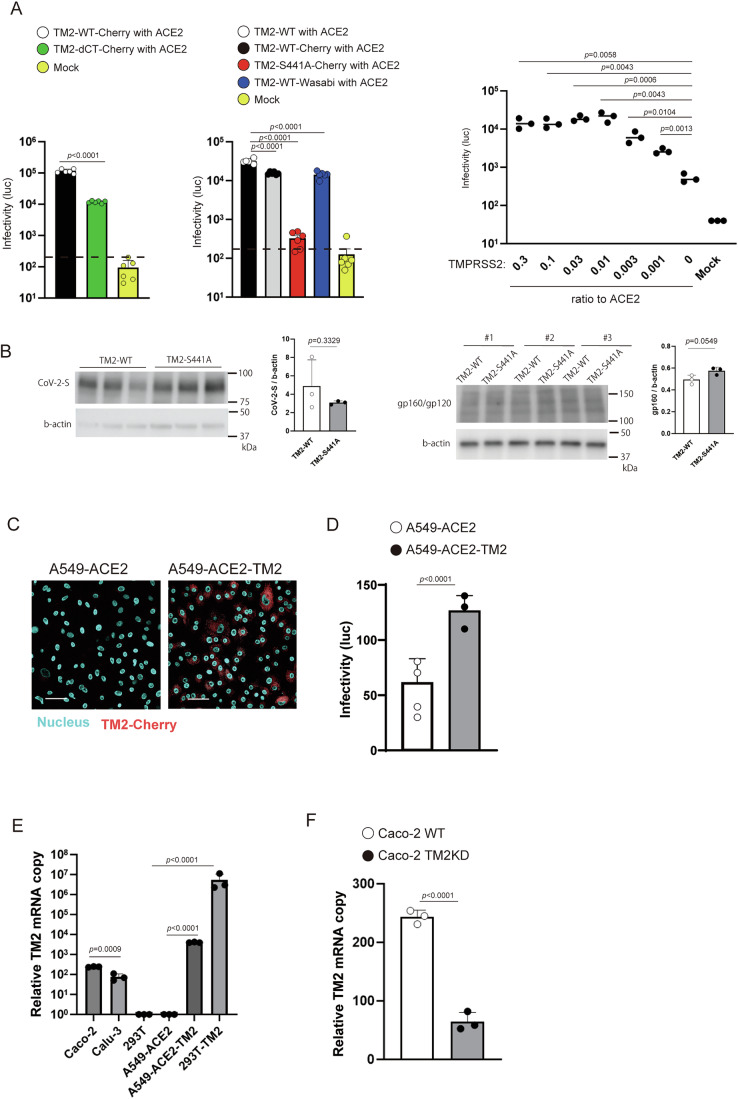
Fluorescent protein-labeled TM2 and TM2 expression in cell lines. (**A**) Infectivity of CoV-2-S pseudotyped lentiviruses was determined in ACE2- and TMPRSS2-WT (TM2-WT), TMPRSS2-WT-mCherry- (TM2-WT-Cherry)-, TMPRSS2-dCT-mCherry- (TM2-dCT-Cherry)-, TMPRSS2-S441A-mCherry- (TM2-S441A-Cherry)-, and/or TMPRSS2-WT-Wasabi- (TM2-WT-Wasabi)-expressing 293T cells (left and middle, the quantified results from a representative experiment in the two experiments independently conducted are expressed as mean ± SEM, *n* = 6). Infectivity of CoV-2-S pseudotyped lentiviruses was determined in ACE2- and TM2-WT-Cherry (with indicated ratio of plasmids) expressing 293 T cells (right, the quantified results are expressed as mean ± SEM of three separate experiments, *n* = 3). Representative data from three independent experiments are shown. (**B**) Immunoblotting analysis of cell lysate from 293 T cells expressing CoV-2-S (left) or HIV-1 Env (right) and TM2-WT-mCherry (TM2-WT) or TM2-S441A-mCherry (TM2-S441A). The ratio of CoV-2-S2 or gp160 to beta-actin was calculated based on band density (the quantified results are expressed as mean ± SEM of three separate experiments, *n* = 3). (**C**) Fluorescence microscopy analysis of A549-ACE2 and A549-ACE2-TMPRSS2-mCherry- (TM2)-expressing cells. (**D**) Infectivity of SARS-CoV-2 spike protein pseudotyped lentiviruses in ACE2- or ACE2- and TM2-expressing A549 cells (the quantified results from a representative experiment in the two experiments independently conducted are expressed as mean ± SEM, *n* = 4). (**E**) q-PCR analysis for TMPRSS2 (TM2) mRNA in indicated cell lines (A549-ACE2-TM2: A549-ACE2 cells stably expressing TM2; 293T-TM2: 293T cells transiently expressing TM2) (the quantified results from a representative experiment in the two experiments independently conducted are expressed as mean ± SEM, *n* = 3). (**F**) q-PCR analysis for TM2 mRNA in original Caco-2 (Caco-2 WT) and TM2 knockdown Caco-2 (Caco-2 TM2-KD) cells (the quantified results from a representative experiment in the two experiments independently conducted are expressed as mean ± SEM, *n* = 3). Scale bars indicate 50 μm. The results of Student’s *t* tests are shown above the bars.

**Figure 1:**
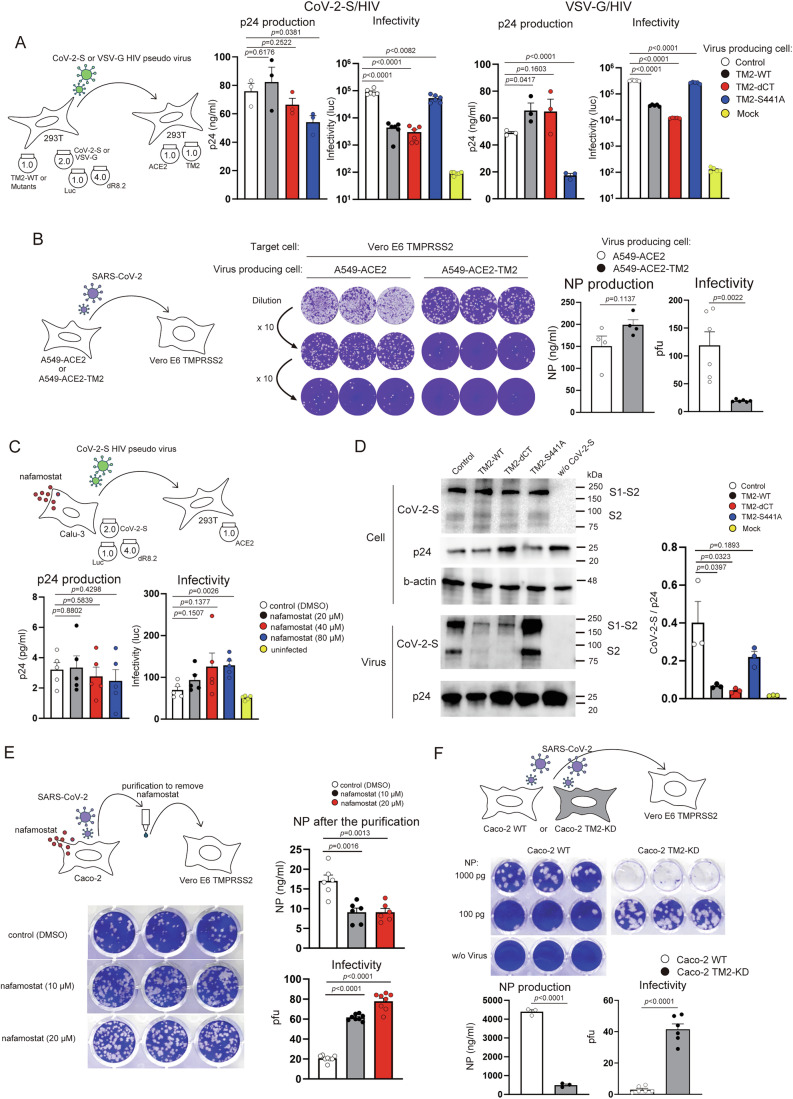
TM2 in virus-producing cells reduces SARS-CoV-2 infectivity. (**A**) Experimental scheme and luciferase activity for the infectivity of SARS-CoV-2-Spike (CoV-2-S) or vesicular stomatitis virus G protein (VSV-G) pseudotyped lentiviruses produced in CD4-mCherry- (Control)-, TMPRSS2-mCherry- (TM2-WT)-, TMPRSS2-dCT-mCherry- (TM2-dCT)-, or TMPRSS2-S441A-mCherry-(TM2-S441A)-expressing 293T cells. The infectivity of pseudotyped lentiviruses (3 ng/well) normalized with p24 amount (left in panels, the quantified results are expressed as mean ± SEM of three separate experiments, *n* = 3) was determined in ACE2- and TM2-expressing 293T cells (right in panels, the quantified results from a representative experiment in the three experiments independently conducted are expressed as mean ± SEM, *n* = 6 for CoV-2-S, *n* = 5 for VSV-G). (**B**) Experimental scheme (left) and plaque-formation assay using Vero E6 TMPRSS2 cells to test the infectivity of SARS-CoV-2 produced in A549-ACE2 or A549-ACE2 stably expressing TMPRSS2 (A549-ACE2-TM2) cells (middle, *n* = 3). A549-ACE2 or A549-ACE2-TM2 cells infected with SARS-CoV-2. After normalization with SARS-CoV-2 nucleocapsid protein (NP, left in panels, the quantified results are expressed as mean ± SEM of four separate experiments, *n* = 4) amounts, the viruses were used in a plaque-formation assay using Vero E6 TMPRSS2 cells. Plaque-forming unit (pfu) per 80 pg of NP is shown (right, the quantified results from a representative experiment in the three experiments independently conducted are expressed as mean ± SEM, *n* = 6). (**C**) Experimental scheme (top) and luciferase activity for the infectivity of CoV-2-S pseudotyped lentiviruses produced in Calu-3 cells. Calu-3 cells transfected with CoV-2-S and lentiviral plasmids were treated with the indicated concentrations of nafamostat. After normalization with p24 amounts (bottom left, the quantified results are expressed as mean ± SEM of five separate experiments, *n* = 5), the pseudotyped lentiviruses (1.0 pg/well) were used to infect 293T cells expressing ACE2 (293T-ACE2), but not TM2, and the infectivity was determined as luciferase activity (bottom right, the quantified results from a representative experiment in the two experiments independently conducted are expressed as mean ± SEM, *n* = 5). (**D**) Immunoblotting analysis of CoV-2-S pseudotyped lentiviruses in 293T cells expressing CD4-mCherry- (Control)-, TM2-WT, TM2-dCT, or TM2-S441A. The ratio of CoV-2-S2 to p24 was calculated based on band density (right, the quantified results are expressed as mean ± SEM of three separate experiments, *n* = 3). The samples were obtained from the same experiment, and the gels and blots were processed in parallel. (**E**) Experimental scheme (left top) and plaque-formation assay of SARS-CoV-2 produced in nafamostat-treated Caco-2 cells (left bottom, *n* = 3). Caco-2 cells infected with SARS-CoV-2 were treated with the indicated concentrations of nafamostat. After the 96 hr culture, viruses were purified and normalized with SARS-CoV-2 NP amounts (right top, the quantified results are expressed as mean ± SEM of six separate experiments, *n* = 6), the viruses were used in a plaque-formation assay using Vero E6 TMPRSS2 cells. Plaque-forming unit (pfu) per 25 pg of NP is shown (right bottom, the quantified results from a representative experiment in the three experiments independently conducted are expressed as mean ± SEM, *n* = 8). (**F**) Experimental scheme (top) and plaque-formation assay (middle) of SARS-CoV-2 produced in Caco-2 cells (*n* = 3). Original (WT) and TM2-knockdown (KD) Caco-2 cells were infected with SARS-CoV-2. After the 96 hr culture and normalization with SARS-CoV-2 NP amounts (bottom left, the quantified results are expressed as mean ± SEM of three separate experiments, *n* = 3), the viruses were used in a plaque-formation assay using Vero E6 TMPRSS2 cells. Plaque-forming unit (pfu) per 100 pg of NP is shown (right bottom, the quantified results from two experiments among the three experiments independently conducted are expressed as mean ± SEM, *n* = 6). The number in vectors indicates the ratio of plasmid used for transfection. The results of Student’s *t* tests are shown above the bars. Source data are available online for this figure.

To investigate the effect of TM2 expression on SARS-CoV-2 infectivity in virus-producing cells, we first examined the infectivity of pseudotyped viruses produced by 293 T cells expressing TM2. To examine the roles of the cytoplasmic tail and enzyme activity, TM2 variants lacking the cytoplasmic tail (dCT) or variants in which the active center, serine 441 (Fraser et al, 2022), was replaced by alanine (S441A) were generated. Mutants lacking the TM2 cytoplasmic tail supported SARS-CoV-2 entry, whereas those lacking the TM2 enzymatic activity did not (Fig. EV1A). TM2 enzyme activity did not affect SARS-CoV-2 spike expression in cells (Fig. EV1B), but TM2-WT or TM2-dCT expression in virus-producing cells severely reduced the infectivity of CoV-2-S or VSV-G pseudotyped lentiviruses (Fig. 1A). To determine how TM2 affects infectivity of authentic SARS-CoV-2 instead of CoV-2-S pseudotyped viruses in stable TM2 expressing cells, we examined infectivity of SARS-CoV-2, produced in A549-ACE2 cells with stable TM2 expression (A549-ACE2-TM2, Fig. EV1C–E), after the normalization with viral nucleoprotein (NP) amount and found that the infectivity of SARS-CoV-2 was significantly reduced by TM2 expression (*P* = 0.0022, Fig. 1B). This occurred despite an increase in the entry of CoV-2-S-pseudotyped viruses due to TM2 expression (Fig. EV1D), suggesting a major inhibitory effect due to TM2 expression. These results indicate that even low levels of TM2 result in a marked inhibitory effect on viral infectivity. To examine the effect of TM2 on SARS-CoV-2 infectivity under physiological conditions, we treated Calu-3 cells with nafamostat (Hoffmann et al, 2020b), a TM2 inhibitor, during the production of the CoV-2-S pseudotyped lentivirus. Nafamostat treatment of Calu-3 cells transfected with CoV-2-S and lentiviral expression plasmids increased the lentivirus infectivity in ACE2-expressing 293 T cells (Fig. 1C). These results indicate that physiological levels of TM2 expression suppress the production of infectious CoV-2-S pseudotyped lentivirus. Next, viral components were examined to determine the cause of reduced viral infectivity. Immunoblotting analysis of the virions of CoV-2-S pseudotyped lentiviruses revealed a reduction in the incorporation of CoV-2-S (Fig. 1D). We next examined the effects of physiological TM2 expression on authentic SARS-CoV-2 production. We treated SARS-CoV-2-infected Caco-2 cells with nafamostat for 3 days, then SARS-CoV-2 particles were purified to remove nafamostat. Infectivity of newly produced SARS-CoV-2 in Caco-2 cells per NP protein was significantly increased with the nafamostat treatment during virus production (Fig. 1E). Nafamostat exhibits inhibitory effects against TMPRSS family proteases other than TM2, and since the expression levels of TMPRSS family proteases also vary between cell lines, we performed TM2 knockdown in Caco-2 cells to investigate the impact of TM2 itself on infectious virus production (Fig. EV1F). The replication efficacy of SARS-CoV-2, as measured by NP production, was significantly reduced with the TM2 knockdown (Fig. 1F). On the other hand, the infectivity of viruses after normalization with NP amount was markedly increased (Fig. 1F). These results indicate that TM2 in Caco-2 has an inhibitory effect on infectious SARS-CoV-2 production.

### TM2 inhibits the production of infectious lentivirus particles

**Figure 2:**
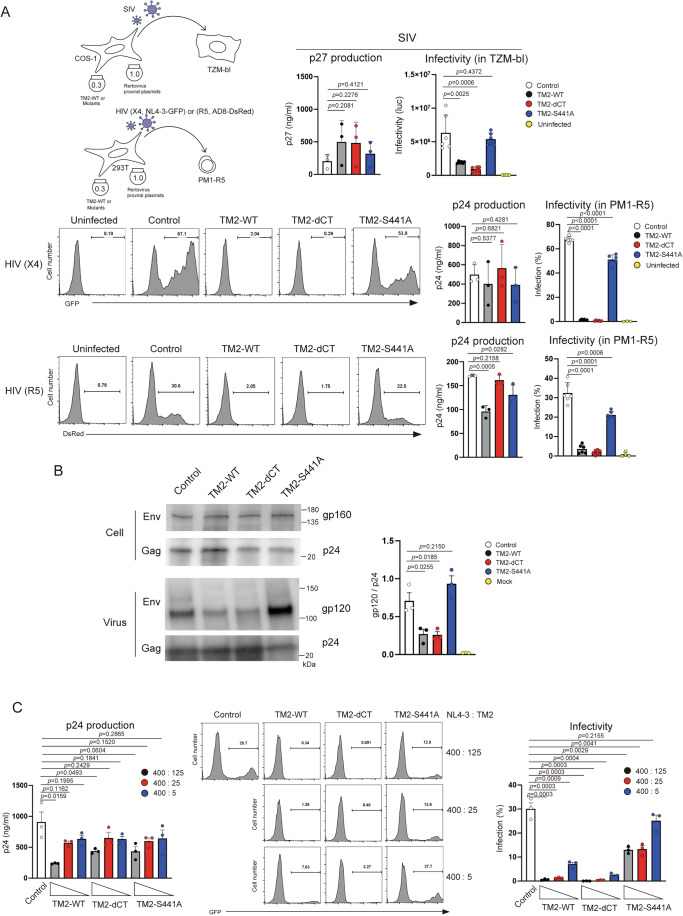
Expression of TM2 in virus-producing cells reduces lentivirus infectivity. (**A**) Experimental scheme (top left) and the infectivity of SIV (top right), fluorescence protein derived HIV-1 NL4-3, an X4 tropic or HIV-1 AD8, an R5 tropic (bottom) produced in mCherry (Control), TMPRSS2-Cherry (TM2-WT), TMPRSS2-dCT-Cherry (TM2-dCT), or TMPRSS2-S441A-Cherry (TM2-S441A)-expressing 293T cells. The infectivity of SIV normalized with p27 amount (left in panels, 3 ng/well, the quantified results are expressed as mean ± SEM of three separate experiments, *n* = 3) was determined in TZM-bl cells (right, the quantified results from a representative experiment in the two experiments independently conducted are expressed as mean ± SEM, *n* = 6). The infectivity of NL4-3 and AD8 normalized with p24 amount (left in panels, 3 ng/well, the quantified results are expressed as mean ± SEM of three separate experiments, *n* = 3) was determined in PM1-R5 cells (right, the quantified results from a representative experiment in the two experiments independently conducted are expressed as mean ± SEM, *n* = 5–6). (**B**) Viral protein profiles of virions and producing cells for HIV-1 NL4-3. The ratio of gp120 to p24 was calculated based on band density (right, the quantified results are expressed as mean ± SEM of three separate experiments, *n* = 3). (**C**) 293T cells were transfected with 400 ng of the NL4-3 proviral plasmid and 125, 25, or 5 ng of mCherry (control), TMPRSS2-Cherry (TM2-WT), TMPRSS2-dCT-Cherry (TM2-dCT), or TMPRSS2-S441A-Cherry (TM2-S441A). The supernatant from the 48-h culture was measured for p24 amount (left, the quantified results are expressed as mean ± SEM of three separate experiments, *n* = 3) and used for infection of PM1-R5 cells, and the % of infected cells was determined by expression of reporter fluorescence protein using the flow-cytometer (right, the quantified results are expressed as mean ± SEM of three separate experiments, *n* = 3). The number in vectors indicates the ratio of plasmid used for transfection. The results of Student’s *t* tests are shown above the bars. Source data are available online for this figure.

Since the effect of TM2 expression was observed in VSV-G as well as SARS-CoV-2 spike protein, we hypothesized that the effect of TM2 is common among enveloped viruses and investigated its impact on simian immunodeficiency virus (SIV) and HIV-1 production. TM2 expression in virus-producing cells severely reduced the infectivity of SIV, X4 tropic, and R5 tropic-HIV-1 (Fig. 2A). Immunoblotting analysis of the HIV-1 virions revealed a reduction in the incorporation of the HIV-1 envelope protein (Env) (Fig. 2B). To determine the effect of TM2 at various expression levels on the infectivity of viruses, we examined the infectivity of HIV-1 by reducing the amount of the TM2 expression plasmid and found a marked decrease in infectivity even after transfection with the TM2 expression plasmid that was 80 times lower than that of the HIV-1 provirus plasmid (Fig. 2C). These results suggest that TM2 expression has inhibitory effect on production of infectious lentivirus particles.

### TM2 causes CoV-2-S intracellular structural changes

**Figure EV2:**
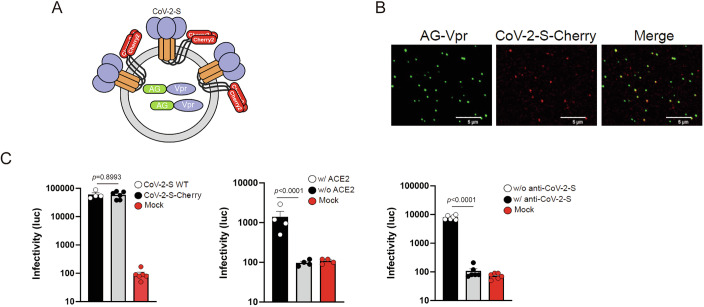
Pseudoviruses carrying CoV-2-S labeled with fluorescent protein retain infectivity. (**A**, **B**) Schematic model of CoV-2-S-mCherry carrying a virion labeled with Vpr-Azami Green (AG) (**A**) and immunofluorescence analysis of virions (**B**). (**C**) Infectivity of unlabeled CoV-2-S or CoV-2-S-Cherry carrying pseudotyped lentivirus in 293T cells, expressing ACE2 and TMPRSS2 (left) without ACE2 (middle) or with anti-CoV-2-S antibody (right); the quantified results from a representative experiment in the two experiments independently conducted are expressed as mean ± SEM, *n* = 4–6. The results of Student’s *t* tests are shown above the bars.

**Figure 3:**
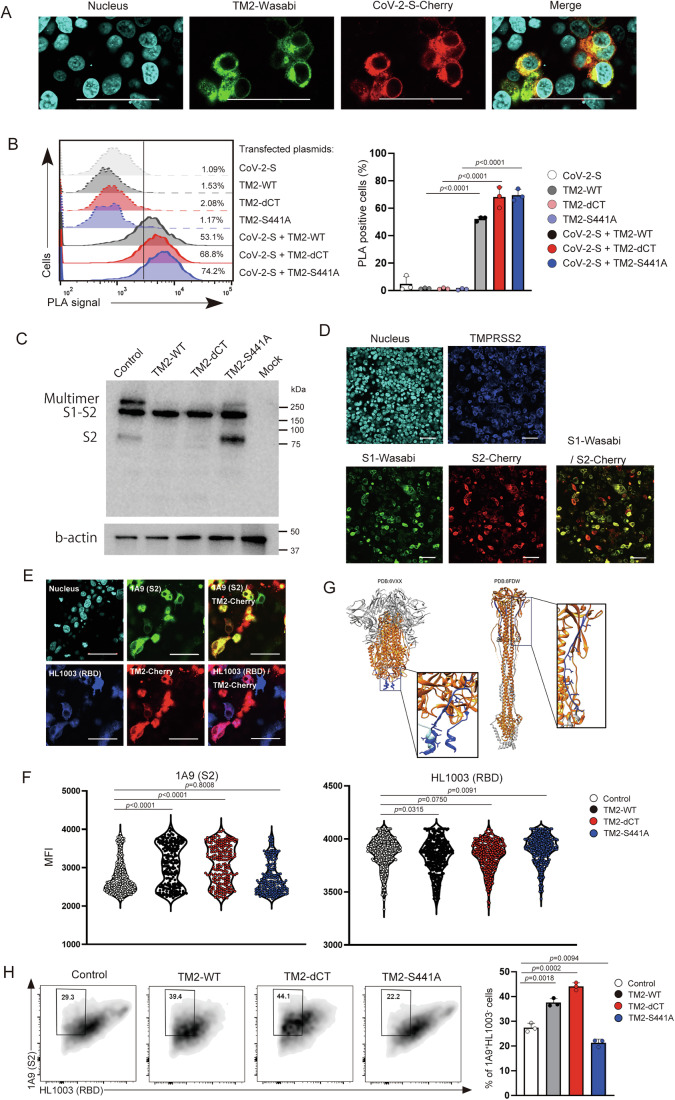
TM2 co-localizes with CoV-2-S and induces CoV-2-S intracellular structural changes. (**A**) Co-localization of TMPRSS2-Wasabi (TM2-Wasabi, green) and CoV-2-S-mCherry (CoV-2-S-Cherry, red) in 293 T cells at 24 h post-transfection. (**B**) Proximity ligation assay (PLA) of CoV-2-S and TMPRSS2-mCherry (TM2-WT), TMPRSS2-dCT-mCherry (TM2-dCT), or TMPRSS2-S441A-mCherry (TM2-S441A) in 293 T cells. The quantified results are expressed as mean ± SEM of three separate experiments (*n* = 3). (**C**) Immunoblotting analysis of CoV-2-S cleavage in 293T cells expressing CD4-mCherry (Control), TMPRSS2-mCherry (TM2-WT), TMPRSS2-dCT-mCherry (TM2-dCT), or TMPRSS2-S441A-mCherry (TM2-S441A). The samples were obtained from the same experiment, and the gels and blots were processed in parallel. (**D**) Fluorescence image analysis of S1-Wasabi (green) and S2-mCherry (red) double-labeled CoV-2-S in 293 T cells expressing TMPRSS2 (blue immunostaining). (**E**) Immunofluorescence analysis for CoV-2-S structural changes using anti-S1 (HL1003, blue) and S2 (1A9, green) antibodies in TM2-WT (red)-expressing 293 T cells at 24 h post-transfection. Low-magnification images are shown in Fig. EV3A. (**F**) A quantitative analysis of 1A9 and HL1003 binding is presented in Fig. EV3A. The mean fluorescence intensity (MFI) of 1A9 or HL1003 signal in mCherry-expressing cells was determined in CoV-2-S- and TM2-mCherry-expressing cells. A total of 214 regions of interest (ROIs) positive for 1A9, as well as 301 ROIs positive for HL1003, were randomly selected from each of the images, which were obtained from three independent staining experiments, and plotted. Representative data from three independent experiments are shown. (**G**) Schematic representation of the structure of the 1A9 epitope (blue) in closed (left, PDB: 6VXX) (Walls et al, 2020) and open (right, PDB: 8FDW) (Shi et al, 2023) forms of CoV-2-S. (**H**) Flow cytometry analysis for CoV-2-S structural change in CD4-mCherry (Control)-, TM2-WT-, TM2-dCT-, or TM2-S441A-expressing 293T cells at 24 h post-transfection. The percentage of 1A9^+^ and HL1003^−^ cells in the mCherry^+^ population was plotted (right, the quantified results are expressed as mean ± SEM of three separate experiments, *n* = 3). Scale bars indicate 50 μm. The results of Student’s *t* tests are shown above the bars. Source data are available online for this figure.

Next, we labeled TM2 and CoV-2-S with fluorescent proteins and expressed them in 293T cells to investigate the interaction between TM2 and CoV-2-S, as well as the subcellular TM2 and CoV-2-S distribution. Fluorescently labeled TM2 and CoV-2-S maintained their functions during viral entry (Figs. EV1A and EV2A–C). We found that TM2 and CoV-2-S co-localized with a high probability in 293 T cells (Fig. 3A). A proximity ligation assay (PLA) also confirmed the proximity between TM2 and CoV-2-S (Fig. 3B).

We hypothesized that the inhibition of CoV-2-S incorporation into virions occurred owing to the intracellular cleavage of CoV-2-S by TM2. However, immunoblotting analysis showed that the majority of CoV-2-S remained uncleaved in TM2-expressing cells (Figs. 1D and  3C). Moreover, even when S1 and S2 were labeled with different fluorescent proteins (Wasabi and Green for S1; mCherry and Red for S2), and TM2 was expressed, these fluorescent proteins remained co-localized in TM2-expressing cells (Fig. 3D). These results indicate that the inhibitory effect of TM2 on CoV-2-S uptake is independent of the intracellular cleavage of CoV-2-S.

**Figure EV3:**
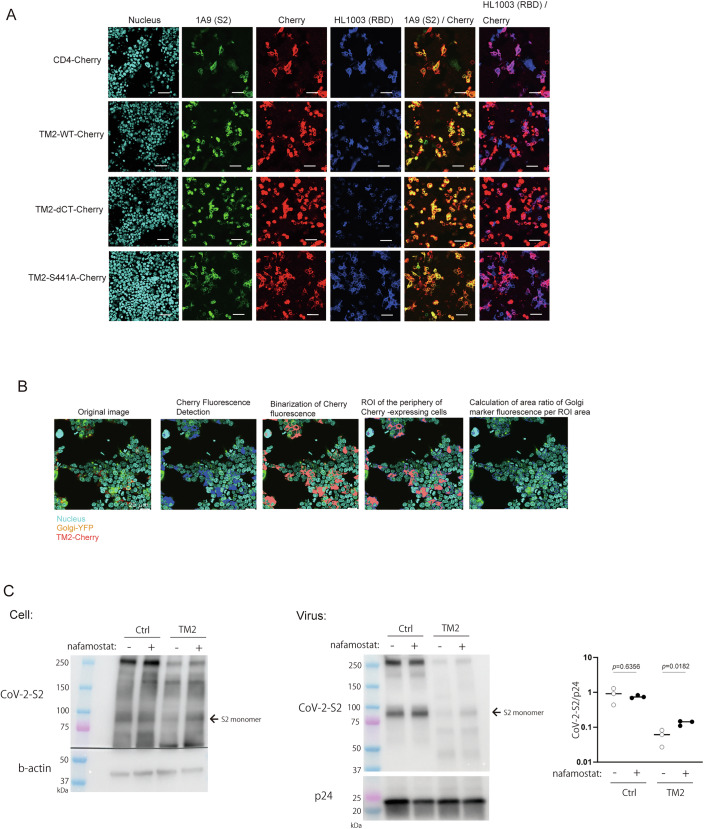
Antibody accessibility to CoV-2-S in TM2-expressing cells. (**A**) Low-magnification image of Fig. 3E. Immunofluorescence analysis of CoV-2-S structural changes using anti-S1 (HL1003) and S2 (1A9) antibodies in CD4-mCherry-, TMPRSS2-WT-mCherry- (TM2-WT)-, TMPRSS2-dCT-mCherry- (TM2-dCT)-, or TMPRSS2-S441A-mCherry- (TM2-S441A)-expressing 293T cells 24 h post-transfection. (**B**) Quantitative analysis of Golgi disruption. The region of interest (ROI) was determined by binarizing the mCherry fluorescence signal and detecting its perimeter to determine the area per TM2-Cherry-expressing cell. Golgi disruption was quantified by calculating the area occupied by Golgi marker fluorescence relative to the ROI area. (**C**) Nafamostat partially rescues the CoV-2-S incorporation into virions. Immunoblotting analysis of cell lysate (left) and CoV-2-S pseudotyped lentiviruses (middle) from 293T cells expressing CD4 (Ctrl), or TM2 with or without 100 μM of nafamostat. The ratio of CoV-2-S2 to p24 was calculated based on band density (right, the quantified results are expressed as mean ± SEM of three separate experiments, *n* = 3). The results of Student’s *t* tests are shown above the bars.

Next, we hypothesized that the reduced incorporation of CoV-2-S into virions is due to conformational changes in CoV-2-S. To examine this, we analyzed the CoV-2-S conformational changes in TM2-expressing cells using two different anti-CoV-2-S antibodies. One of these antibodies, HL1003, recognizes the receptor-binding domain (RBD), whereas the other, 1A9, recognizes the heptad repeat 2 (HR2) region of S2 (Ng et al, 2014). We found that recognition by 1A9 was enhanced, whereas recognition by HL1003 was decreased in TM2-expressing cells (Figs. 3E,F and  EV3A). Based on the reported conformation of CoV-2-S (Shi et al, 2023; Walls et al, 2020), we predicted that the 1A9 epitope would be more easily recognized in its open form, in which S2 would expand towards the target cell membrane (Fig. 3G). The increase in 1A9 recognition upon TM2 expression was confirmed using flow cytometry (Fig. 3H). These results indicated that TM2 expression induced an abnormal intracellular conformational change in CoV-2-S, recognizable by the 1A9 antibody, which reduced the incorporation of viral spikes into virions.

### The Golgi is disrupted in TM2 and CoV-2-S co-expressing cells

**Figure 4:**
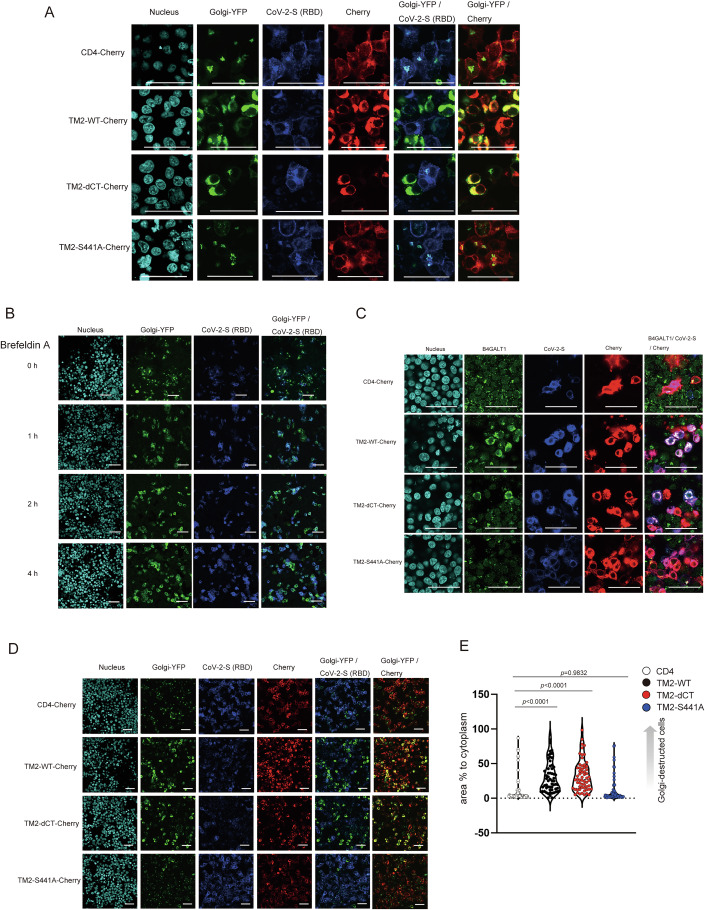
The Golgi apparatus is disrupted in TM2 and CoV-2-S-expressing cells. (**A**) Immunofluorescence analysis for Golgi-YFP (green), CoV-2-S (blue), and TM2-WT, TM2-dCT, or TM2-S441A (red) in 293T cells at 24 h post-transfection. (**B**) Immunofluorescence analysis for 293T cells transfected with Golgi-YFP (green) and CoV-2-S (blue) expression plasmids and treated with 2.5 μg/mL Brefeldin A for the indicated time at 24 h post-transfection. (**C**) Immunofluorescence analysis of endogenous B4GALT1 in 293 T cells transfected with CoV-2-S and CD4-mCherry (CD4-Cherry), TM2-WT-mCherry (TM2-WT-Cherry), TM2-dCT-mCherry (TM2-dCT-Cherry), or TM2-S441A-mCherry (TM2-S441A-Cherry) expression plasmids at 24 h post-transfection. B4GALT1 and CoV-2-S were stained with anti-B4GALT1 (green) and 1A9 (blue) antibodies, respectively. (**D**) Immunofluorescence analysis of Golgi-YFP, CoV-2-S, and CD4, TM2-WT, TM2-dCT, or TM2-S441A-mCherry in 293T cells 24 h post-transfection. Representative data from three independent experiments are shown. (**E**) The percentage of Golgi-YFP area per cytoplasmic area was calculated. The periphery of mCherry-expressing cells was determined by binarizing the mCherry signals, and the percentage of Golgi-YFP per cytoplasm area was calculated (see Fig. EV3B). Sixty cells were randomly selected from at least three independent images and plotted. Representative data from three independent experiments are shown. Scale bars indicate 50 μm. The results of Student’s *t* tests are shown above the bars. Source data are available online for this figure.

Since the viral incorporation of envelope glycoproteins was reduced, we next investigated how the Golgi is involved in CoV-2-S transport and found significant changes in the distribution of the trans-Golgi marker, beta-1, 4-galactosyltransferase 1 (B4GALT1)-conjugated yellow fluorescent protein (Golgi-YFP), in TM2 and CoV-2-S co-expressing cells (Fig. 4A). The changes in the distribution of the Golgi marker were very similar to those induced by Brefeldin A, a specific inhibitor of Golgi trafficking (Fig. 4B) (Fujiwara et al, 1988). Similar changes in the subcellular distribution of B4GALT1 and TM2 were observed with endogenous B4GALT1 expression (Fig. 4C). These results indicate that the co-expression of CoV-2-S and TM2 induced Golgi disruption.

### TM2 by itself induces Golgi disruption

To quantify Golgi distribution, the area of Golgi markers per unit area of each TM2-mCherry-expressing cell line was calculated using image analysis (Fig. EV3B). As the normal Golgi accumulates near the cell nucleus, the Golgi area per cell is small. Abnormal Golgi diffuses throughout the cell, resulting in a large Golgi-marker area. Although SARS-CoV-2 infection has been reported to induce Golgi disruption (Zhang et al, 2025), we found that co-expression of CoV-2-S and TM2-WT or TM2-dCT significantly increased the Golgi-marker area, confirming that the Golgi was disrupted upon co-expression of TM2 and CoV-2-S (*P* < 0.0001, Fig. 4D,E).

**Figure 5:**
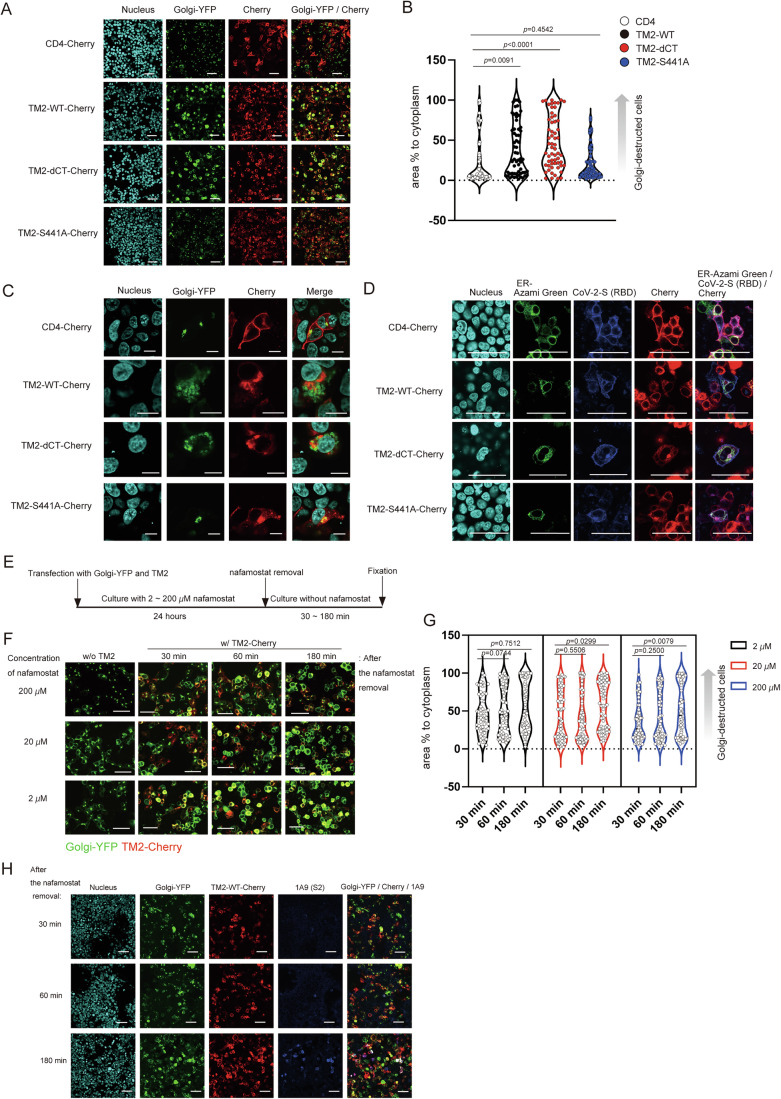
TM2 expression by itself induces Golgi disruption. (**A**) Immunofluorescence analysis of Golgi-YFP (green), TMPRSS2-mCherry (TM2-WT), TMPRSS2-dCT-mCherry (TM2-dCT), and TMPRSS2-S441A-mCherry (TM2-S441A) in 293 T cells at 24 h post-transfection. (**B**) A quantitative analysis of Golgi disruption. The area of Golgi markers relative to the cell area of TM2-expressing cells was calculated using image analysis. Sixty cells were randomly selected from at least three independent images and plotted. Representative data from three independent experiments are shown. (**C**) Immunofluorescence analysis for Golgi-YFP (green) and TM2-WT-, TM2-dCT- or TM2-S441A-mCherry-expression vectors transfected into 293 T cells at 24 h post-transfection. (**D**) Immunofluorescence analysis for ER-Azami Green (green) and TM2-WT, TM2-dCT, or TM2-S441A-mCherry in 293T cells at 24 h post-transfection. (**E**) Experimental scheme for (**F**–**H**). (**F**) 293T cells transfected with Golgi-YFP (green) and TMPRSS2-WT-mCherry (TM2-Cherry, red) expression plasmids and cultured with the indicated concentrations of nafamostat. At 24 h post-transfection, nafamostat was removed by changing the medium, and the cells were cultured for the indicated additional time before fixation. (**G**) A quantitative analysis of Golgi disruption in the transfected 293T cells after the removal of nafamostat. Sixty cells were randomly selected from at least three independent images and plotted. Representative data from three independent experiments are shown. (**H**) Increased 1A9 epitope binding was analyzed in 293 T cells expressing Golgi-YFP, CoV-2-S, and TM2-WT-Cherry at the indicated time points after the nafamostat removal. After removing nafamostat, the cells were cultured for the indicated time before fixation and stained with a 1A9 monoclonal antibody (blue). Scale bars indicate 50 μm. The results of Student’s *t* tests are shown above the bars. Source data are available online for this figure.

Coronavirus spikes and other viral envelope glycoproteins are known to cause ER and Golgi stress (Shaban et al, 2021; Zhang et al, 2025); however, how TM2 affects Golgi function remains unclear. Therefore, we investigated the effects of TM2-only expression in the Golgi, without CoV-2-S expression. Surprisingly, the Golgi was found to be disrupted even in TM2-only-expressing cells (Fig. 5A,B). In addition, confocal microscopy revealed that the Golgi disintegrated into a mesh-like structure in TM2-expressing cells (Fig. 5C), suggesting that its membrane was absorbed by the ER membrane. By contrast, ER morphology remained unaffected in the presence of TM2 (Fig. 5D).

### TM2 enzymatic activity is responsible for trans-Golgi disruption

To examine the dependency on TM2 catalytic activity, we measured the TM2-induced Golgi disruption after removing the TM2 inhibitor nafamostat from TM2-transfected 293T cells. Golgi disruption was suppressed by treatment with nafamostat (Fig. 5E,F). We observed that the Golgi began to disintegrate within 3 h of nafamostat removal (Fig. 5G). These results are consistent with the lack of Golgi disruption with the S441A mutant, which lacks enzymatic activity (Fig. 5A,B), indicating that the TM2 enzymatic activity is required for Golgi disruption. Moreover, we observed partial rescue of CoV-2-S incorporation into lentiviral virions produced in 293 T cells expressing TM2 by the nafamostat treatment (Fig. EV3C).

**Figure 6:**
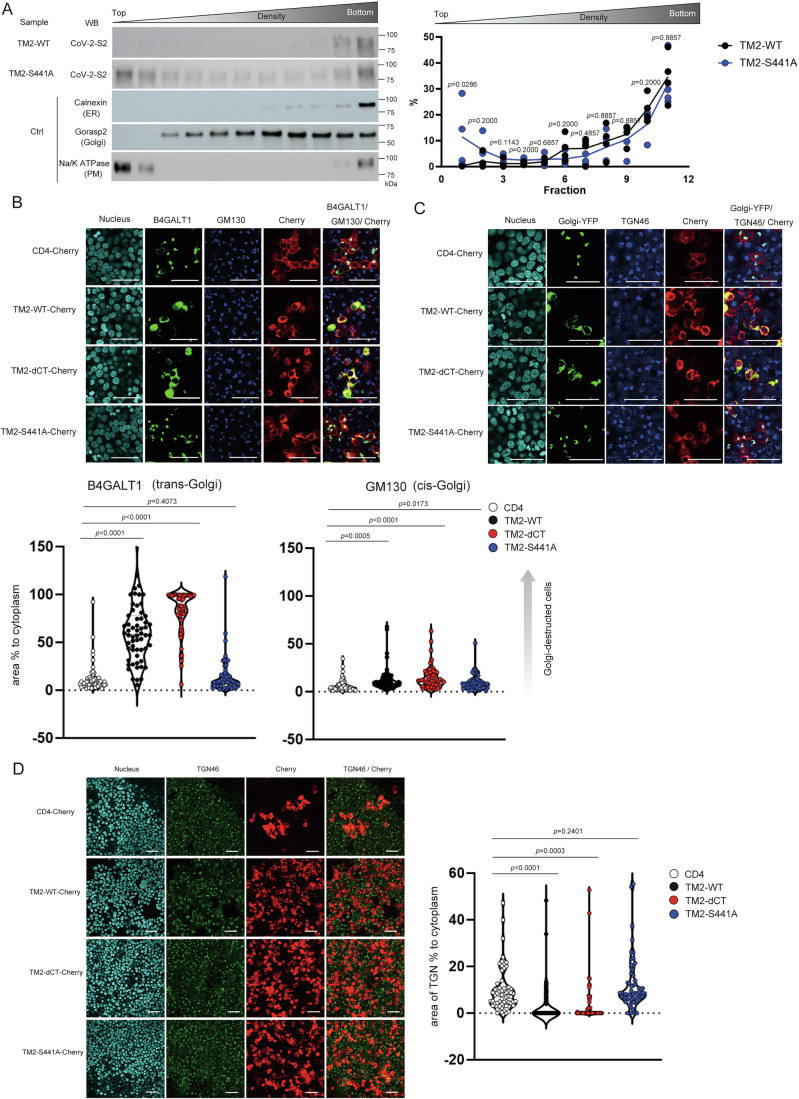
TM2 induces trans-Golgi and trans-Golgi network disruption. (**A**) Immunoblotting analysis of CoV-2-S, Calnexin as an ER marker, Gorasp2 as a Golgi marker, and Na/K ATPase as a marker for plasma membrane in density gradient fraction of 293 T cells expressing TMPRSS2-mCherry (TM2-WT), or TMPRSS2-S441A-mCherry (TM2-S441A) (left). The percentage of CoV-2-S in each fraction is shown in the right panel (the quantified results are expressed as mean ± SEM of four separate experiments, *n* = 4). The samples were obtained from the same experiment, and the gels and blots were processed in parallel. The results of Mann–Whitney tests are shown. (**B**, **C**) Immunofluorescence analysis of GM130 (**B**, blue) and TGN46 (**C**, blue) in 293T cells transfected with Golgi-YFP (B4GALT1, green), CD4-Cherry, TM2-WT-Cherry, TM2-dCT-Cherry, or TM2-S441A-Cherry expression plasmids at 24 h post-transfection. The percentage of B4GALT1 or GM130 area per cytoplasmic area was calculated. Sixty cells were randomly selected from at least three independent images and plotted. Representative data from three independent experiments are shown (**B**, bottom). (**D**) Immunofluorescence analysis of TGN46 (green) in 293T cells transfected with CD4-Cherry, TM2-WT-Cherry, TM2-dCT-Cherry, and TM2-S441A-Cherry expression plasmids at 24 h post-transfection. The percentage of TGN46 area per cytoplasmic area was calculated (right). Sixty cells were randomly selected from at least three independent images and plotted. Representative data from three independent experiments are shown. Scale bars indicate 50 μm. The results of Student’s *t* tests are shown above the bars. Source data are available online for this figure.

Nafamostat treatment also inhibited the TM2-induced CoV-2-S conformational changes, measured by 1A9 accessibility (Fig. 5H), providing further evidence that the observed effects depend on TM2 enzymatic activity. Previous studies have reported that uncleaved CoV-2-S tends to adopt a more “open” structure, resulting in lower incorporation efficiency into viral particles (Nguyen et al, 2021). Therefore, alterations in the intracellular transport of CoV-2-S may affect the 1A9 accessibility. To examine the effect of TM2 enzymatic activity in CoV-2-S intracellular transport, we performed cellular fractionation analysis. Compared to the S441A mutant, TM2-WT showed a significant reduction in CoV-2-S transport to the plasma membrane fraction (Fig. 6A). Therefore, we cannot completely exclude the possibility that alterations in the intracellular transport of CoV-2-S are affecting 1A9 accessibility.

B4GALT1, a Golgi YFP marker, reportedly accumulates in the trans-Golgi (Lee et al, 2009), indicating that TM2 expression disrupts the trans-Golgi. Next, Golgi matrix protein 130 (GM130), which accumulates in the cis-Golgi (Nakamura et al, 1995), was stained to examine the effect of TM2 expression on the cis-Golgi. The results showed that the effect of TM2 on the subcellular localization of GM130 was minor compared to that of B4GALT1 (Fig. 6B). We performed a similar analysis of TGN46 distribution in the trans-Golgi network, which revealed that TGN46 was almost completely absent in TM2-expressing cells (Fig. 6C,D). TM2 reportedly cleaves targets under weakly acidic conditions (Kreutzberger et al, 2022), which is consistent with its activation in the trans-Golgi and trans-Golgi networks, which are weakly acidic environment (Kellokumpu, 2019).

### TM2 disrupts the trans-Golgi via ERK activation

**Figure 7:**
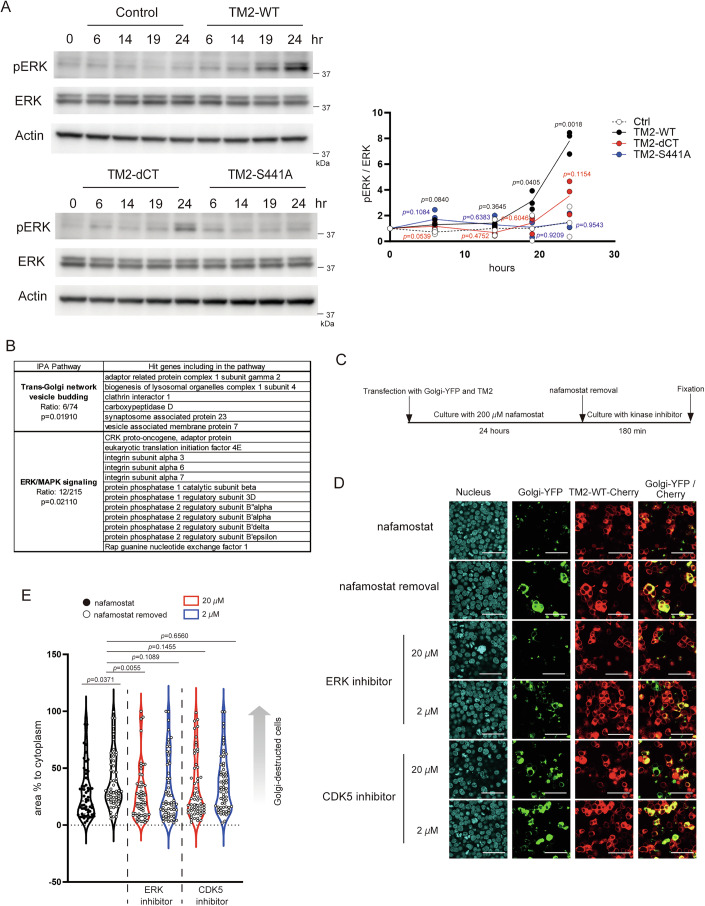
TM2 disrupts the trans-Golgi network via ERK activation. (**A**) Phosphorylated and total ERK (pERK and ERK, respectively) were detected in 293 T cells expressing CD4-mCherry (Control), TMPRSS2-mCherry (TM2-WT), TMPRSS2-dCT-mCherry (TM2-dCT), or TMPRSS2-S441A-mCherry (TM2-S441A) at the indicated time (h) post-transfection by immunoblotting analysis. The ratio of pERK to total ERK at 0 hr was set as 1, and the ratio of pERK to total ERK at each time point to that is shown in the graph (the quantified results are expressed as mean ± SEM of three separate experiments, *n* = 3). The samples were obtained from the same experiment, and the gels and blots were processed in parallel. The results of Student’s *t* tests for the value of Ctrl at each time point are shown above the points. (**B**) IPA analysis of differentially expressed proteins between TM2-WT- and TM2-S441A-transfected 293T cells. (**C**) Experimental scheme for (**D**, **E**). (**D**) Inhibition of TM2-induced Golgi disruption using ERK or CDK inhibitors was analyzed in 293T cells, transfected with Golgi-YFP (green) and TM2-WT (red), and cultured for 24 h in the presence of 200 μM nafamostat. After removing nafamostat, the cells were cultured with the indicated concentrations of ERK or CDK5 inhibitor for 180 min before undergoing fixation. (**E**) Quantitative analysis of the images. Sixty cells were randomly selected from at least three independent images and plotted. Representative data from three independent experiments are shown. Scale bars indicate 50 μm. The results of Student’s *t* tests are shown above the bars. Source data are available online for this figure.

**Figure EV4:**
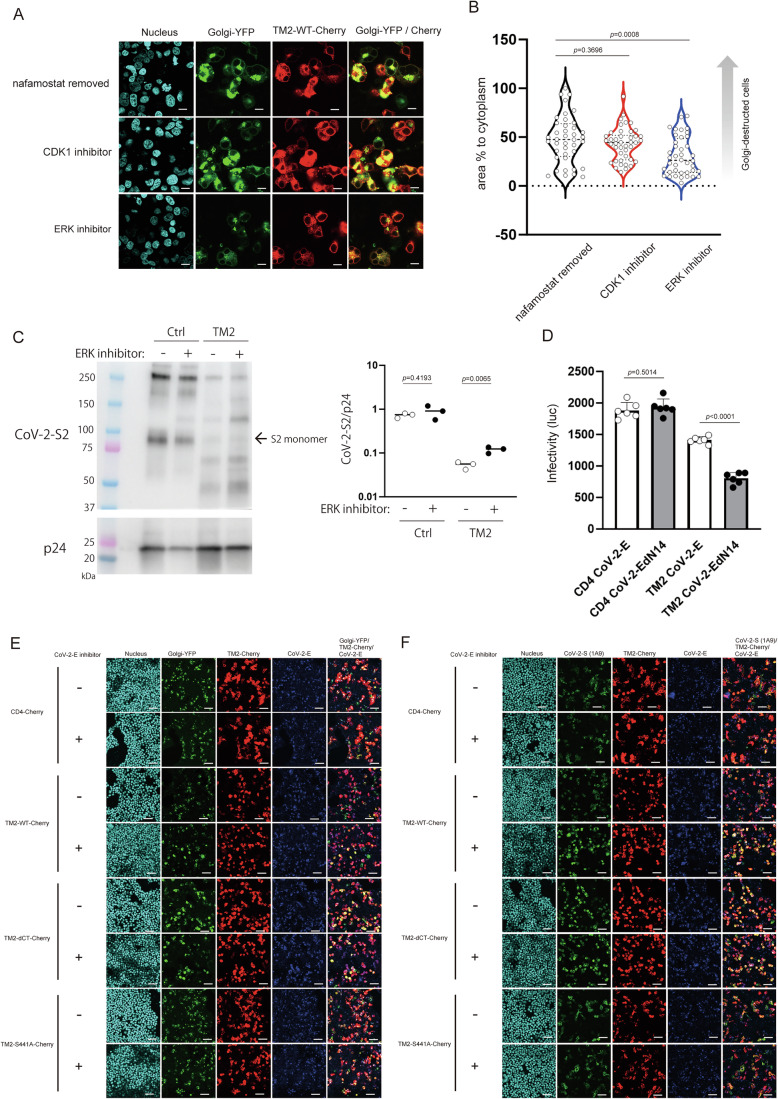
Inhibition of TM2-induced Golgi disruption by an ERK inhibitor. (**A**) Inhibition of TMPRSS2-induced Golgi disruption using ERK or CDK inhibitors, analyzed in 293T cells, transfected with Golgi-YFP (green) and TMPRSS2-WT-mCherry (TM2-WT-Cherry, red) and cultured for 24 h with 200 μM nafamostat. After removing nafamostat, cells were cultured with 20 μM ERK (20-233) or CDK1 (Ro 3306) inhibitors for 180 min before fixation. Scale bars indicate 10 μm. (**B**) Quantitative analysis of Golgi disruption in the images. Forty cells were randomly selected from at least three independent images and plotted. Representative data from two independent experiments are shown. (**C**) Immunoblotting analysis of CoV-2-S pseudotyped lentiviruses in 293T cells expressing CD4 (Ctrl), or TMPRSS2 (TM2) with or without 50 μM of ERK inhibitor. The ratio of CoV-2-S2 to p24 was calculated based on band density (right, the quantified results are expressed as mean ± SEM of three separate experiments, *n* = 3). (**D**) Inhibition of TM2 function by CoV-2-E. Luciferase activity for the infectivity of CoV-2-S pseudotyped lentiviruses produced in CD4-mCherry- (CD4)-, or TMPRSS2-mCherry- (TM2)-, and CoV-2-E or CoV-2-EdN14 expressing 293 T cells (the quantified results from a representative experiment in the two experiments independently conducted are expressed as mean ± SEM, *n* = 6). (**E**) Immunofluorescence analysis of Golgi-YFP (green) and CoV-2-E (blue) in CD4-mCherry (CD4-Cherry)-, TMPRSS2-WT-mCherry (TM2-WT-Cherry)-, TMPRSS2-dCT-mCherry (TM2-dCT-Cherry)-, or TMPRSS2-S441A-mCherry (TM2-S441A-Cherry)-expressing 293T cells cultured with CoV-2-E inhibitory peptide (+) or control peptide (−) at 24 h post-transfection. (**F**) Immunofluorescence analysis for CoV-2-S structural changes using anti-CoV-2-S2 (1A9, green) antibodies in CoV-2-S and CoV-2-E (blue) in CD4-Cherry-, TM2-WT-Cherry, TM2-dCT-Cherry, or TM2-S441A-Cherry (red) -expressing 293 T cells cultured with CoV-2-E inhibitory peptide (+) or control peptide (−) at 24 h post-transfection. Scale bars indicate 50 μm. The results of Student’s *t* tests are shown above the bars.

Golgi disruption is induced by the phosphorylation of the Golgi reassembly and stacking protein (GRASP) 65 and 55 complexes by various kinases, such as extracellular signal-regulated kinase (ERK) and cyclin-dependent kinase (CDK) (Bisel et al, 2008; Feinstein and Linstedt, 2008; Jesch et al, 2001; Sun et al, 2008). In particular, GRASP55, which anchors to the trans-Golgi layers, is primarily regulated by ERK. Activation of ERK releases this anchorage, resulting in trans-Golgi disruption (Bisel et al, 2008). We measured phosphorylated ERK in TM2-transfected 293T cells and observed an increase in phosphorylated ERK in TM2-WT- but not in TM2-S441A-expressing 293T cells (Fig. 7A). To examine the effect of TM2 expression on overall protein synthesis in cells, proteome analysis of TM2-WT-expressing 293T cells was performed and compared with that of TM2-S441A-expressing 293T cells. Ingenuity Pathway analysis (IPA) of 704 protein molecules with significantly different expression levels (*n* = 3 independent analyses, *P* < 0.05) identified hits in pathways related to Golgi membrane trafficking and the ERK pathway (Fig. 7B). We examined the effects of ERK, CDK1, and CDK5 inhibitors on TM2-induced Golgi disruption following nafamostat removal to investigate the mechanism of TM2-induced Golgi disruption. We found that TM2-induced Golgi disruption after nafamostat removal was suppressed by an ERK inhibitor, but not by CDK inhibitors (Figs. 7C–E and  EV4A,B). Furthermore, we observed that ERK inhibitors partially restored the inhibition of CoV-2-S incorporation into lentiviral particles by TM2 (Fig. EV4C). Taken together, these results indicate that TM2 disrupts the trans-Golgi via the ERK pathway.

### SARS-CoV-2 envelope (E) protein inhibits TM2-induced Golgi-disruption

**Figure 8:**
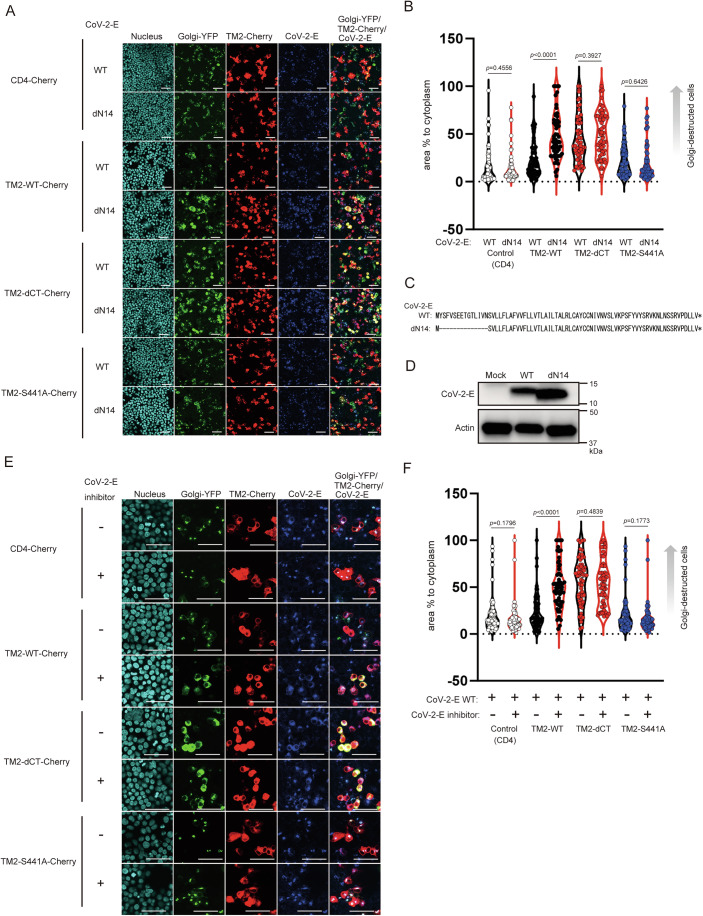
CoV-2-E inhibits TM2-induced Golgi disruption. (**A**) Immunofluorescence analysis of Golgi-YFP (green) and SARS-CoV-2 Envelope protein (CoV-2-E WT or CoV-2-E dN14 mutant, blue) in CD4 (Control)-, TMPRSS2-WT (TM2-WT)-, TMPRSS2-dCT (TM2-dCT)-, or TMPRSS2-S441A (TM2-S441A)-Cherry-expressing 293 T cells at 24 h post-transfection. (**B**) Quantitative analysis of Golgi disruption in Golgi-YFP, TM2, and CoV-2-E WT or dN14-transfected 293 T cells. Sixty cells were randomly selected from at least three independent images and plotted. Representative data from three independent experiments are shown. (**C**, **D**) Amino acid sequence (**C**) and immunoblotting analysis (**D**) of CoV-2-E WT and CoV-2-E dN14 expressed in 293T cells. The samples were obtained from the same experiment, and the gels and blots were processed in parallel. (**E**) Immunofluorescence analysis of Golgi-YFP (green) and SARS-CoV-2 Envelope protein (CoV-2-E, blue) in CD4 (Control)-, TM2-WT-, TM2-dCT-, or TM2-S441A-Cherry-expressing 293T cells cultured with CoV-2-E inhibitory peptide (+) or control peptide (−) at 24 h post transfection. (**F**) Quantitative analysis of Golgi disruption in Golgi-YFP, TM2, and CoV-2-E transfected and CoV-2-E inhibitor-treated 293T cells presented in (**E**) and Fig. EV4E. Sixty cells were randomly selected from at least three independent images and plotted. Representative data from three independent experiments are shown. Scale bars indicate 50 μm. The results of Student’s *t* tests are shown above the bars. Source data are available online for this figure.

Although TM2-induced Golgi disruption compromises the maturation of CoV-2-S, plenty of infectious SARS-CoV-2 virions are released from natural viral-producing cells expressing TM2. Therefore, we investigated the existence of viral molecules that inhibit TM2 function. CoV-2-E plays several roles in the viral life cycle and pathogenesis (Zhou et al, 2023). CoV-2-E is mainly located in the ER-Golgi and increases the pH in the Golgi apparatus (Vargas-Zapata et al, 2022). To examine the effect of CoV-2-E on TM2-mediated Golgi disruption, 293T cells were co-transfected with TM2, CoV-2-E, and Golgi-YFP expression vectors, and Golgi disruption was examined. TM2-mediated Golgi disruption was reduced in cells expressing CoV-2-E (Fig. 8A,B). The N-terminal domain of CoV-2-E is known to be necessary for its oligomerization and function (Bekdash et al, 2024). In a CoV-2-E mutant lacking the 14 amino acids at the N-terminus (CoV-2-E dN14, Fig. 8C,D), the function of suppressing TM2 Golgi disruption was abolished (Fig. 8A,B). We also found that CoV-2-E expression partially rescues infectivity of CoV-2-S pseudotyped lentivirus produced in TM2 expressing 293 T cells (Fig. EV4D). In addition, the CoV-2-E inhibitor iPep-SARS-E (Bekdash et al, 2024) abolished the CoV-2-E-induced suppression of TM2 function during Golgi disruption (Figs. 8E,F and EV4E). Interestingly, we did not observe this effect of CoV-2-E in the TM2-dCT mutant lacking the TM2 cytoplasmic tail, suggesting that the interaction between the TM2 cytoplasmic tail and CoV-2-E is required for the inhibition of TM2 function by CoV-2-E (Fig. 8E,F). Furthermore, we found that CoV-2-E inhibits the TM2-inducing structural change of CoV-2-S detected by increased accessibility of 1A9 (Fig. EV4F). These results indicate that CoV-2-E regulates Golgi function by inhibiting TM2 and suggest that CoV-2-E increases infectious viral production in virus-producing cells via supporting CoV-2-S incorporation into virions.

## Discussion

CoV-2-E reportedly increases CoV-2-S incorporation into virions, thereby enhancing viral infectivity. Here, we show that CoV-2-E inhibits TM2 function, which induces Golgi disruption via the ERK pathway and reduces CoV-2-S incorporation into virions. These results suggest that SARS-CoV-2 utilizes TM2 during the cell entry phase and inhibits TM2 function during the viral-production phase for efficient viral replication. Our data using TM2 inhibitor and TM2 mutant indicate that TM2 enzymatic activity was essential for the inhibition of CoV-2-S incorporation. In this study, we demonstrated the inhibition of CoV-2-S incorporation into virions by TM2 expression, suggesting that TM2 has a protective role in the host against SARS-CoV-2 replication.

Despite the growing interest in TM2 due to its function in envelope glycoprotein cleavage, such as with CoV-2-S and HA, its physiological function remains unclear. Our present study showed that TM2 is not only involved in the inhibition of viral envelope glycoprotein incorporation into virions but also plays a role in the regulation of trans-Golgi function. Therefore, our work sheds light on the physiological function of TM2 in the trans-Golgi, which was entirely unknown. Golgi disruption induced by ERK and CDK activation has been studied with respect to cellular damage in neurons and Alzheimer’s disease (Bisel et al, 2008; Sun et al, 2008). TM2 is highly expressed in the respiratory system, intestinal tract, kidneys, and testes, and its role in membrane protein synthesis in these organs should be examined. The results of our experiments using kinase inhibitors indicate that TM2 disrupts the Golgi via the ERK pathway. Golgi stacking proteins GRASP65 and GRASP55 hold the Golgi membranes together and anchor them to the membranes of cis- and medium-trans-Golgi, respectively. Phosphorylation by kinases induces Golgi disruption, and GRASP55 is phosphorylated by ERK, suggesting that TM2-induced trans-Golgi disruption is mediated by the ERK pathway, which is consistent with other reports (Feinstein and Linstedt, 2008; Jesch et al, 2001).

TM2 expression is reportedly enhanced by both TLR and IL-1β signaling (Cioccarelli et al, 2021; Yao et al, 2022). Therefore, TM2 expression is induced by inflammatory responses during viral infection, leading to the inhibition of viral envelope glycoprotein incorporation into virions. Coronaviruses that utilize TM2 for cell entry, such as SARS-CoVs and Middle East Respiratory Syndrome (MERS-CoV), might have evolved to use increased TM2 during the inflammatory response to enter new target cells and expand infection. On the other hand, TM2 reduces the production of infectious SARS-CoV-2 as we have shown here. Thus, inhibition of the TM2 function by CoV-2-E in the viral production phase is important for SARS-CoV-2 replication.

The fact that TM2 inhibited CoV-2-S, HIV-1 Env, and SIV Env incorporation into the virion suggests that the inhibitory effect of TM2 on CoV-2 was not dependent on the cleavage of CoV-2-S in the cell; indeed, most CoV-2-S remains uncleaved. This suggests that TM2 acts as a restriction factor not only for CoV-2-S but also for the biosynthesis of a wide range of viral envelope glycoproteins. Here, we found that TM2 expression in HIV-1- and SIV-producing cells severely reduced the infectivity of these viruses. Since the expression of TMPRSS family proteins has also been reported in T cells, monocytes, and placental cells (Agostinis et al, 2022; Yao et al, 2022), the involvement of TMPRSS family proteins in HIV-1 replication will be a subject for future studies.

## Methods

**Table:** Reagents and tools table

Reagent/resource	Reference or source	Identifier or catalog number
**Experimental models**		
SARS-CoV-2 (WK-521)	JIHS, NIID, ARC, Tokyo, Japan	408667
Simian immunodeficiency virus (SIV)	JIHS, NIID, ARC, Tokyo, Japan	M33262
HIV-1 (NL4_3, AD8)	JIHS, NIID, ARC, Tokyo, Japan	AF324493.2 AF004394.1
HEK 293T	ATCC	CRL-11268
Calu-3	ATCC	HTB-55
Caco-2	RIKEN BRC through the National BioResource Project of the MEXT	RCB0988
Vero E6 TMPRSS2	NIBN JCRB Cell Bank	JCRB1819
Human ACE2-expressing A549	InvivoGen	a549-hace2
COS-1	RIKEN BRC	RCB0143
TZM-bl	ATCC	CRL-8129
PM1-R5	Dr Yasuko Tsunetsugu-Yokota (Yamamoto et al, 2009)	
Jurkat	RIKEN BRC	RCB3052
**Recombinant DNA**		
pcDNA3	Thermo Fisher Scientific	
pBApo-EF1	Takara Bio	
pFC14K	Promega	
TMPRSS2 cDNA	Sino Biological	HG13070-UT
CoV-2-S	Takeshita et al, 2023	
CoV-2-E	This study	
CD4 (CD4-mCherry)	This study	
TM2-WT (TMPRSS2-mCherry)	This study	
TM2-dCT (TMPRSS2-dCT-mCherry)	This study	
S441A (TMPRSS2-S441A-mCherry)	This study	
Wasabi	Addgene	ABW74902.1
mCherry	Addgene	165828
CoV-2-S-mCherry (CoV-2-S-Cherry)	This study	
Golgi-YFP (beta-1, 4-galactosyltransferase 1 (B4GALT1)-conjugated yellow fluorescent protein)	Addgene	56590
TMPRSS2-Wasabi (TM2-Wasabi)	This study	
pCMV delta R8.2	Addgene	12263
pLentiLuc	Urano et al, 2008	
pVSV-G	Addgene	138479
pCMV3-ACE2	Sino Biological	HG10108-UT
pBRmac239	JIHS, NIID, ARC, Tokyo, Japan	M33262
pNL-E	Dr Yasuko Tsunetsugu-Yokota (Yamamoto et al, 2009)	
pNLAD8-D	Dr Yasuko Tsunetsugu-Yokota (Yamamoto et al, 2009)	
**Antibodies**		
Anti-CoV-2-S1 RBD (HL1003)	GeneTex	GTX635792
Anti-SARS-CoV-2-S2 (1A9)	GeneTex	GTX632604
Anti-SARS-CoV-2-nucleoprotein	GeneTex	GTX135357
Anti-GM130	Proteintech	11308-1-AP
Anti-SARS-CoV-2-S2	GeneTex	GT745
Anti-Calnexin	CST	2433
Anti-GORASP2	Proteintech	10598-1-AP
Anti-B4GALT1	Abcam	ab121326
Anti-TGN46	Proteintech	13573-1-AP
Anti-SARS-CoV-2 Envelope	GeneTex	GTX136046
Anti-rabbit IgG-A488	Abcam	ab150077
Anti-rabbit IgG-A647	Abcam	ab150079
Anti-mouse IgG-A647	BioLegend	405322
Anti-mouse IgG-A647	Abcam	ab150115
Anti-mCherry antibody	Abcam	ab167453
Anti-SARS-CoV-2-S1 antibody (HL6)	GeneTex	GTX635654
Anti-beta-actin antibody	MBL	M177-3
Anti-HIV-1 p24 antibody (Nu24)	Terahara et al, 2011	
Anti-HIV-1 gp120 antibody	Abcam	ab21179
Anti-HIV-1 Env plasma pooled from HIV-1 positive individuals	JIHS, NIID, ARC, Tokyo, Japan	
Anti-p-ERK	Cell Signaling	4370
Anti-ERK	BioLegend	686901
Anti-mouse IgG-HRP	MBL	330
Anti-rabbit IgG-HRP	MBL	458
Anti-human IgG-HRP	Promega	W4031
Anti-TMPRSS2	Abcam	ab109131
**Oligonucleotides and other sequence-based reagents**		
Vector construction		
pCMV3-hCD4-mCherry2 (CD4-Cherry)		
CD4_Cherry2_F1	Operon	CCAGTGTCCTCACCGGTTTCAGAAGACATGTAGCCCCATTGGCGGAGGCGGATCCGGATCCATGGTGAGC
CD4_Cherry2_R1	Operon	GCACCAGAAGCAAGTGCCTAAAAGGGACTCCCCGGTTCATGGTACCAAGCTTGGTGGCGGCCCCTATAGT
CD4_F1	Operon	ATGAACCGGGGAGTCCCTTTTAGG
CD4_R1	Operon	AATGGGGCTACATGTCTTCTGAAACCG
pCMV3-hTMPRSS2-mCherry2 (TM2-WT-Cherry)		
hTMPRSS2_F1	Operon	GGCCGCCACTCCACCGGCGGCATGGACGAGCTGTACAAGTAATAATCTAGAGCGGCCGCCGAATTCGGGC
hTMPRSS2_R1	Operon	CTCCTCGCCCTTGCTCACCATGGATCCGGATCCGCCTCCGCCGCCGTCTGCCCTCATTTGTCGATAAATC
GS_mCherry2_F1	Operon	GGCGGAGGCGGATCCGGATCCATGGTGAGCAAGGGCGAGGAGGATAACATG
mCherry2_R2	Operon	TTCTTACTTGTACAGCTCGTCCATGCCGCCGGTGGA
pCMV3-hTMPRSS2-mWasabi2 (TM2-WT-Wasabi)		
TMPRSS2Vector_F1	Operon	TAATAAACTCGAGTCTAGAGCGGCCGCCGAATTCG
hTMPRSS2_R1	Operon	CTCCTCGCCCTTGCTCACCATGGATCCGGATCCGCCTCCGCCGCCGTCTGCCCTCATTTGTCGATAAATC
TMPRSS2_Wasabi2_F1	Operon	CAAATGAGGGCAGACGGCGGCGGAGGCGGATCCGGATCCATGGTGAGCAAGGGCGAGGAGACCACAATG
TMPRSS2_Wasabi2_R1	Operon	GGCCCGAATTCGGCGGCCGCTCTAGACTCGAGTTTATTACTTGTACAGCTCGTCCATGCCGTCGGTGGA
pCMV3-hTMPRSS2dCT-mCherry2 (TM-dCT-Cherry)		
TMPRSS2Vector_F1	Operon	TAATAAACTCGAGTCTAGAGCGGCCGCCGAATTCG
TMPRSS2_dCT_R1	Operon	AGGGTCAAGGTGATGCACAGTGCTTTCTTAGTCTTTGACCCTGAGTTCAAAGCCATGGTACCAAGCTTGG
TMPRSS2dCT_F1	Operon	TCAAAGACTAAGAAAGCACTGTGCATCACC
hTMPRSS2_R1	Operon	CTCCTCGCCCTTGCTCACCATGGATCCGGATCCGCCTCCGCCGCCGTCTGCCCTCATTTGTCGATAAATC
TMPRSS2dTM_F1	Operon	GGCGGCGGAGGCGGATCCGGATCCATG
mCherry_Vec_R3	Operon	CGGGCCCGAATTCGGCGGCCGCTCTAGACTCGAGTTTATTACTTGTACAGCTCGTCCATGCCGCCGGTGGAGTGG
pCMV3-hTMPRSS2-S441A-mCherry2 (TM2-S441A-Cherry)		
TMPRSS2Vector_F1	Operon	TAATAAACTCGAGTCTAGAGCGGCCGCCGAATTCG
Vec_TMPRSS2_R3	Operon	AAGGTCCAATAGCTGGTGGTGACCCTGAGTTCAAAGCCATGGTACCAAGCTTGGTGGCGGCCCCTATAGTGAGTCG
hTMPRSS2_F1	Operon	GGCCGCCACTCCACCGGCGGCATGGACGAGCTGTACAAGTAATAATCTAGAGCGGCCGCCGAATTCGGGC
S441A_R1	Operon	ATTGTTCTTCGAAGTGACCAGAGGCCCTCCAGCGTCACCCTGGCAAGAATCGACGTTCCCCTG
S441A_F1	Operon	CAGGGGAACGTCGATTCTTGCCAGGGTGACGCTGGAGGGCCTCTGGTCACTTCGAAGAACAAT
hTMPRSS2_R1	Operon	CTCCTCGCCCTTGCTCACCATGGATCCGGATCCGCCTCCGCCGCCGTCTGCCCTCATTTGTCGATAAATC
TMPRSS2dTM_F1	Operon	GGCGGCGGAGGCGGATCCGGATCCATG
mCherry_Vec_R3	Operon	CGGGCCCGAATTCGGCGGCCGCTCTAGACTCGAGTTTATTACTTGTACAGCTCGTCCATGCCGCCGGTGGAGTGG
pBApo-EF1-CoV-2-S-mCherry2 (CoV-2-S-Cherry)		
CoV2SSino_f2	Operon	ATGTTTGTGTTCCTGGTGCTGCTGCCACTG
CoV2S_R3	Operon	TTTCACTCCTTTCAGCACAGGTTCAGAGTCATCCTCATCA
CoV2S_Linker_F1	Operon	TGATGAGGATGACTCTGAACCTGTGCTGAAAGGAGTGAAAGAGAGCGGCAGCGTGTCCAGCGAACAGCTG
CoV2TMrevmCherry2_R1	Operon	TCTGTCTTTTTATTGCCGTCATAGCGCGGGTTCCTTCTTACTTGTACAGCTCGTCCATGCCGCC
pBA_CoV2f2	Operon	GAAGGAACCCGCGCTATGACGGCAATAAAAAGACAGAATAAAACGCACGGTGTTGGGTCGTTTGTTCATAAACGCGG
pBA_CoV2R3	Operon	GGCTGGACACCAGTGGCAGCAGCACCAGGAACACAAACATGAAGAAAAAAACTTTGAACCACTGTCTGAGGC
pBApo-EF1-CoV-2-S1wasabi-S2-Tmlink-Cherry2 (CoV-2-S1-Wasabi-S2-Cherry)		
Site1_R1	Operon	CTCGCCCTTGCTCACCATGGATCCGGATCCGCCTCCGCCAGACACATGGATGGCATGGAACCAGGTCAC
pBA_CoV2f2	Operon	GAAGGAACCCGCGCTATGACGGCAATAAAAAGACAGAATAAAACGCACGGTGTTGGGTCGTTTGTTCATAAACGCGG
Site1_Wasabi_F2	Operon	GCGGATCCGGATCCATGGTGAGCAAGGGCGAG
Wasabi_Linker_R2	Operon	CCTCTTGGTGCCATTGGTGCCGCTGTCCAGGCTTCTAAATTGTGCGAGTTGCTCGGAGCTGACGGACCCGGATTCGGACACGCTGCCGCTCTCCTTGTACAGCTCGTCCAT
Site1_linker2_F1	Operon	ATTTAGAAGCCTGGACAGCGGCACCAATGGCACCAAGAGGTTTGACAACCCTGTGCTGCC
CoV2S_R3	Operon	TTTCACTCCTTTCAGCACAGGTTCAGAGTCATCCTCATCA
CoV2S_Linker_F1	Operon	TGATGAGGATGACTCTGAACCTGTGCTGAAAGGAGTGAAAGAGAGCGGCAGCGTGTCCAGCGAACAGCTG
CoV2TMrevmCherry2_R1	Operon	TCTGTCTTTTTATTGCCGTCATAGCGCGGGTTCCTTCTTACTTGTACAGCTCGTCCATGCCGCC
pFC14K-CoV-2-E (CoV-2-E)		
Golgi_F4	Operon	AATTCTAGAGTCGACCTGCAGGCATGCAAGCTG
FC14K_R1	Operon	GTGGATTCCTTACTTATTCCATGGCGATCGCTAGCCCTATAG
CoV2E_F2	Operon	ATAGGGCTAGCGATCGCCATGGAATAAGTAAGGAATCCACGCCACCATGTATTCCTTCGTGAGCGAAGAGAC
Eprotein_R1	Operon	GCCGGATCAGCTTGCATGCCTGCAGGTCGACTCTAGAATTATCACACCAGCAGGTCAGGCACGCGGGAAGA
pFC14K-CoV-2-E-dN14 (CoV-2-E dN14)		
E_Halo_F1	Operon	AATCTGAATTCTTCCCGCGTGCCTGACCTGCTGGTGGGAAGCAGCGGATCCGAAATCGGTACTGGCTTTCCATTCGACC
FC14K_R1	Operon	GTGGATTCCTTACTTATTCCATGGCGATCGCTAGCCCTATAG
CoV2E_F3	Operon	CTAGCGATCGCCATGGAATAAGTAAGGAATCCACGCCACCATGAGCGTGCTGCTGTTTCTGGCCTTTGTGGTG
CoV2E_R1	Operon	GGATCCGCTGCTTCCCACCAGCAGGTCAGGCACGCGGGAAGAATTCAG
q-PCR		
TMPRSS1		
TM1_Tq_F1	Operon Operon	cactccgagctggacgtgcgaacgg
TM1_Tq_Pr1	Operon	FAM-cgccaatggcacgtcgggcttcttctgt-TAM
TM1_Tq_R1	Operon	gatggcggccaagaaacggcctctg
TMPRSS2		
TM2-3-Fw	Operon	gcagtggtttctttacgctgt
TM2_C3_Tq1	Operon	FAM-tcaacttgaactcaagccgccagagcagga-TAM
TM2-3-Re	Operon	ccgcaaatgccgtccaatg
TMPRSS4		
TM4_Tq_F1	Operon	tgattctggataaatactacttcctctgcgggcagcc
TM4_Tq_Pr1	Operon	FAM-tccacttcatcccgaggaagcagctgtgtgac-TAM
TM4_Tq_R1	Operon	cacctgcagtgtggatcggtccttgg
TMPRSS11D		
TM11D_Tq_F1	Operon	gtggatcctgacagcagctcactgcttcag
TM11D_Tq_Pr1	Operon	FAM-tctaatcctcgtgactggattgccacgtctgg-TAM
TM11D_Tq_R1	Operon	ggaagccactgatgggaggacccagagtagat
TMPRSS13		
TM13_Tq_F1	Operon	agctgtcccaagcacgctgttcgctgtgac
TM13_Tq_Pr1	Operon	FAM-tggtggactgcaagctgaagagtgacgagct-TAM
TM13_Tq_R1	Operon	ggaagccactgatgggagacccagagtagat
b-actin		
hActb_F1	Operon	cccaaggccaaccgcgagaa
hActb_Tq1	Operon	FAM-tgacccagatcatgtttgagaccttcaacaccc-TAM
hActb_R1	Operon	cgtcaccggagtccatcacga
HIV-1 (lentivirus vector)		
Gag183UF	Operon	ctagcagtggcgcccgaacag
Gag187P-MGB	Operon	FAM-tctctcgacgcaggactcggcttgctg-TAM
Gag187LR	Operon	ccatctctctccttctagcctccgctagtca
**Chemicals, enzymes, and other reagents**		
NEBuilder^®^ HiFi DNA Assembly Cloning Kit	NEB	E5520
Brefeldin A	Sigma	B6542
iPep-SARS-E	Bekdash et al, 2024	
DMEM	Thermo Fisher Scientific	11965-092
Fetal bovine serum	Sigma	A8412
cover glass 8-well chambers	AGC	5232-008
poly-L-Lysine	Sigma	P4832
96-well plate	Falcon	353072
FuGene 6	Promega	E2692
Formaldehyde	Thermo Fisher Scientific	28908
HBSS	Thermo Fisher Scientific	14175-095
goat serum	Abcam	ab7481
saponin	Sigma	S7900
Hoechst 33342	FujiFilm Wako	346-07951
0.45-μm filter	Millipore	SLHVR33RB
HIV-1 p24 ELISA kit	ZeptoMetrix	0801008
Glo lysis buffer	Promega	E2661
Bright-Glo luciferase assay system	Promega	E2620
carboxymethyl cellulose sodium salt	FujiFilm Wako	039-01335
neutral buffered formalin	Muto Chemical	20214
crystal violet	Fujifilm Wako	038-04862
Hygromycin B	Thermo Fisher Scientific	10687010
RPMI	Thermo Fisher Scientific	11875093
SARS-CoV-2 nucleocapsid protein (NP) ELISA	Proteintech	KE30007
SE kit	Lonza	V4XC-1024
nafamostat	FujiFilm Wako	141-08701
Dynabeads Intact Virus Enrichment	Thermo Fisher Scientific	10700D
SIV p27 Antigen Capture Assay kit	ABL	5436
Lipofectamine LTX with Plus reagent	Thermo Fisher Scientific	15338030
Zombie Aqua Fixable Viability Kit	BioLegend	423101
BD Cytofix/Cytoperm	BD Biosciences	554723
BD Perm/Wash	BD Biosciences	554723
Mojo Sort buffer	BioLegend	480017
Sucrose	Sigma	S0389-500G
RIPA lysis buffer	Nacalai tesque	16488-34
cOmplete proteinase inhibitor	Roche	05892953001
Protease inhibitor	Roche	1187358001
Immobilon-P PVDF membranes	Millipore	IPVH00010
Trans-Blot Turbo Mini PVDF Membrane, 0.2 µm	Bio-Rad	12023954
Phosphatase Inhibitor Cocktail	Nacalai tesque	07575-51
Immobilon HRP substrate	Millipore	WBKLS0500
Duolink® flowPLA Detection Kit - FarRed	Sigma-Aldrich	DUO94004-40TST
PFA	Thermo Fisher Scientific	28908
Tris-HCl(pH8.5)	FujiFilm Wako	316-90405
Urea	Supelco. 8 M Sigma-Aldrich 500 g	U4883-6X25ML U0631-500G
TCEP	FujiFilm Wako 1 g Sigma-Aldrich 2 g	575-47161 C4706-2G
CAA	FujiFilm Wako	032-09762
Bicinchoninic acid (BCA) assay	Thermo Fisher Scientific	23225
Ammonium bicarbonate	FUJIFILM Wako	579-34935
Lys-C	FUJIFILM Wako	125-05061
Trypsin	Promega	V5280
ERK inhibitor	Cayman Chemical	15944
CDK5 inhibitor 20-233	MCE	HY-123772
CDK1 inhibitor Ro 3306	Abcam	ab141491
EDTA	Sigma-Aldrich	20-158
OptiPrep	Sigma	D1556
puromycine	Thermo Fisher Scientific	A1113803
TRIzol	Thermo Fisher Scientific	15596026
Direct-zol RNA Kit	Zymo Research	R2052
QuantiTect RT-PCR Kit	Qiagen	20445
**Software**		
CellSens Dimension software	Evident	
ImageJ	Schneider et al, 2012	
FlowJo	BD Biosciences	
DIA-NN (v.1.8.1)	PMID 31768060	
MaxQuant	PMID 27809316	
UCSF Chimera software	Pettersen et al, 2004	
Prism 8.4.3 software	GraphPad	
**Other**		
FLUOVIEW FV3000 system	Evident	
Keyence BZ-9000 system	Keyence	
GloMax Discover Microplate Reader	Promega	
Nucleofector	Lonza	
FLUOVIEW FV3000 with an OSR system	Evident	
FACSCanto II	BD Biosciences	
Mini-PROTEAN system	Bio-Rad	
Trans-Blot® Turbo™ System	Bio-Rad	
ChemiDoc system	Bio-Rad	
FUSION-FX6.EDGE V.070	Vilber Bio Imaging	
Orbitrap Eclipse mass spectrometer	Thermo Fisher Scientific	
FAIMSpro interface and combined with a Vanquish Neo UHPLC pump	Thermo Fisher Scientific	
Ingenuity Pathway Analysis	Qiagen	
Optima L-100XP	Beckman Coulter	
SW60Ti	Beckman Coulter	335650
SW41Ti	Beckman Coulter	331362
CFX Maestro Ver. 2.3	Bio-Rad	

### Plasmid expression vectors

All expression plasmids in this study were generated using the NEBuilder HiFi DNA Assembly system (NEB, Ipswich, MA, USA), and the primers used are listed in the Reagents & Tools Table. TM2 cDNA (HG13070-UT) was purchased from Sino Biological (Beijing, China). pcDNA3 (Thermo Fisher Scientific, Waltham, MA, USA), pBApo-EF1 (Takara Bio, Shiga, Japan), and pFC14K (Promega, Madison, WI, USA) were used as vector backbones.

### Fluorescence immunocytochemistry

HEK 293T (293T) cells were cultured in DMEM (11965-092, Thermo Fisher Scientific), supplemented with 10% fetal bovine serum (FBS), on cover glass 8-well chambers (5232-008, AGC Techno Glass, Tokyo, Japan), coated with poly-L-Lysine (PLL, P4832, Sigma, St Louis, MO, USA), and were transfected with CoV-2-S, CoV-2-E, CD4, TM2, and/or Golgi-YFP (a gift from Michael Davidson, Addgene#56590) (Olenych et al, 2007) expression plasmids using FuGene 6 (E2692, Promega) according to the manufacturer’s recommendations. The cells were fixed in 2% formaldehyde (Thermo Fisher Scientific) in HBSS (14175-095, Thermo Fisher Scientific) for 10 min and washed with HBSS at 24 h post-transfection. The fixed cells were permeabilized and blocked in HBSS containing 10% FBS, 3% goat serum (ab7481; Abcam, Cambridge, UK), and 0.2% saponin (S7900; Sigma) for 10 min at room temperature (RT). Cells were stained with anti-CoV-2-S1 RBD (HL1003, GTX635792, 1:300 dilution, GeneTex, Irvine, CA, USA), anti-SARS-CoV-2-S2 (1A9, GTX632604, 1:300 dilution, GeneTex), anti-GM130 (11308-1-AP, Proteintech, Rosemont, IL, USA, 1:500 dilution), anti-B4GALT1 (ab121326, Abcam, 1:500 dilution), anti-TGN46 (13573-1-AP, Proteintech, 1:300 dilution), and anti-SARS-CoV-2 envelope (E) protein (136046, GeneTex, 1:500 dilution) antibodies in HBSS, supplemented with 10% FBS, 3% goat serum, and 0.2% saponin for 12 h at 4 °C. Primary antibodies were detected using anti-rabbit IgG-A488 (ab150077, Abcam, 1:500 dilution), anti-rabbit IgG-A647 (ab150079, Abcam, 1:500 dilution), and anti-mouse IgG-A647 (405322, BioLegend, 1:500 dilution) in HBSS containing 10% FBS, 3% goat serum, and 0.2% saponin for 30 min at RT. Nuclei were stained with Hoechst 33342 (346-07951, FujiFilm Wako, 1:1000 dilution) in HBSS for 10 min at RT. Stained cells were imaged using a FLUOVIEW FV3000 system (Evident, Tokyo, Japan) or a Keyence BZ-9000 system (Keyence, Osaka, Japan). Image analysis was performed using the CellSens Dimension software (Evident) and ImageJ (Schneider et al, 2012).

### Pseudotyped virus infection

293T cells were transfected with TM2-mCherry, pCMV delta R8.2, a gift from Didier Trono (Addgene plasmid # 12263; http://n2t.net/addgene:12263; RRID: Addgene_12263), pLentiLuc (Urano et al, 2008), CoV-2-S (Takeshita et al, 2023), or VSV-G (Addgene_138479) plasmids using FuGene 6. After 6 h, the cells were washed and cultured for another 48 h in DMEM containing 10% FBS. Culture supernatants were filtered through a 0.45-μm filter (Millipore, Burlington, MA, USA), and the amount of p24 was titrated using an HIV-1 p24 ELISA kit (ZeptoMetrix, Buffalo, NY, USA). 293 T cells were transfected with ACE2 (HG10108-UT, Sino Biological) and/or TM2-mCherry expression plasmids using FuGene 6 in 96-well plates and cultured with viruses normalized by p24 amount. After 24 h of culture, luciferase activity in the infected cells was determined using the Glo lysis buffer, Bright-Glo luciferase assay system, and GloMax Discover Microplate Reader (Promega).

### Plaque-forming assay

Vero E6 TMPRSS2 cells (JCRB1819; JCRB Cell Bank, NIBIOHN, Japan) were infected with SARS-CoV-2 (WK-521; NIID, Japan), diluted in DMEM containing 2% FBS. After 2 h of incubation at 37 °C in 5% CO_2_, unbound viruses were removed, and cells were cultured in DMEM containing 2% FBS and 1.0% carboxymethyl cellulose sodium salt (039-01335, FujiFilm Wako, Tokyo, Japan) for 48 h at 37 °C in 5% CO_2_. After fixation with 10% neutral buffered formalin (20214, Muto Chemical, Tokyo, Japan), the cells were stained with 0.1% crystal violet (038-04862, Fujifilm Wako) in 20% ethanol.

### A549-ACE2-TM2 cells

Human ACE2-expressing A549 cells were purchased from InvivoGen (San Diego, CA, USA) and transfected with the TM2-mCherry expression vector using FuGene 6. Transfected cells were selected and cultured with 500 μg/mL of Hygromycin B (Thermo Fisher Scientific). Cells were infected with SARS-CoV-2 (WK-521; NIID, Japan), diluted in RPMI containing 2% FBS with an MOI of 1.0. After 2 h of incubation at 37 °C in 5% CO_2_, unbound viruses were removed, and cells were cultured in RPMI containing 2% FBS for 96 h at 37 °C in 5% CO_2_. Viruses in culture supernatant were evaluated by a SARS-CoV-2 nucleocapsid protein (NP) ELISA (Proteintech, Rosemont, IL, USA) and used (8–800 pg of NP per well) to infect Vero E6 TMPRSS2 cells for the plaque-forming assay described above.

### Pseudotyped virus production in Calu-3 cells

Calu-3 cells (HTB-55, ATCC) were transfected with TM2-mCherry, pCMV delta R8.2, pLentiLuc, and CoV-2-S plasmids, using Nucleofector (Lonza, Basel, Switzerland) with the SE kit and EO-120 protocol (Lonza) and cultured for 72 h in DMEM containing 10% FBS with 20–80 μM of nafamostat (141-08701, FujiFilm Wako). Culture supernatants were filtered through a 0.45-μm filter and infected into 293 T cells, transfected with human ACE2 using FuGene 6 in 96-well plates. After 48 h of culture, luciferase activity in the infected cells was determined using the Glo lysis buffer, Bright-Glo luciferase assay system, and GloMax Discover Microplate Reader (Promega).

### SARS-CoV-2 production in nafamostat-treated Caco-2 cells

Caco-2 cells were provided by the RIKEN BRC through the National BioResource Project of the MEXT, Japan. Cells were infected with SARS-CoV-2 (WK-521; NIID, Japan), diluted in RPMI containing 2% FBS with an MOI of 0.3. After 2 h of incubation at 37 °C in 5% CO_2_, unbound viruses were removed, and cells were cultured in RPMI containing 2% FBS for 96 h at 37 °C in 5% CO_2_. Viruses in culture supernatant were isolated with Dynabeads Intact Virus Enrichment (Thermo Fisher Scientific) according to the manufacturer’s instructions. Isolated viruses were evaluated using a SARS-CoV-2 NP ELISA and then infected (25 pg of NP per well) into Vero E6 TMPRSS2 cells for the plaque-forming assay described above.

### SIV infection

COS-1 cells were transfected with SIVmac239 (accession number M33262) molecular clone DNA (pBRmac239) and pTMPRSS2-Cherry vectors (in a 1:0.3 ratio) using FuGene 6. After 24 h of culture, the cells were washed and cultured for another 48 h in DMEM supplemented with 10% FBS. Culture supernatants were filtered with a 0.45-μm filter, and concentrations of p27 were measured by an SIV p27 Antigen Capture Assay kit (ABL, Rockville, MD, USA). Viruses (3 ng of p27 per well) were added to TZM-bl cells. After 72 h of culture, the luciferase activity in the infected cells was determined using a Bright-Glo luciferase assay system.

### HIV-1 infection

The molecular DNA clones of X4 tropic-EGFP-expressing HIV (pNL-E) and R5 tropic-DsRed-expressing HIV (pNLAD8-D) were kindly provided by Dr Yasuko Tsunetsugu-Yokota (Yamamoto et al, 2009). 293T cells were transfected with pNL-E or pNLAD8-D and pTMPRSS2-Cherry vectors (1:0.3 ratio in Fig. 2A; 400:125, 400:25, and 400:5 in Fig. 2C) using FuGene 6. After 24 h of culture, the cells were washed and cultured for another 48 h in DMEM supplemented with 10% FBS. Culture supernatants were filtered with a 0.45-μm filter, and concentrations of p24 were measured by an HIV Type 1 p24 Antigen ELISA 2.0 kit (ZeptoMetrix). Viruses (3 ng of p24 per well) were added to the human T-cell line, PM1-R5, and the cells were analyzed by flow cytometry after 72 h of culture.

### ELISA for viral proteins

Infectious viral particles were inactivated using RIPA lysis buffer, and the concentration of HIV-1 p24, SIV p27, and SARS-CoV-2 NP was determined by ELISA kits, HIV-1 p24 ELISA kit (ZeptoMetrix), SIV p27 Antigen Capture Assay kit (ABL), and SARS-CoV-2 NP ELISA (Proteintech), respectively.

### Single-pseudotyped virus imaging

293T cells were transfected with pCMV delta R8.2, pLentiLuc, and CoV-2-S-mCherry expression plasmid using FuGene 6. After 24 h, the cells were washed and cultured for another 48 h in DMEM containing 10% FBS. Culture supernatants were filtered through a 0.45-μm filter and bound onto an 8-well cover glass chamber coated with PLL for 12 h at 4 °C after washing with HBSS. Viral particles were visualized using a FLUOVIEW FV3000 with an OSR system (Evident).

### Flow cytometry analysis for CoV-2-S binding antibodies

293T cells were transfected with CoV-2-S and TM2-mCherry expression vectors using FuGene 6. After 24 h of culture, the cells were fixed with BD Cytofix/Cytoperm (554723, BD Biosciences, Franklin Lakes, NJ, USA) and stained with anti-SARS-CoV-2-S1 RBD (HL1003, 1:300 dilution) and anti-SARS-CoV-2-S2 (1A9, 1:300 dilution) antibodies in BD Perm/Wash (BD Biosciences) for 30 min at RT. The secondary antibodies used were anti-rabbit IgG-A488 (Abcam, 1:500) and anti-mouse IgG-A647 (Abcam, 1:500) in BD Perm/Wash for 30 min at RT. Cells were suspended in Mojo Sort buffer (BioLegend, San Diego, CA, USA) and analyzed using FACSCanto II and FlowJo (BD Biosciences).

### Immunoblotting analysis

293T cells were transfected with CoV-2-S, TM2, or pCMV delta R8.2 expression vectors or pNL4-3 using FuGene 6. After 48 h of culture, culture supernatants were filtered through a 0.45-μm filter, and viruses were collected by centrifugation on 20% of sucrose (Sigma) in PBS at 30,000 rpm for 90 min at 4 °C using an SW41 rotor (Beckman Coulter, Brea, CA, USA) and lysed using RIPA lysis buffer (16488-34, Nacalai Tesque, Kyoto, Japan). Cells were lysed in RIPA buffer containing a complete proteinase inhibitor (05892953001; Roche, Basel, Switzerland). SDS-PAGE was performed using the Mini-PROTEAN system (Bio-Rad, Hercules, CA, USA). After transfer to Immobilon-P PVDF membranes (IPVH00010, Millipore), immunoblotting analysis was performed with the following antibodies or antisera: anti-mCherry antibody (abcam, 1:1000 dilution), anti-SARS-CoV-2-S2 antibody (1A9, GeneTex, 1:1000 dilution), anti-SARS-CoV-2-S1 antibody (HL6, GeneTex, 1:1000 dilution), anti-HIV-1 p24 antibody (Nu24, 1:1000 dilution) (Terahara et al, 2011), anti-HIV gp120 antibody (ab21179, abcam, 1:1000 dilution) and anti-HIV-1 Env plasma pooled from HIV-1 positive individuals (1:1000 dilution). To analyze phosphorylated ERK, cell lysates were prepared in 25 mM Tris-Cl buffer (pH 8.0) including 150 mM NaCl, 1% NP-40, protease inhibitor (1187358001, Roche), and Phosphatase Inhibitor Cocktail (07575-51, Nacalai tesque), and anti-p-ERK (4370, Cell Signaling, Danvers, MA, USA, 1:2000 dilution), anti-ERK (686901, BioLegend, 1:1000 dilution), and anti-actin (M177-3, MBL, 1:10,000 dilution) antibodies were used for the immunoblotting analysis. Signals were detected using anti-mouse IgG-HRP (330, MBL, 1:10,000 dilution), anti-rabbit IgG-HRP (458, MBL, 1:10,000 dilution), anti-goat IgG-HRP (546, MBL, 1:10,000 dilution) or anti-human IgG-HRP (W4031, Promega, 1:10,000 dilution) antibodies with Immobilon HRP substrate (Millipore) using a ChemiDoc system (Bio-Rad) or FUSION-FX6 (Vilber Bio Imaging, Collegien, France).

### PLA

A total of 1 × 10^5^ transfected HEK 293 T cells were fixed, permeabilized with Cytofix/Cytoperm Solution (BD) for 20 min at 4 °C, and washed. PLA was performed using Duolink® flowPLA Detection Kit - FarRed (Sigma-Aldrich) according to the manufacturer’s protocol. Briefly, cells were incubated in Duolink Blocking Solution for 1 h at RT, pelleted, and incubated with anti-TM2 (abcam) and/or anti-SARS-CoV-2-S (HL1003, GeneTex) primary antibodies, diluted to 5 μg/mL in Duolink Antibody Diluent for 1 h at RT. Cells were washed, PLA probe anti-mouse PLUS and anti-rabbit MINUS diluted 1:5 in 10% FBS/Antibody Diluent were added, incubated for 1 h at 37 °C, and washed. Ligase diluted 1:40 in Duolink flowPLA Ligation Buffer was added and incubated for 30 min at 37 °C, followed by washing. Polymerase diluted 1:80 in Duolink flowPLA Amplification Buffer was added and incubated for 20 h at 37 °C. After washing, cells were incubated in Duolink flowPLA Detection Reagent FarRed for 30 min at 37 °C, washed, and resuspended in 0.8% PFA/PBS. The cells were then analyzed using flow cytometry (FACSCanto II, BD Bioscience).

### Proteome analysis

A total of 1 × 10^6^ 293T cells were transfected with TM2-mCherry or TM2-S441A-mCherry expression plasmids. After 24 h of culture, cells were washed with PBS and suspended in 50 μL of 8 M urea, 100 mM Tris-HCl (pH 8.5), 10 mM TCEP, and 40 mM CAA. Protein concentration was determined using a bicinchoninic acid (BCA) assay (Thermo Fisher Scientific). The protein solution was diluted fivefold with 50 mM ammonium bicarbonate (FUJIFILM Wako), and proteins were digested overnight with LysC (FUJIFILM Wako) and trypsin (sequence grade; Promega) at a protein:enzyme ratio of 100:1 for each enzyme at 25 °C.

LC-MS/MS analysis was performed on an Orbitrap Eclipse mass spectrometer (Thermo Fisher Scientific), equipped with a FAIMSpro interface and combined with a Vanquish Neo UHPLC pump (Thermo Fisher Scientific). The mobile phases consisted of (A) 0.1% formic acid and (B) 0.1% formic acid and 80% acetonitrile (ACN). Peptides were loaded on a self-made 18-cm fused-silica emitter (100-µm inner diameter), packed with ReproSil-Pur C18-AQ (1.9 µm, Dr. Maisch, Ammerbuch, Germany) and separated by a linear gradient for 60 min (5–40% B over 45 min, 40–99% B over 5 min, and 99% B for 10 min) at a flow rate of 350 nL/min. The FAIMS compensation voltages (CVs) were fixed at 45. MS scanning was performed in data-independent acquisition (DIA) mode using an Orbitrap analyzer. MS1 scans were performed in the range of 350–1000 *m/z* (resolution = 120,000, maximum injection time = 45 ms, and automatic gain control = 300%). In the subsequent MS/MS scans, the precursor range was set to 500–740 *m/z*, and 60 scans were acquired with an isolation window of 4 *m/z* and an HCD-normalized collision energy of 27 (resolution = 15,000, injection time = 22 ms, auto gain control = 1000%, first mass = 120 *m/z*).

Raw files were processed using DIA-NN (v.1.8.1) (PMID 31768060) to perform a library-free search against the UniProt/SwissProt human database combined with the contaminant database obtained from MaxQuant (PMID 27809316) using the following parameters: up to two missed cleavages, precursor charge state of 2–4, precursor range of 500–740 *m/z*, and fragment ion range of 120–1800 *m/z*. The maximum number of variable modifications was set to three. Variable protein N-terminal methionine excision, methionine oxidation, N-terminal acetylation, and fixed cysteine carbamidomethylation were considered. Matches between the runs were performed. Ingenuity Pathway Analysis (Qiagen IPA, Qiagen, Venlo, Netherlands) was performed for 704 proteins that were statistically different (*P* < 0.05) in three independent analyses of TM2-WT- and TM2-S441A-transfected cells, respectively.

### Nafamostat removal assay

293T cells were transfected with Golgi-YFP and TM2 expression plasmids using FuGene 6 and were cultured in DMEM including 10% FBS and 2–200 μM nafamostat on an 8-well chambered cover glass, coated with PLL. At 24 h post-transfection, the medium was replaced with fresh DMEM containing 10% FBS with or without kinase inhibitors (ERK inhibitor, 15944, Cayman Chemical, Ann Arbor, MI, USA; CDK1 inhibitor Ro 3306, ab141491, CDK5 inhibitor 20-233, HY-123772, MCE, Monmouth Junction, NJ, USA) and cultured for 30–180 min before fixation with 2% formaldehyde. Immunostaining and imaging analyses were performed as previously described.

### Structural analysis of CoV-2-S for the 1A9 epitope

Structural analysis was performed using the UCSF Chimera software (Pettersen et al, 2004). The epitope amino acid sequence (VIGIVNNTVYDPLQPELDSF) of the 1A9 antibody located in the closed (PDB:6VXX) (Walls et al, 2020) and open (PDB:8FDW) (Shi et al, 2023) form of the CoV-2-S structure is highlighted in blue (Fig. 3G).

### CoV-2-E inhibitor assay

293T cells were transfected with Golgi-YFP, CoV-2-E, and TM2 expression plasmids using FuGene 6 and were cultured in DMEM including 10% FBS and 3 μM CoV-2-E inhibitory peptide, iPep-SARS-E (Bekdash et al, 2024), and control peptide (NH_2_-GRKKRRQRRRPPQGSGSGKPIPNPLLGLDST-COOH) on an 8-well chambered cover glass coated with PLL. At 24 h post-transfection, immunostaining and imaging analyses were performed as previously described.

### Subcellular fractionation

293T cells were transfected with CoV-2-S and TM2-WT-Cherry or TM2-S441A-Cherry expression plasmids using FuGene 6. After 24 h of culture, cells were suspended in 10 mM Tris-Cl (pH 7.5) buffer including 1 mM EDTA (Sigma) and 0.25 M Sucrose (Sigma) and homogenized with Dounce homogenizer. Homogenates were placed on the 4% - 34% step gradient of OptiPrep (Sigma) including 10 mM Tris-Cl (pH 7.5), 1 mM EDTA and 0.25 M Sucrose, and centrifuged at 55,000 rpm for 16 hr at 4 °C using an SW60Ti rotor (Beckman). A 250 μl of each fraction was collected from top and was lysed in Sample buffer. SDS-PAGE was performed using the Mini-PROTEAN system. After transfer to PVDF membranes using Trans-Blot Turbo system, immunoblotting analysis was performed with the following antibodies: anti-SARS-CoV-2-S2 antibody (GT745, GeneTex, 1:10,000 dilution), anti-Calnexin antibody (2433, Cell Signaling, 1:1000 dilution), anti-Gorasp2 antibody (10598-1-AP, Proteintech, 1:4000 dilution), and anti-ATP1P antibody (604752, Biolegend, 1:1000 dilution).

### TMPRSS2 knockdown in Caco-2 cells

293T cells were transfected with pCMV delta R8.2, VSV-G plasmids, and the lentiviral GFP vectors pool, including human TMPRSS2 targeting shRNA sequences (TL308745, ORIGENE, Rockville, MD, USA) using FuGene 6. After 6 h, the cells were washed and cultured for another 24 h in DMEM containing 10% FBS. Culture supernatants were filtered through a 0.45-μm filter and were inoculated into Caco-2 cells. The transfected Caco-2 cells were selected by 3 μg/ml puromycin for 3 weeks.

### Quantitative PCR

Total RNA was extracted from cells using TRIzol (Thermo Fisher Scientific) and Direct-zol RNA Kit (Zymo Research, Irvine, CA, USA). TaqMan PCR was carried out with QuantiTect RT-PCR Kit (Qiagen) and primers listed in the Reagents and Tools Table using CFX Maestro Ver. 2.3 (Bio-Rad). For the standard use, the double-stranded DNA, including target sequences, was synthesized by GENEWIZ (Tokyo, Japan).

### Biosafety

Experiments using SARS-CoV-2 were conducted at the BSL-3 facility at RIKEN Yokohama with the approval of the RIKEN Yokohama Microbial Safety Committee. Experiments using HIV-1 and SIV were conducted at the BSL-3 facility at JIHS-NIID with the approval of the NIID Biorisk Management Committee.

### Statistics

The Prism 8.4.3 software (GraphPad, San Diego, CA, USA) was used for statistical analysis.

## Supplementary information

Peer Review File

Source data Fig. 1

Source data Fig. 2

Source data Fig. 3

Source data Fig. 4

Source data Fig. 5

Source data Fig. 6

Source data Fig. 7

Source data Fig. 8

Expanded View Figures

## Data Availability

The proteomics data are deposited in the ProteomeXchange Consortium via the jPOST partner repository (https://repository.jpostdb.org/entry/JPST003877.1). The source data of this paper are collected in the following database record: biostudies:S-SCDT-10_1038-S44319-026-00797-2.
