# Supplementary figures and images for: TMPRSS2-induced Golgi disruption restricts the incorporation of virus envelope glycoproteins into virions
Source: EMBO Rep. 2026 May 19;27(12):3424–53. doi: 10.1038/s44319-026-00797-2 (PMC13303877; doi:10.1038/s44319-026-00797-2)

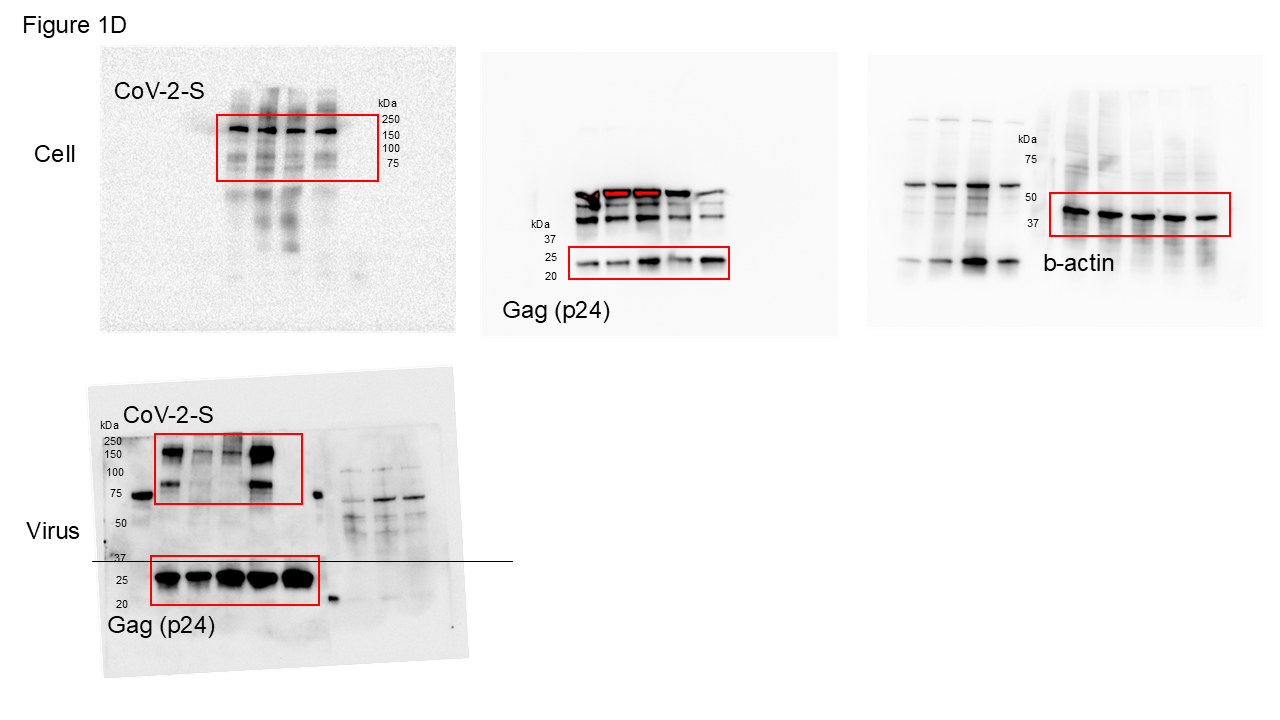

Supplement: Supplementary file 2 — Source data Fig. 1 [file 44319_2026_797_MOESM2_ESM.zip › Figure 1/CoV2S_p24_Actin_WB.tif]

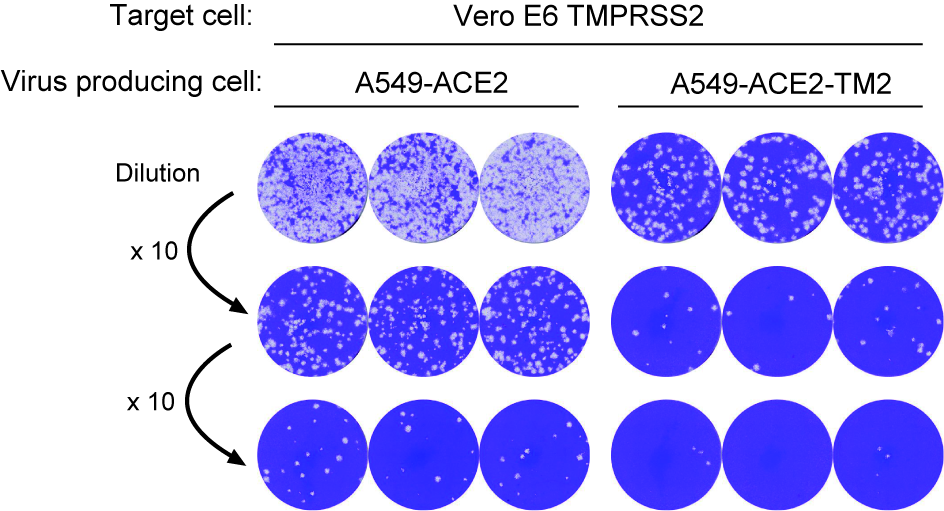

Supplement: Supplementary file 2 — Source data Fig. 1 [file 44319_2026_797_MOESM2_ESM.zip › Figure 1/Fig1B.tif]

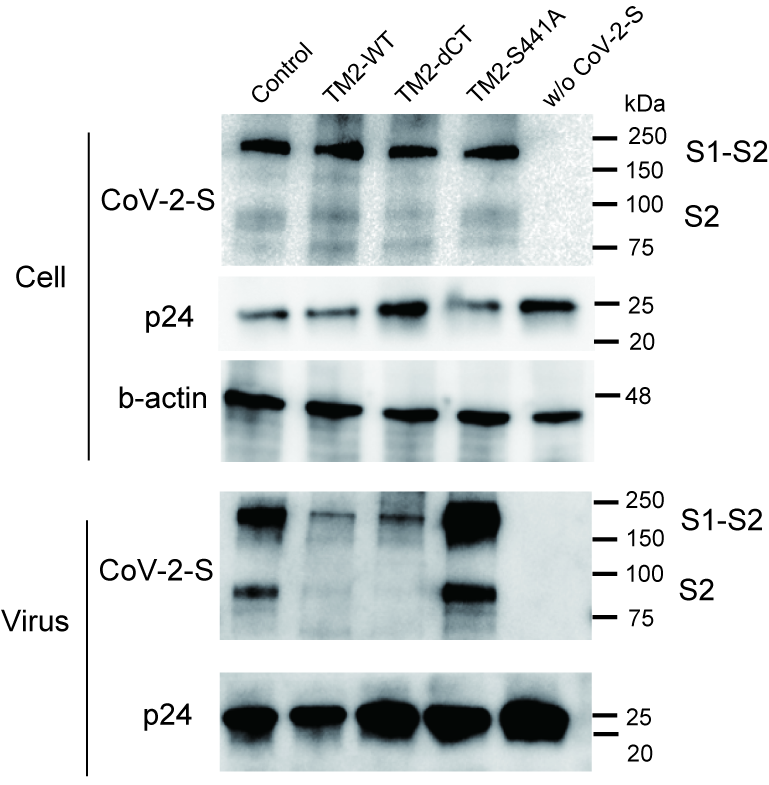

Supplement: Supplementary file 2 — Source data Fig. 1 [file 44319_2026_797_MOESM2_ESM.zip › Figure 1/Fig1D.tif]

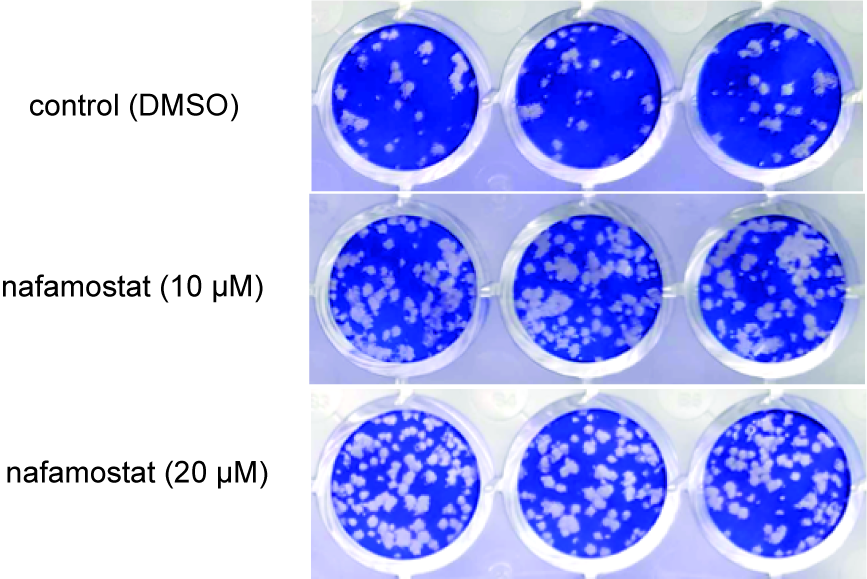

Supplement: Supplementary file 2 — Source data Fig. 1 [file 44319_2026_797_MOESM2_ESM.zip › Figure 1/Fig1E.tif]

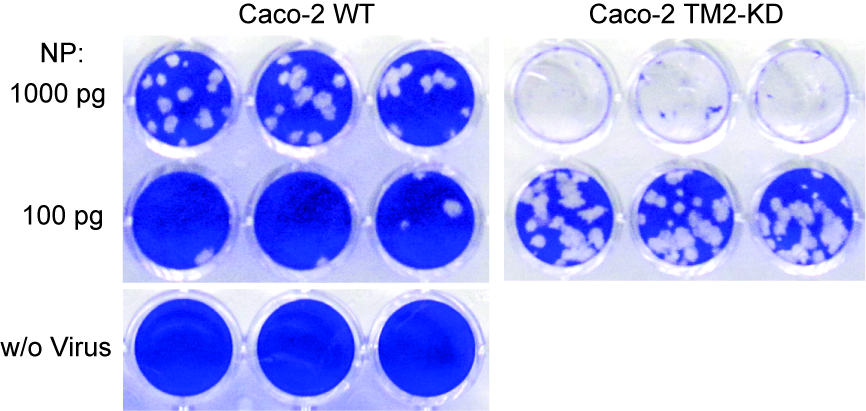

Supplement: Supplementary file 2 — Source data Fig. 1 [file 44319_2026_797_MOESM2_ESM.zip › Figure 1/Fig1F.tif]

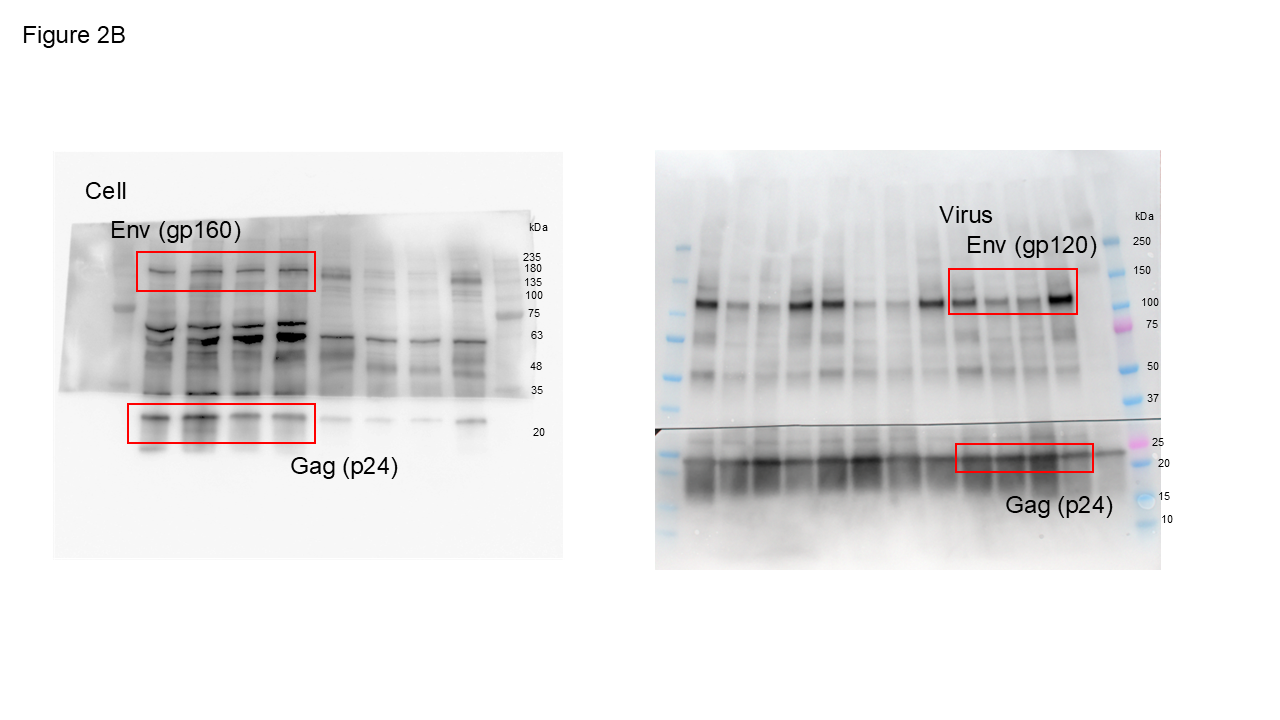

Supplement: Supplementary file 3 — Source data Fig. 2 [file 44319_2026_797_MOESM3_ESM.zip › Figure 2/Env_Gag_WB.tif]

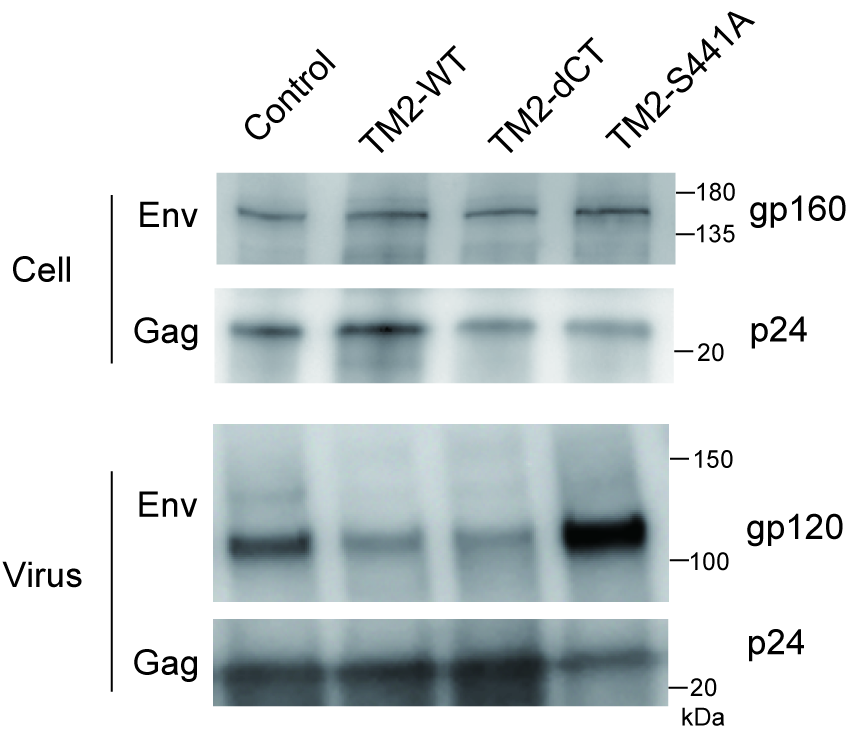

Supplement: Supplementary file 3 — Source data Fig. 2 [file 44319_2026_797_MOESM3_ESM.zip › Figure 2/Fig2B.tif]

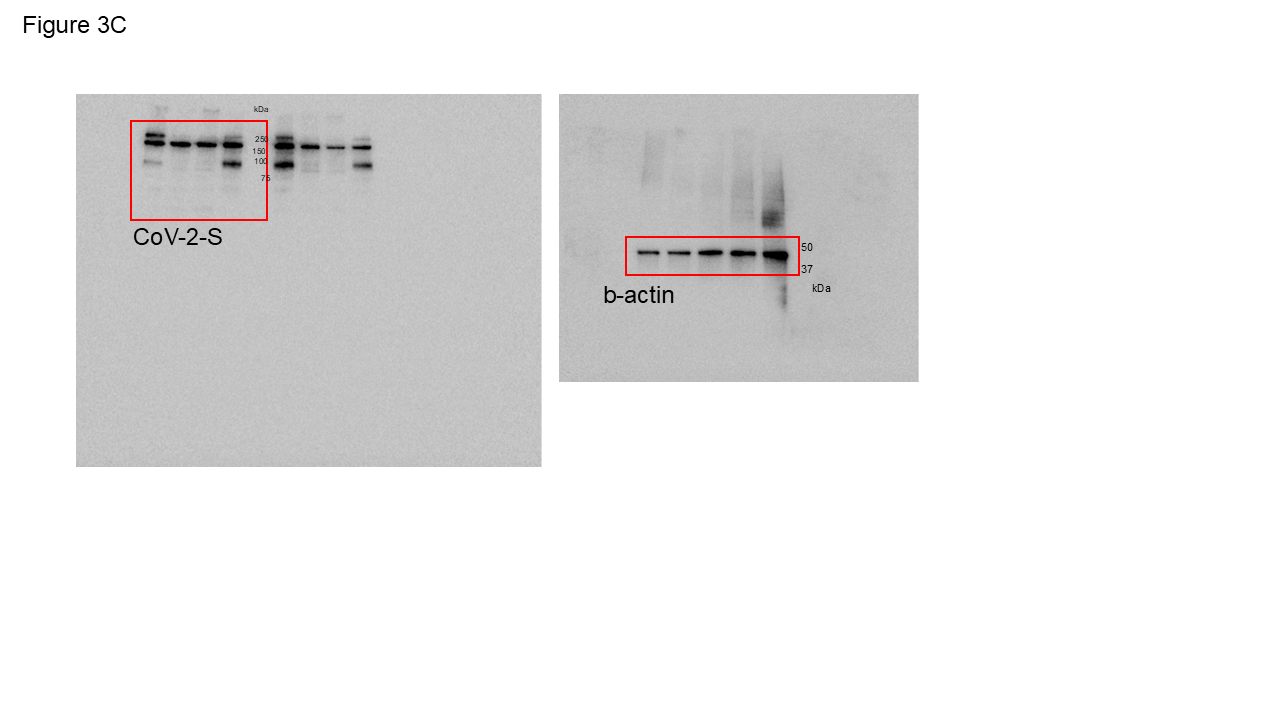

Supplement: Supplementary file 4 — Source data Fig. 3 [file 44319_2026_797_MOESM4_ESM.zip › Figure 3/CoV2S_actin_WB.tif]

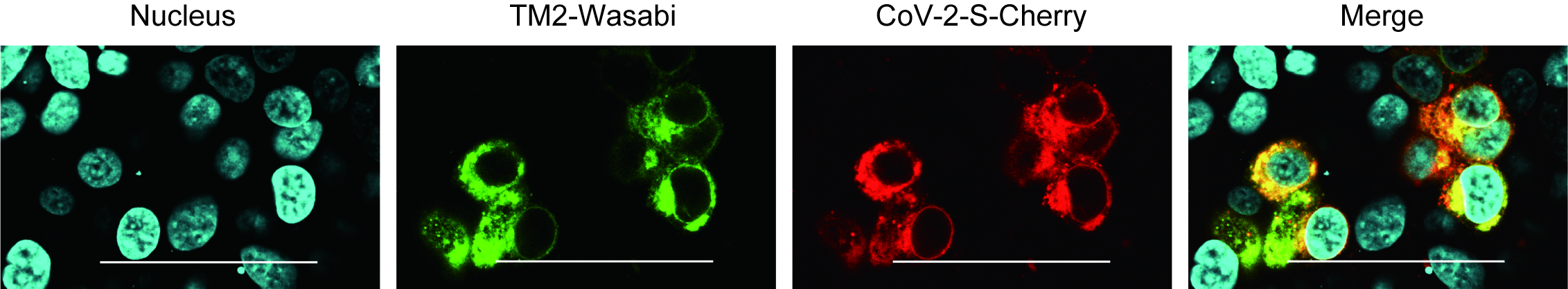

Supplement: Supplementary file 4 — Source data Fig. 3 [file 44319_2026_797_MOESM4_ESM.zip › Figure 3/Fig3A.tif]

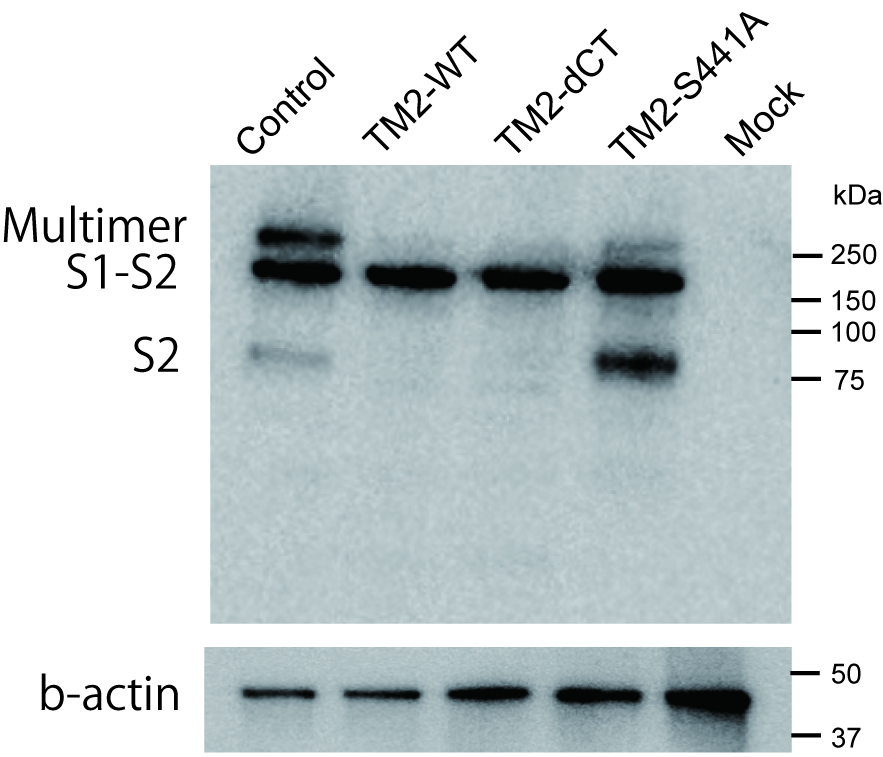

Supplement: Supplementary file 4 — Source data Fig. 3 [file 44319_2026_797_MOESM4_ESM.zip › Figure 3/Fig3C.tif]

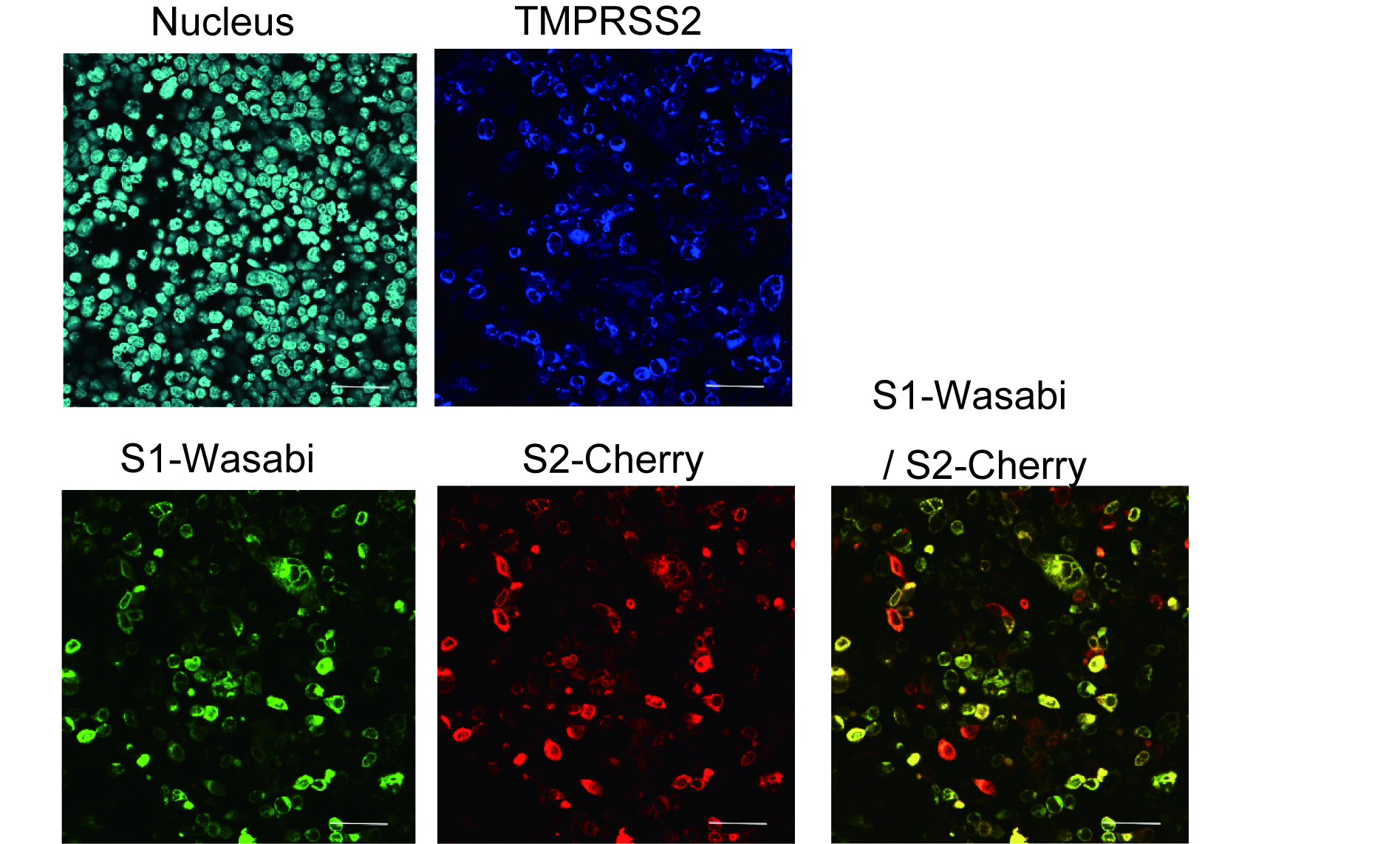

Supplement: Supplementary file 4 — Source data Fig. 3 [file 44319_2026_797_MOESM4_ESM.zip › Figure 3/Fig3D.tif]

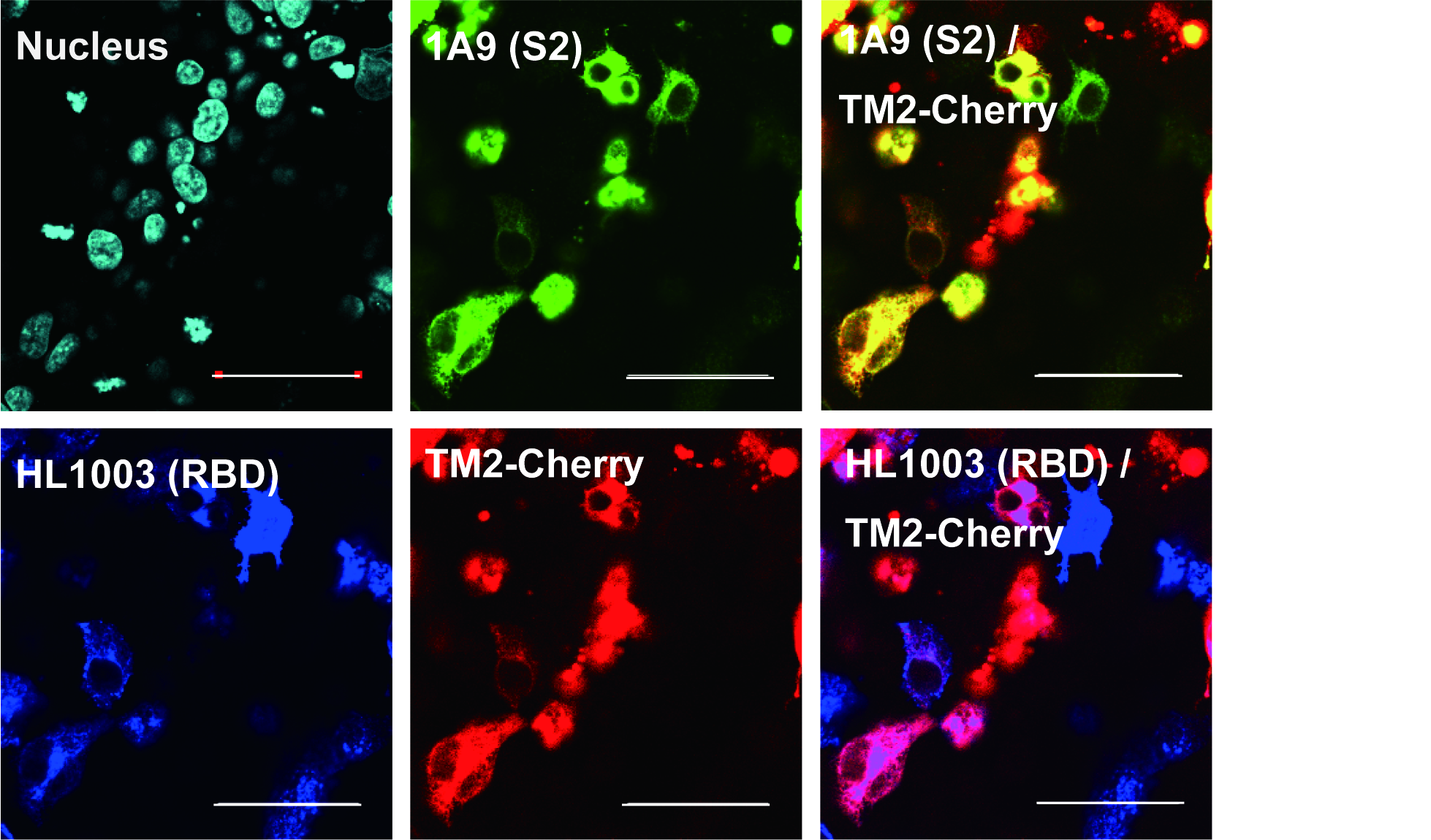

Supplement: Supplementary file 4 — Source data Fig. 3 [file 44319_2026_797_MOESM4_ESM.zip › Figure 3/Fig3E.tif]

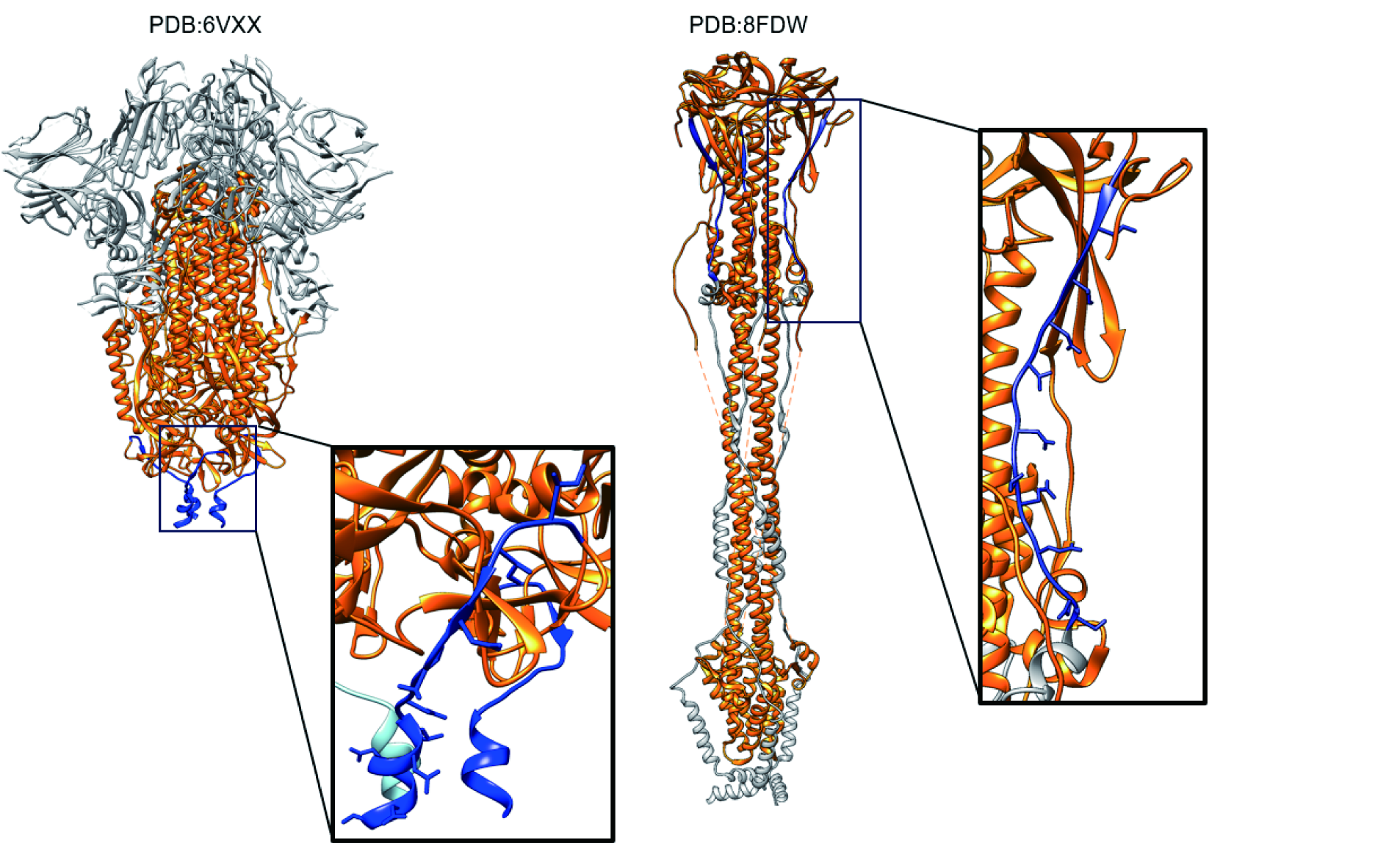

Supplement: Supplementary file 4 — Source data Fig. 3 [file 44319_2026_797_MOESM4_ESM.zip › Figure 3/Fig3G.tif]

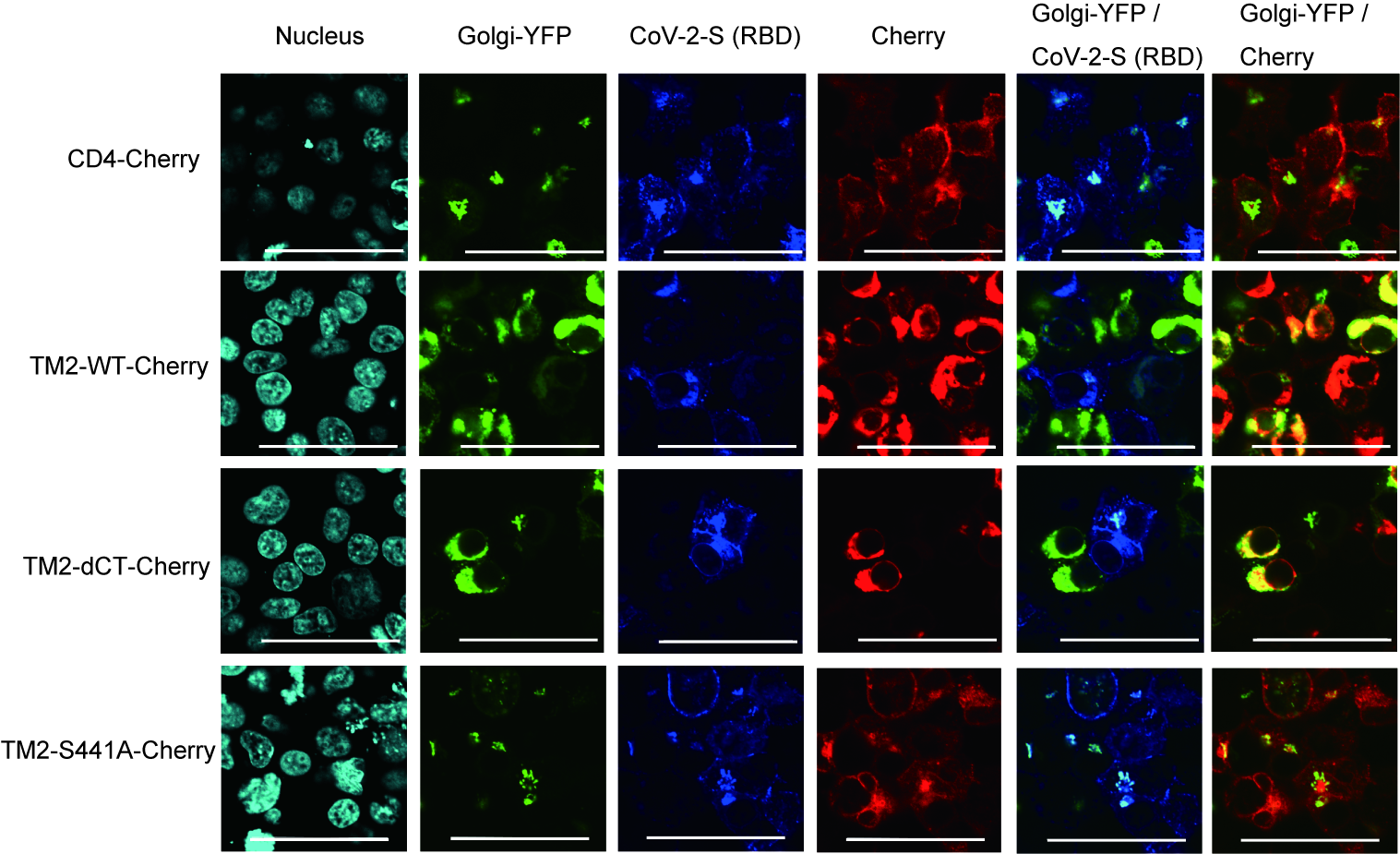

Supplement: Supplementary file 5 — Source data Fig. 4 [file 44319_2026_797_MOESM5_ESM.zip › Figure 4/Fig4A.tif]

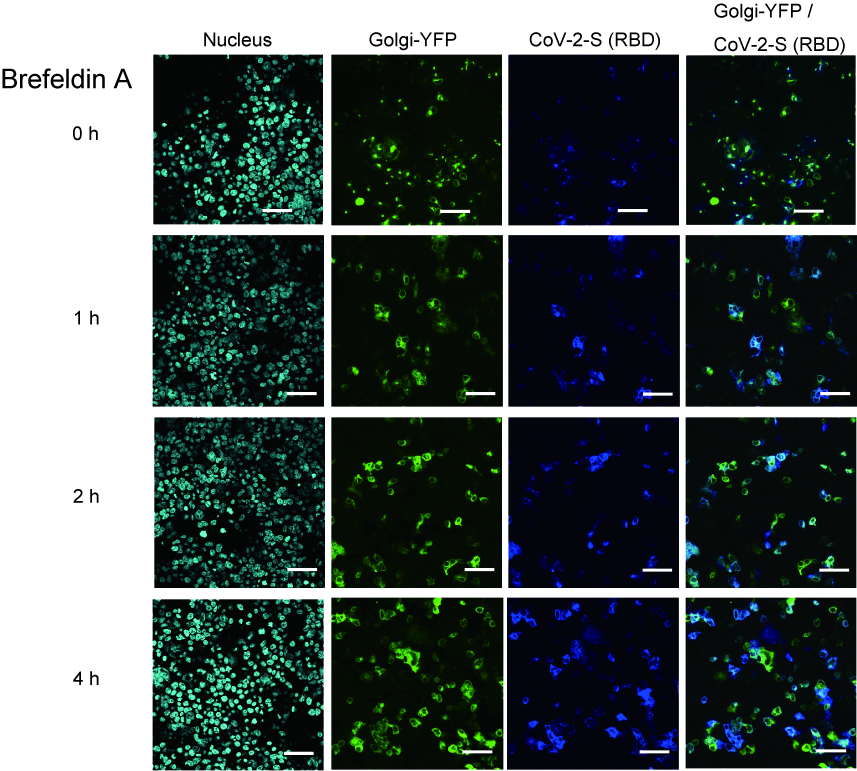

Supplement: Supplementary file 5 — Source data Fig. 4 [file 44319_2026_797_MOESM5_ESM.zip › Figure 4/Fig4B.tif]

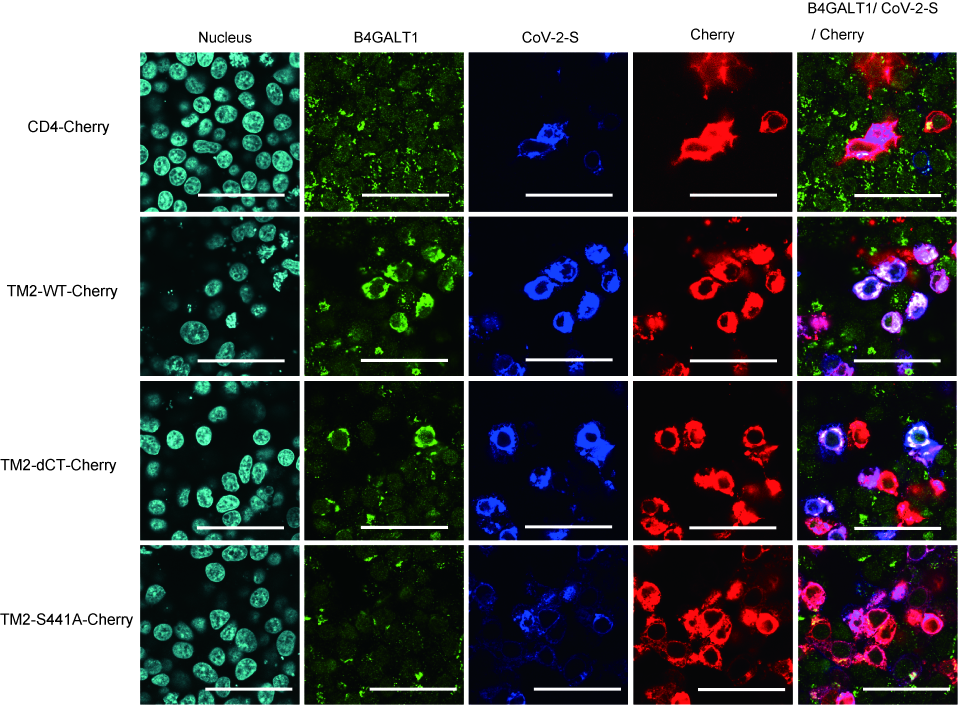

Supplement: Supplementary file 5 — Source data Fig. 4 [file 44319_2026_797_MOESM5_ESM.zip › Figure 4/Fig4C.tif]

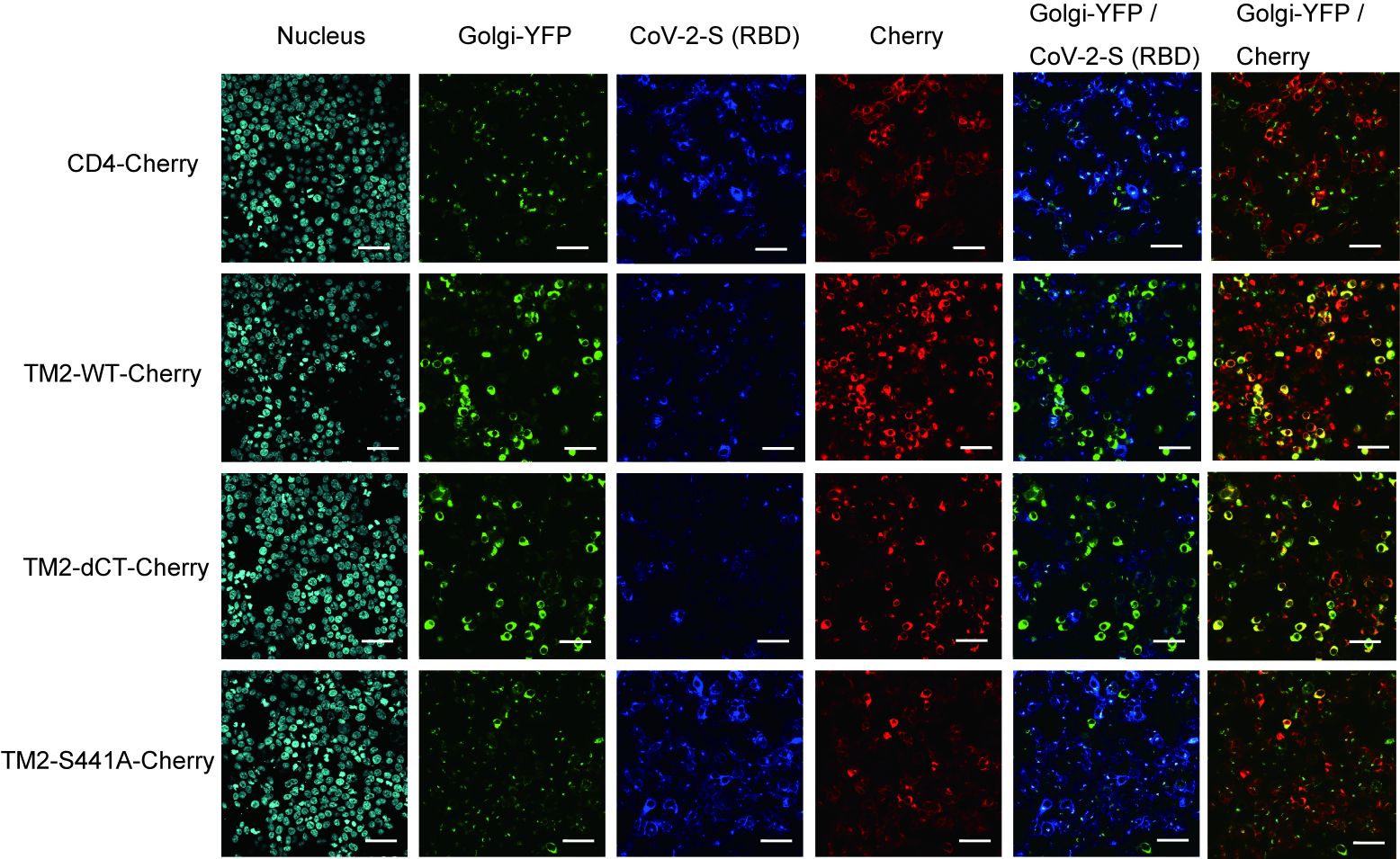

Supplement: Supplementary file 5 — Source data Fig. 4 [file 44319_2026_797_MOESM5_ESM.zip › Figure 4/Fig4D.tif]

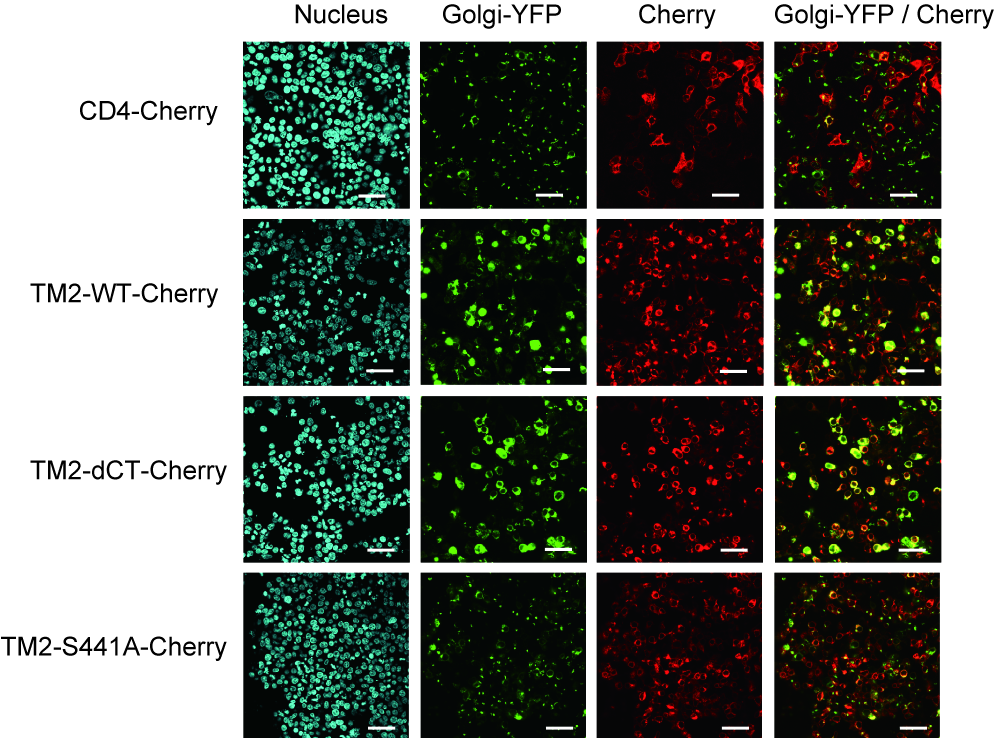

Supplement: Supplementary file 6 — Source data Fig. 5 [file 44319_2026_797_MOESM6_ESM.zip › Figure 5/Fig5A.tif]

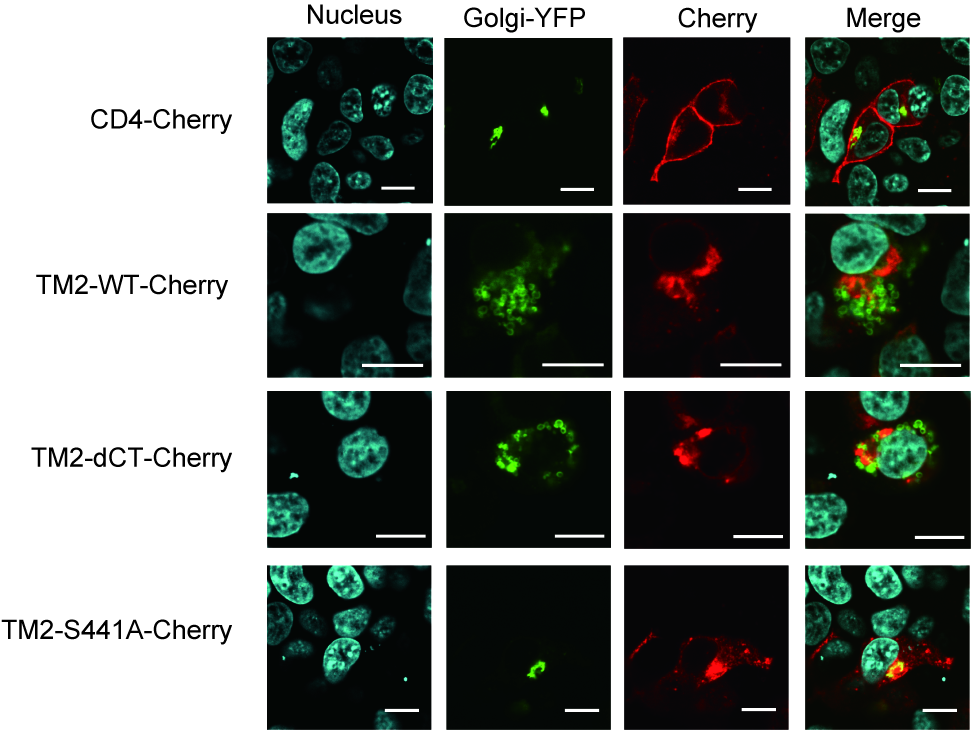

Supplement: Supplementary file 6 — Source data Fig. 5 [file 44319_2026_797_MOESM6_ESM.zip › Figure 5/Fig5C.tif]

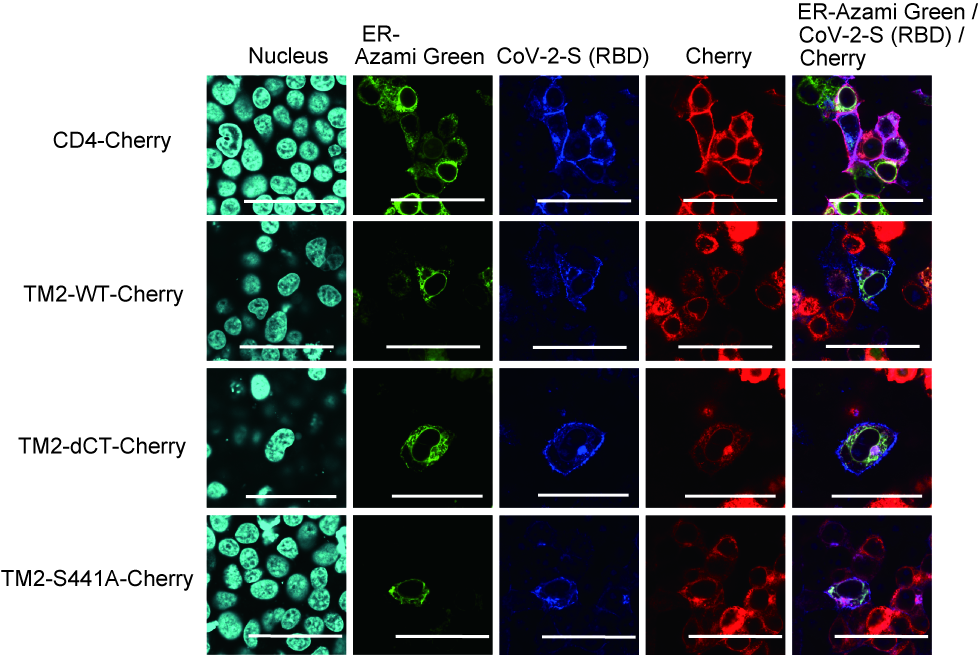

Supplement: Supplementary file 6 — Source data Fig. 5 [file 44319_2026_797_MOESM6_ESM.zip › Figure 5/Fig5D.tif]

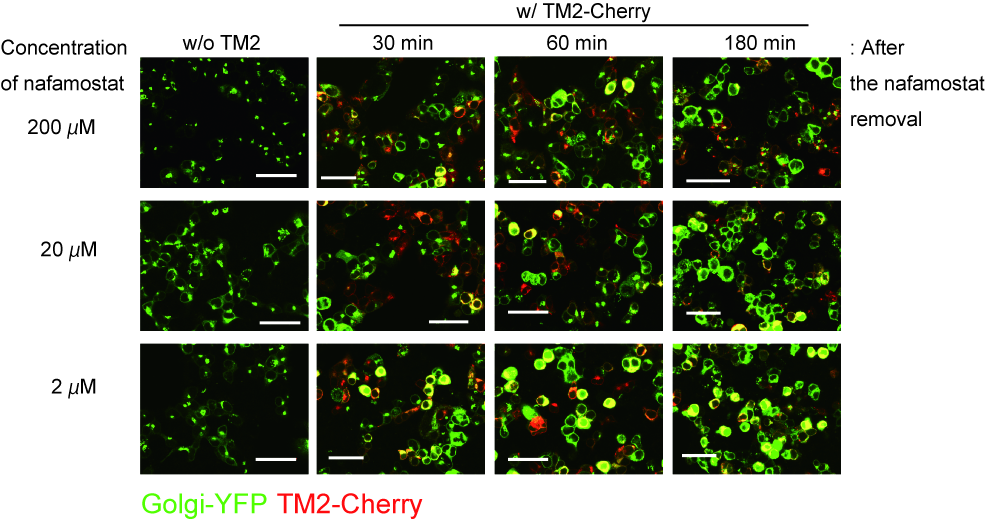

Supplement: Supplementary file 6 — Source data Fig. 5 [file 44319_2026_797_MOESM6_ESM.zip › Figure 5/Fig5F.tif]

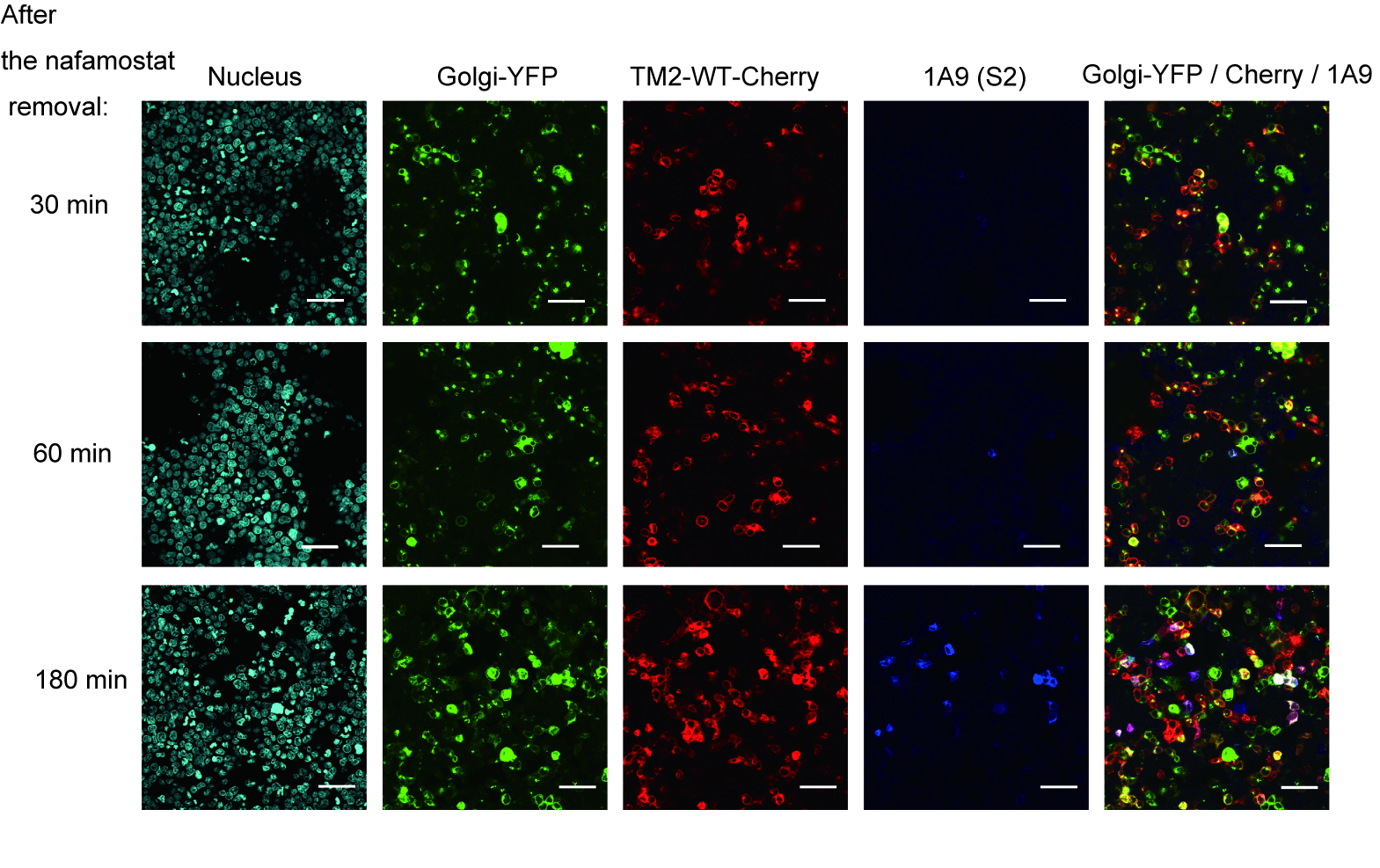

Supplement: Supplementary file 6 — Source data Fig. 5 [file 44319_2026_797_MOESM6_ESM.zip › Figure 5/Fig5H.tif]

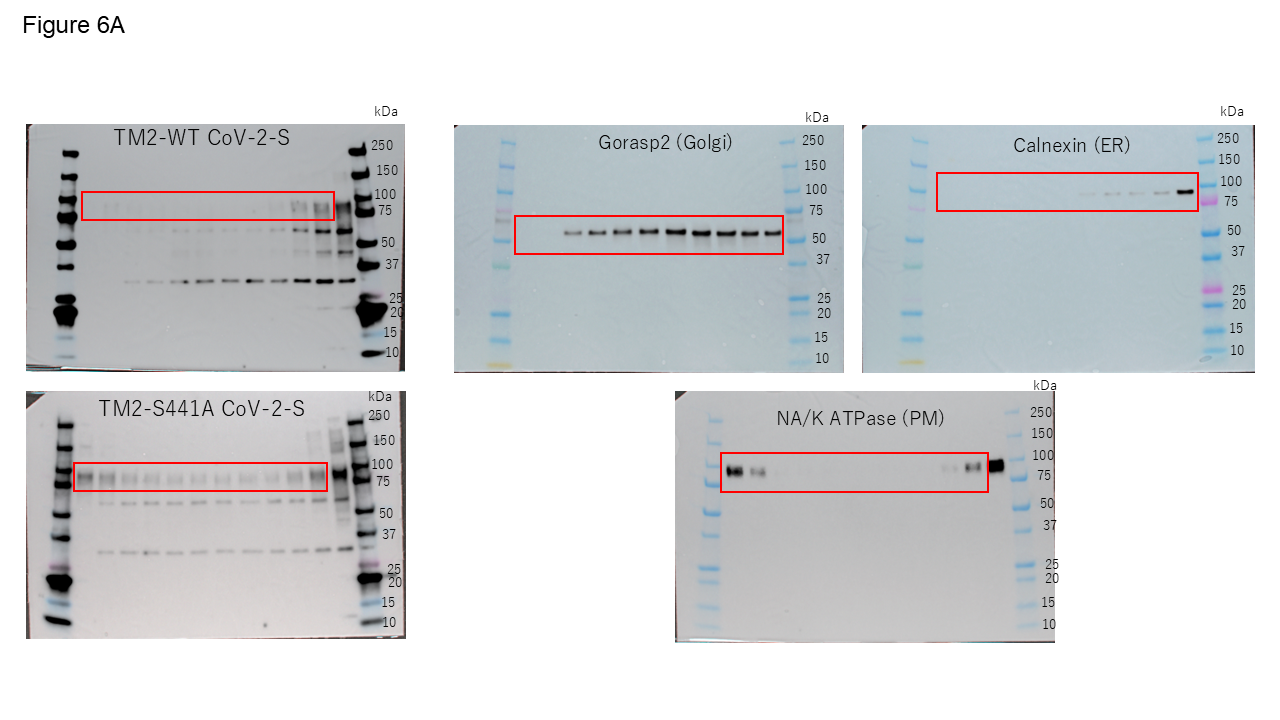

Supplement: Supplementary file 7 — Source data Fig. 6 [file 44319_2026_797_MOESM7_ESM.zip › Figure 6/CoV2S_ER_Golgi_PM_WB.tif]

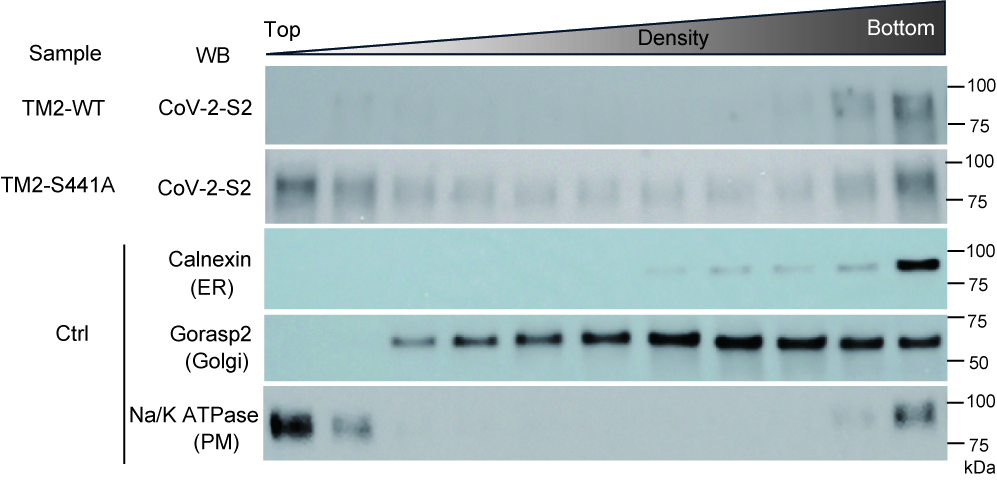

Supplement: Supplementary file 7 — Source data Fig. 6 [file 44319_2026_797_MOESM7_ESM.zip › Figure 6/Fig6A.tif]

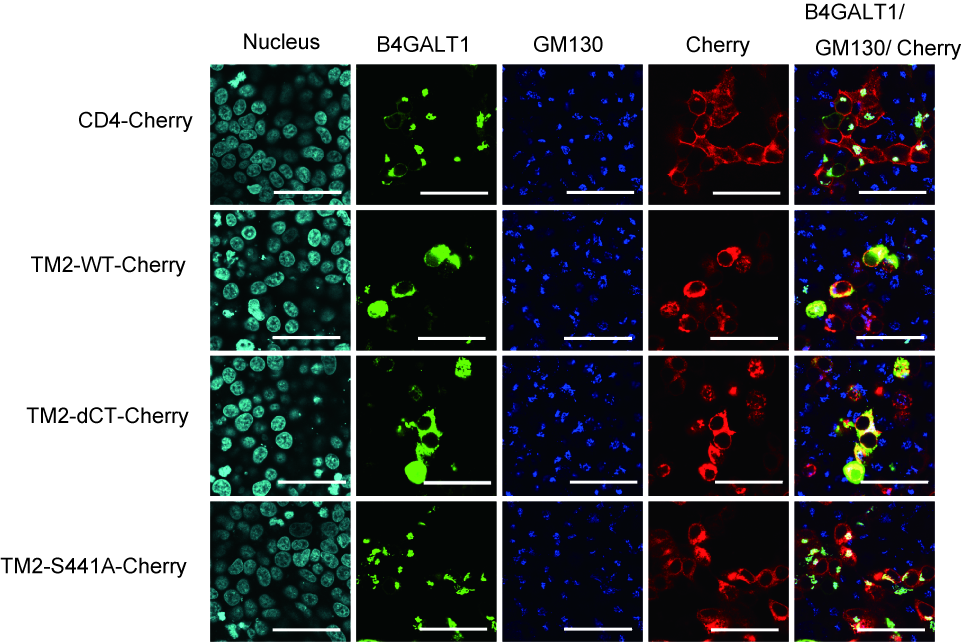

Supplement: Supplementary file 7 — Source data Fig. 6 [file 44319_2026_797_MOESM7_ESM.zip › Figure 6/Fig6B.tif]

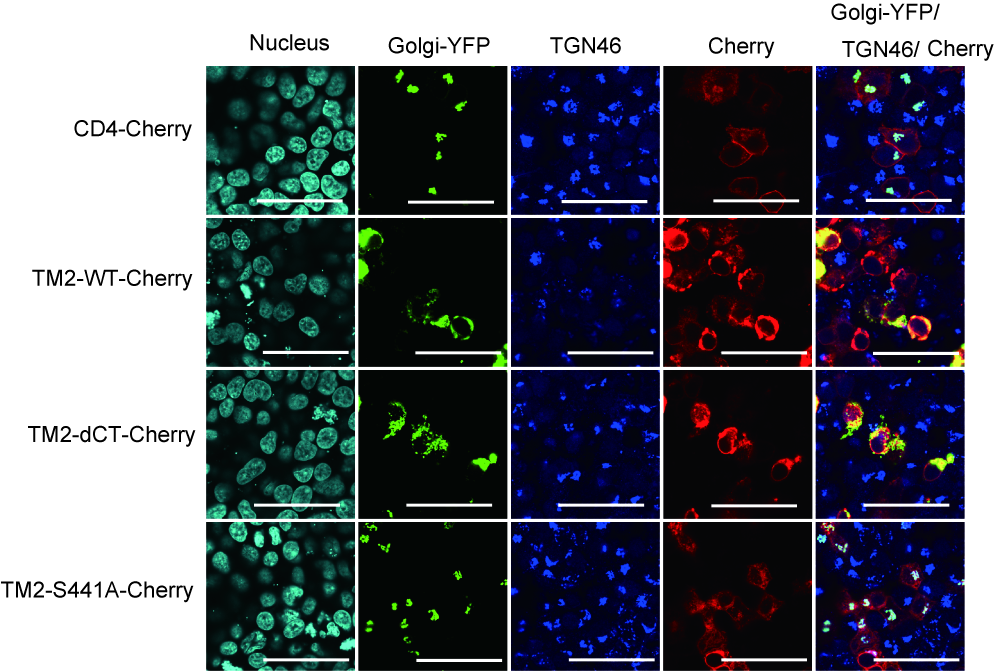

Supplement: Supplementary file 7 — Source data Fig. 6 [file 44319_2026_797_MOESM7_ESM.zip › Figure 6/Fig6C.tif]

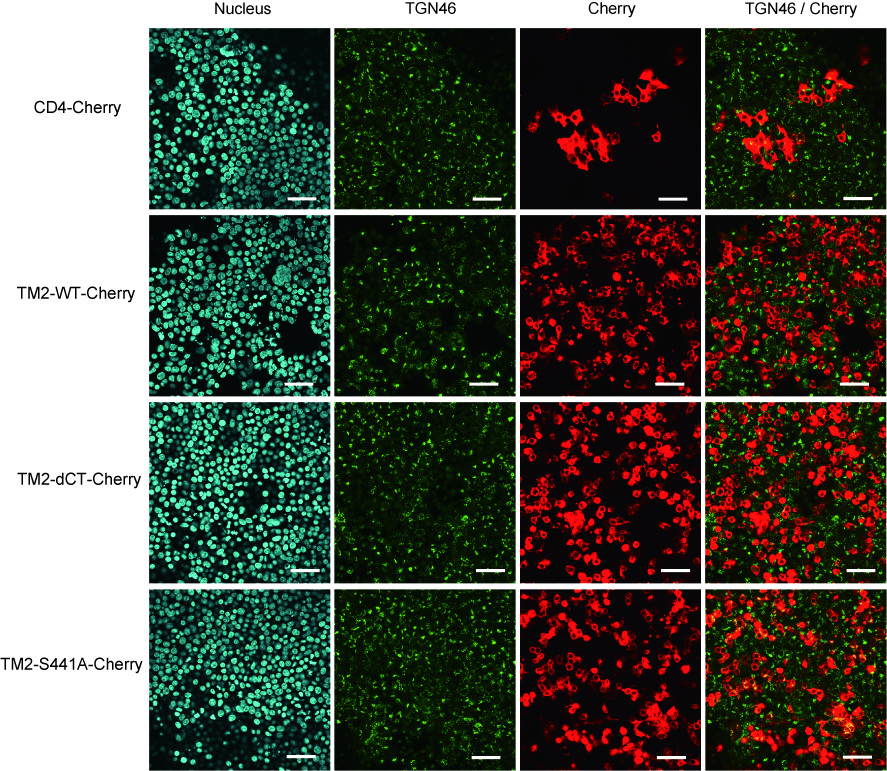

Supplement: Supplementary file 7 — Source data Fig. 6 [file 44319_2026_797_MOESM7_ESM.zip › Figure 6/Fig6D.tif]

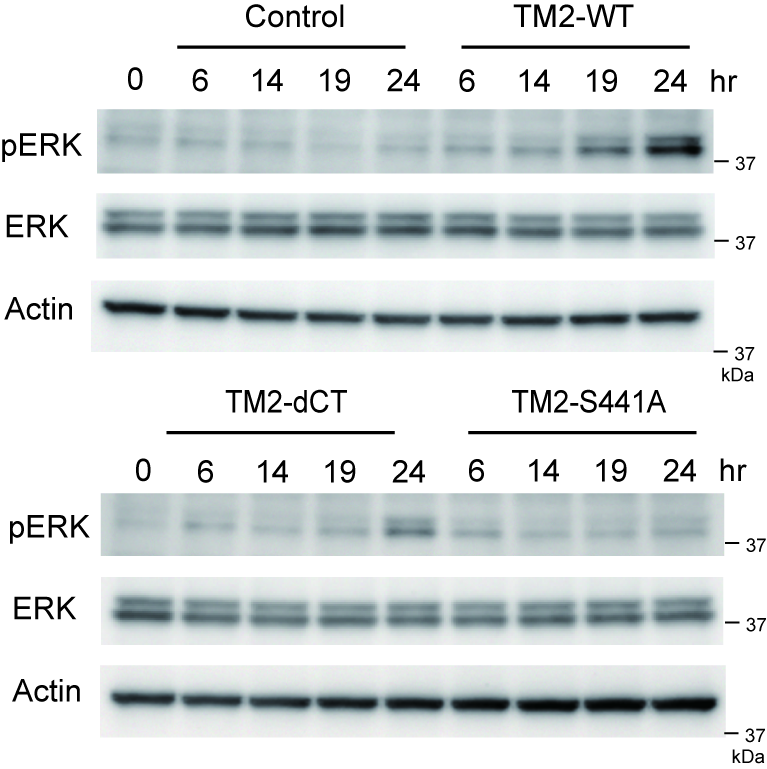

Supplement: Supplementary file 8 — Source data Fig. 7 [file 44319_2026_797_MOESM8_ESM.zip › Figure 7/Fig7A.tif]

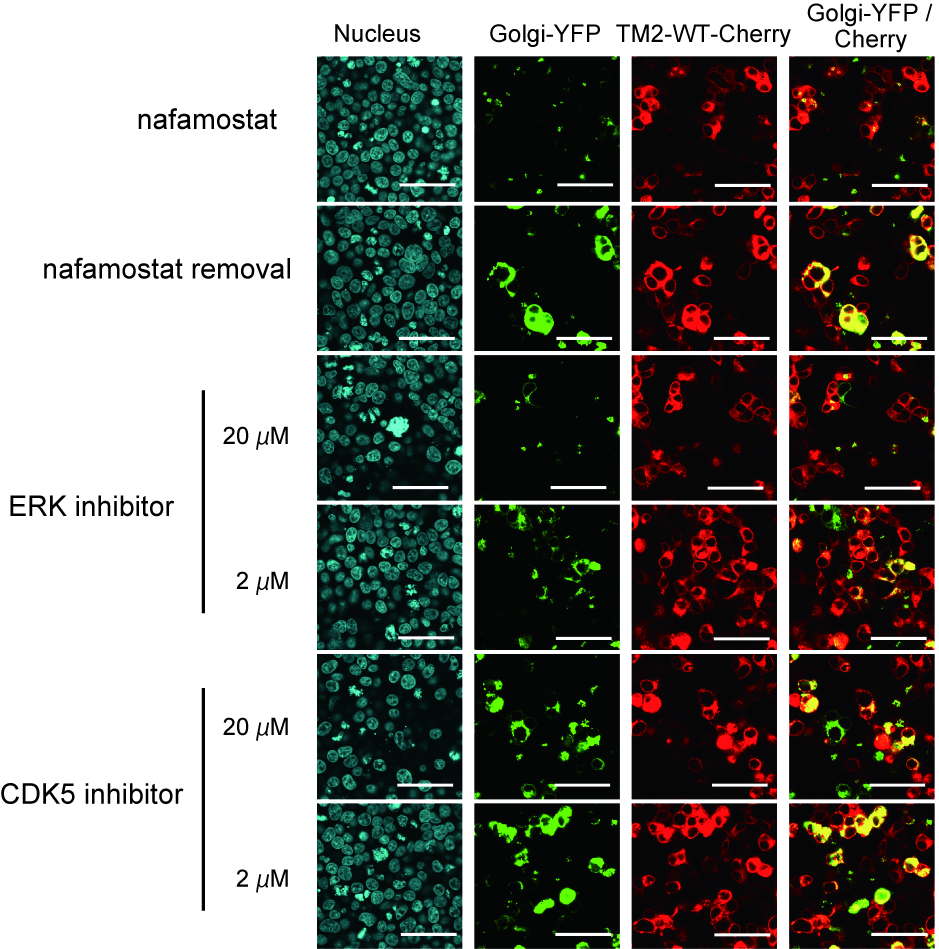

Supplement: Supplementary file 8 — Source data Fig. 7 [file 44319_2026_797_MOESM8_ESM.zip › Figure 7/Fig7D.tif]

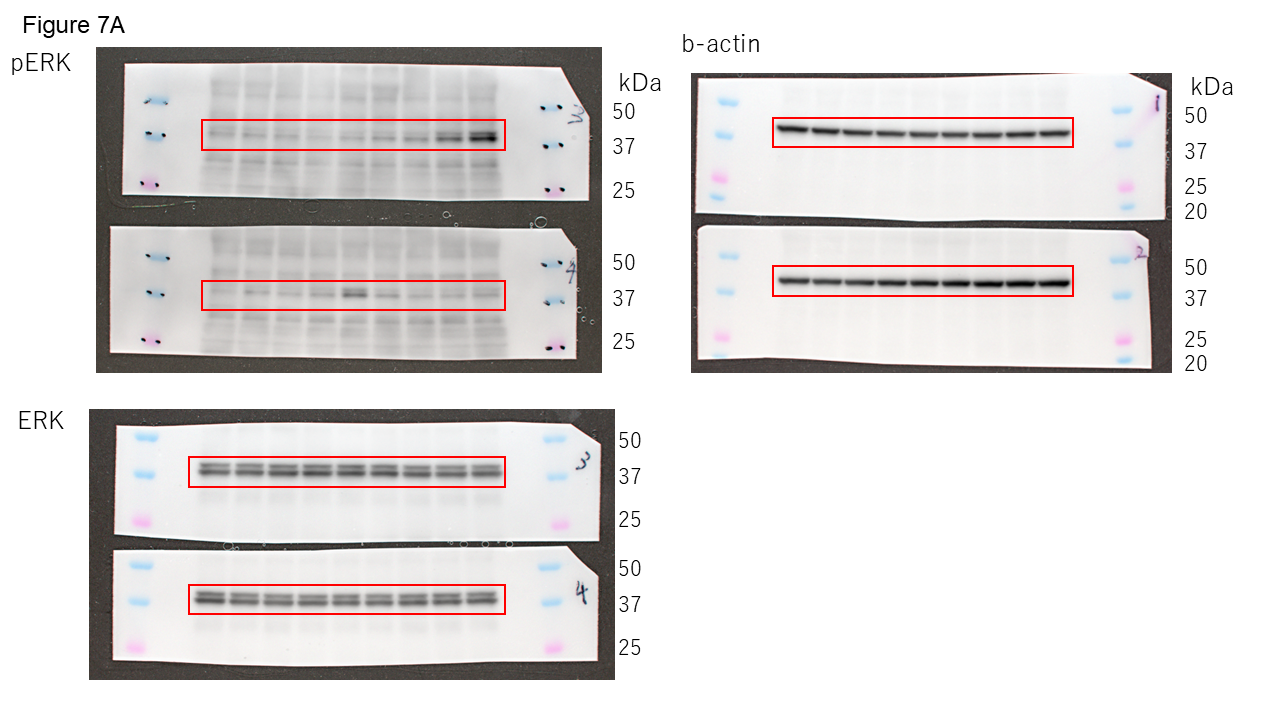

Supplement: Supplementary file 8 — Source data Fig. 7 [file 44319_2026_797_MOESM8_ESM.zip › Figure 7/pERK_ERK_actin_WB.tif]

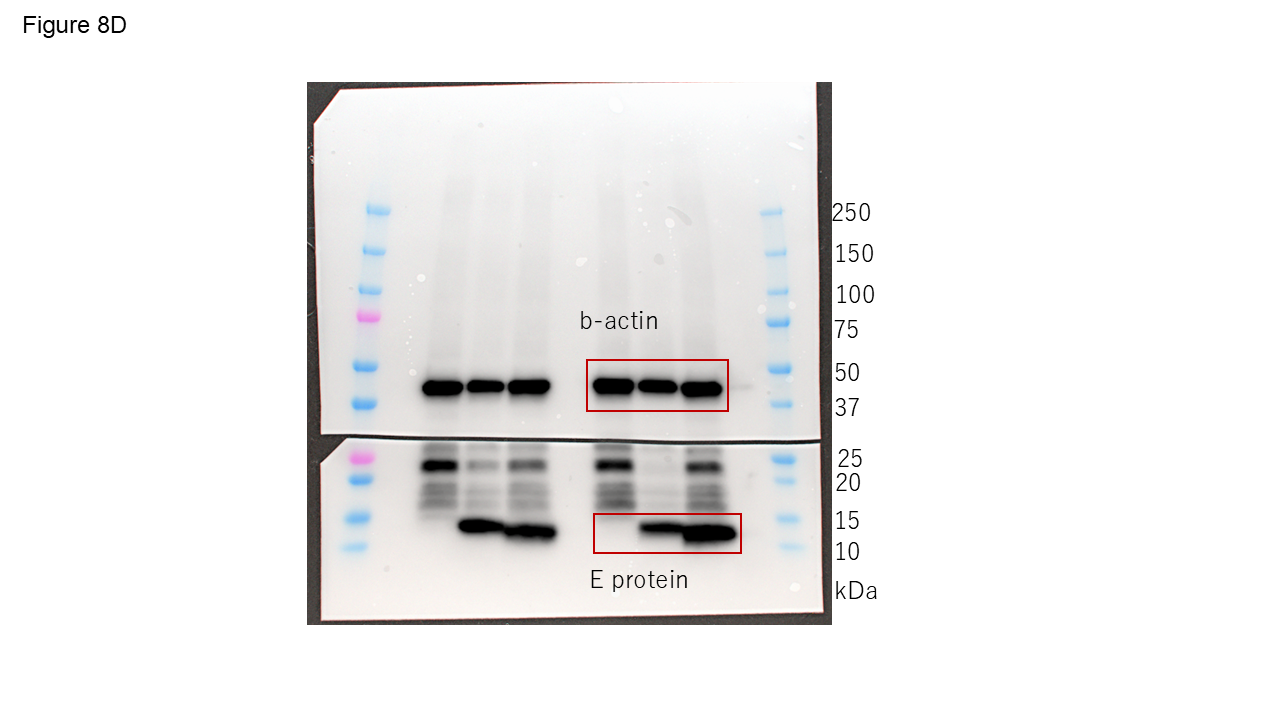

Supplement: Supplementary file 9 — Source data Fig. 8 [file 44319_2026_797_MOESM9_ESM.zip › Figure 8/CoV2E_actin_WB.tif]

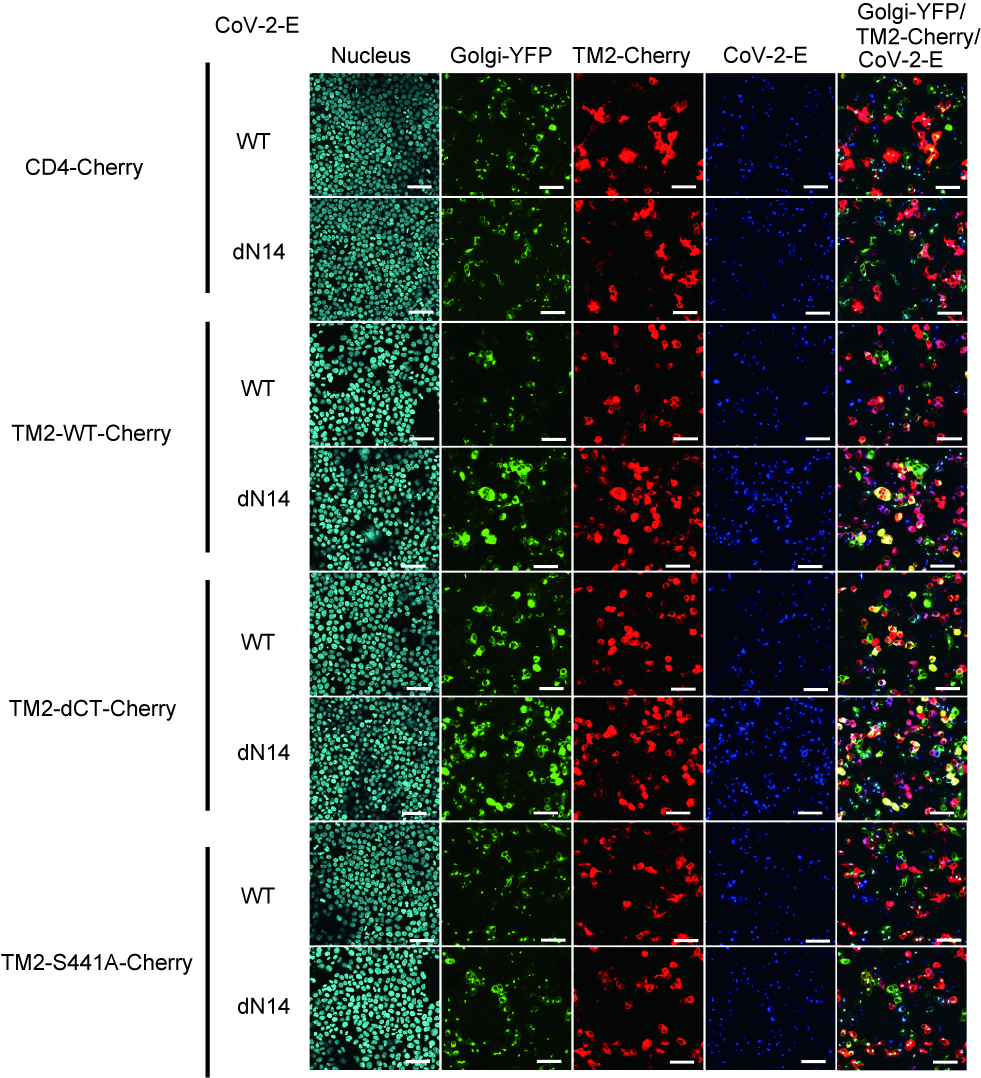

Supplement: Supplementary file 9 — Source data Fig. 8 [file 44319_2026_797_MOESM9_ESM.zip › Figure 8/Fig8A.tif]

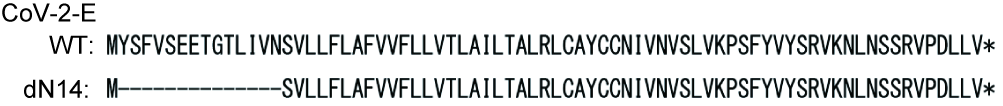

Supplement: Supplementary file 9 — Source data Fig. 8 [file 44319_2026_797_MOESM9_ESM.zip › Figure 8/Fig8C.tif]

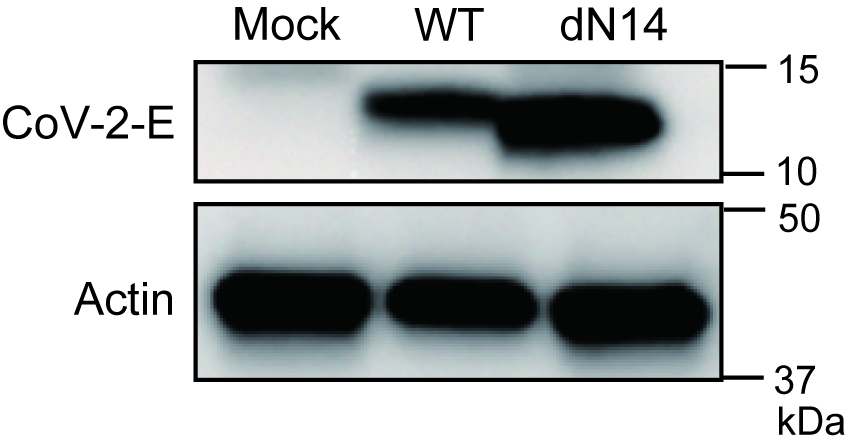

Supplement: Supplementary file 9 — Source data Fig. 8 [file 44319_2026_797_MOESM9_ESM.zip › Figure 8/Fig8D.tif]

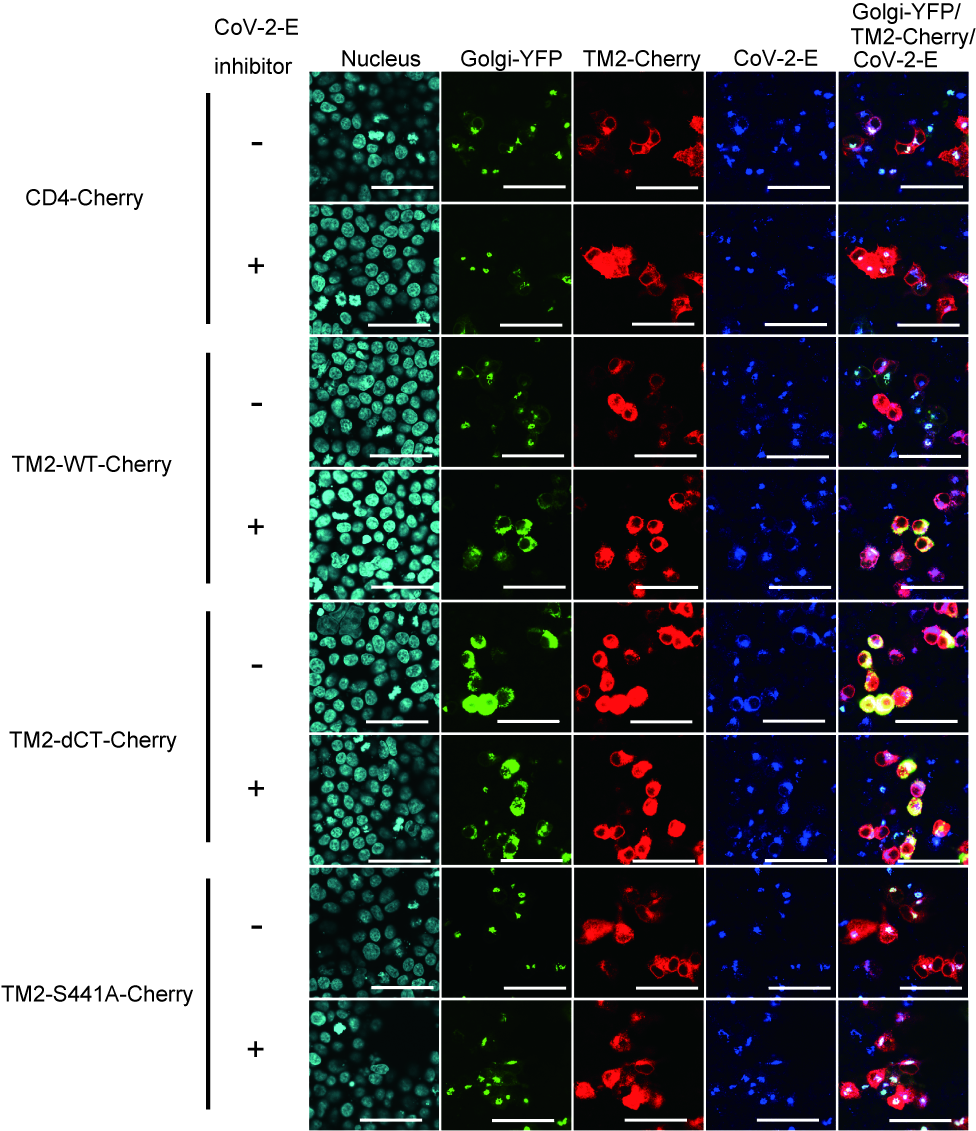

Supplement: Supplementary file 9 — Source data Fig. 8 [file 44319_2026_797_MOESM9_ESM.zip › Figure 8/Fig8E.tif]
