# Supplementary material for: TMPRSS2-induced Golgi disruption restricts the incorporation of virus envelope glycoproteins into virions
Source: EMBO Rep. 2026 May 19;27(12):3424–53. doi: 10.1038/s44319-026-00797-2 (PMC13303877; doi:10.1038/s44319-026-00797-2)
Supplement: Supplementary file 10 — Expanded View Figures [file 44319_2026_797_MOESM10_ESM.pdf]

Expanded View Figures

Figure EV1. Fluorescent protein-labeled TM2 and TM2 expression in cell lines.

(**A**) Infectivity of CoV-2-S pseudotyped lentiviruses was determined in ACE2- and TMPRSS2-WT (TM2-WT), TMPRSS2-WT-mCherry- (TM2-WT-Cherry)-, TMPRSS2-dCT-mCherry- (TM2-dCT-Cherry)-, TMPRSS2-S441A-mCherry- (TM2-S441A-Cherry)-, and/or TMPRSS2-WT-Wasabi- (TM2-WT-Wasabi)-expressing 293T cells (left and middle, the quantified results from a representative experiment in the two experiments independently conducted are expressed as mean ± SEM, $n = 6$). Infectivity of CoV-2-S pseudotyped lentiviruses was determined in ACE2- and TM2-WT-Cherry (with indicated ratio of plasmids) expressing 293 T cells (right, the quantified results are expressed as mean ± SEM of three separate experiments, $n = 3$). Representative data from three independent experiments are shown. (**B**) Immunoblotting analysis of cell lysate from 293 T cells expressing CoV-2-S (left) or HIV-1 Env (right) and TM2-WT-mCherry (TM2-WT) or TM2-S441A-mCherry (TM2-S441A). The ratio of CoV-2-S2 or gp160 to beta-actin was calculated based on band density (the quantified results are expressed as mean ± SEM of three separate experiments, $n = 3$). (**C**) Fluorescence microscopy analysis of A549-ACE2 and A549-ACE2-TMPRSS2-mCherry- (TM2)-expressing cells. (**D**) Infectivity of SARS-CoV-2 spike protein pseudotyped lentiviruses in ACE2- or ACE2- and TM2-expressing A549 cells (the quantified results from a representative experiment in the two experiments independently conducted are expressed as mean ± SEM, $n = 4$). (**E**) q-PCR analysis for TMPRSS2 (TM2) mRNA in indicated cell lines (A549-ACE2-TM2: A549-ACE2 cells stably expressing TM2; 293T-TM2: 293T cells transiently expressing TM2) (the quantified results from a representative experiment in the two experiments independently conducted are expressed as mean ± SEM, $n = 3$). (**F**) q-PCR analysis for TM2 mRNA in original Caco-2 (Caco-2 WT) and TM2 knockdown Caco-2 (Caco-2 TM2-KD) cells (the quantified results from a representative experiment in the two experiments independently conducted are expressed as mean ± SEM, $n = 3$). Scale bars indicate 50 μm. The results of Student's *t* tests are shown above the bars.

 © The Author(s)

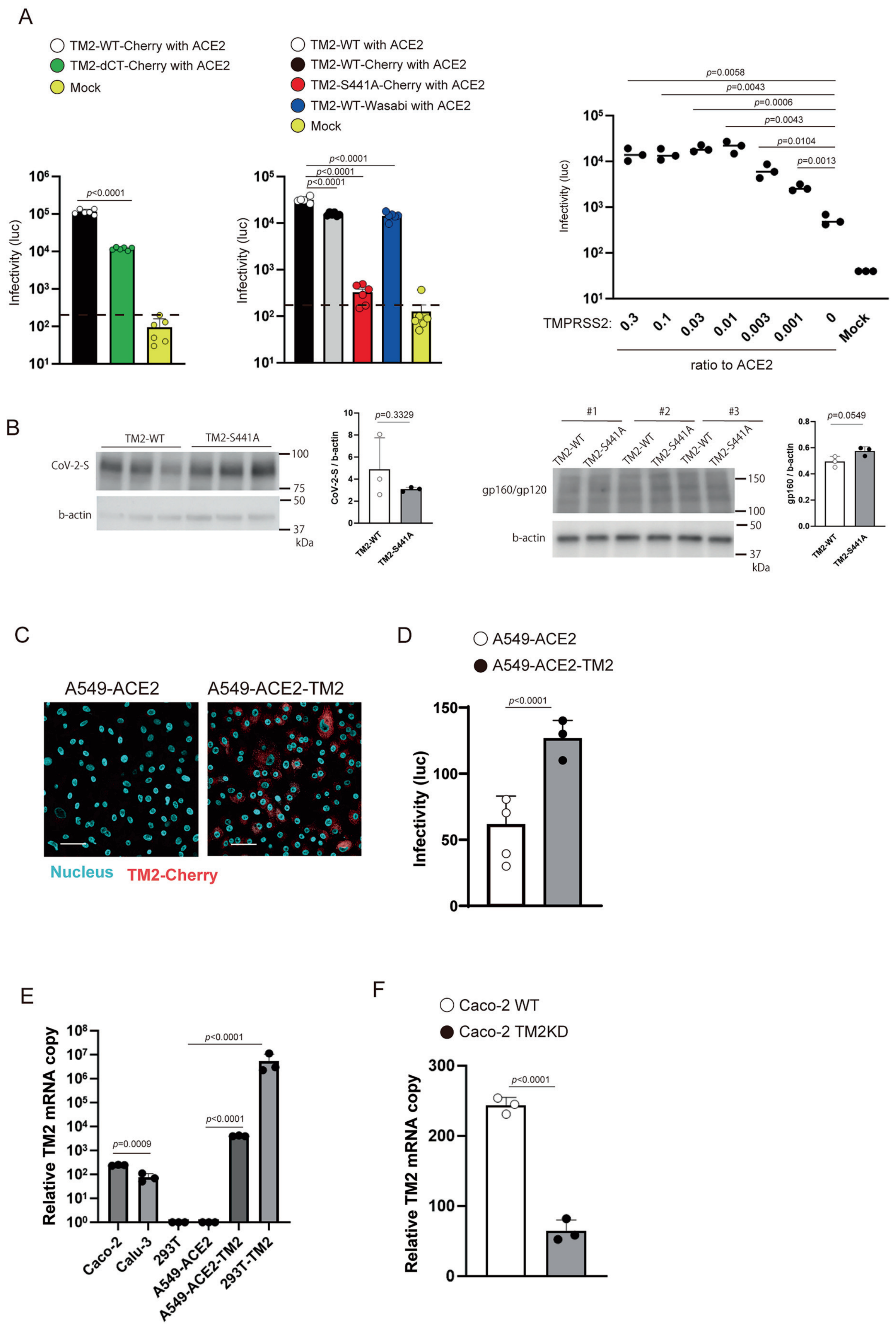
A
TM2-WT-Cherry with ACE2
TM2-dCT-Cherry with ACE2
Mock
TM2-WT with ACE2
TM2-WT-Cherry with ACE2
TM2-S441A-Cherry with ACE2
TM2-WT-Wasabi with ACE2
Mock
Infectivity (luc)
p<0.0001
p=0.0058
p=0.0043
p=0.0006
p=0.0043
p=0.0104
p=0.0013
TMPRSS2: 0.3 0.1 0.03 0.01 0.003 0.001 0 Mock
ratio to ACE2
B
TM2-WT
TM2-S441A
CoV-2-S
b-actin
kDa
p=0.3329
CoV-2-S / b-actin
#1 #2 #3
gp160/gp120
p=0.0549
gp160 / b-actin
C
A549-ACE2
A549-ACE2-TM2
Nucleus
TM2-Cherry
D
A549-ACE2
A549-ACE2-TM2
Infectivity (luc)
E
Relative TM2 mRNA copy
p=0.0009
Caco-2
Calu-3
293T
A549-ACE2
A549-ACE2-TM2
293T-TM2
F
Caco-2 WT
Caco-2 TM2KD
Relative TM2 mRNA copy

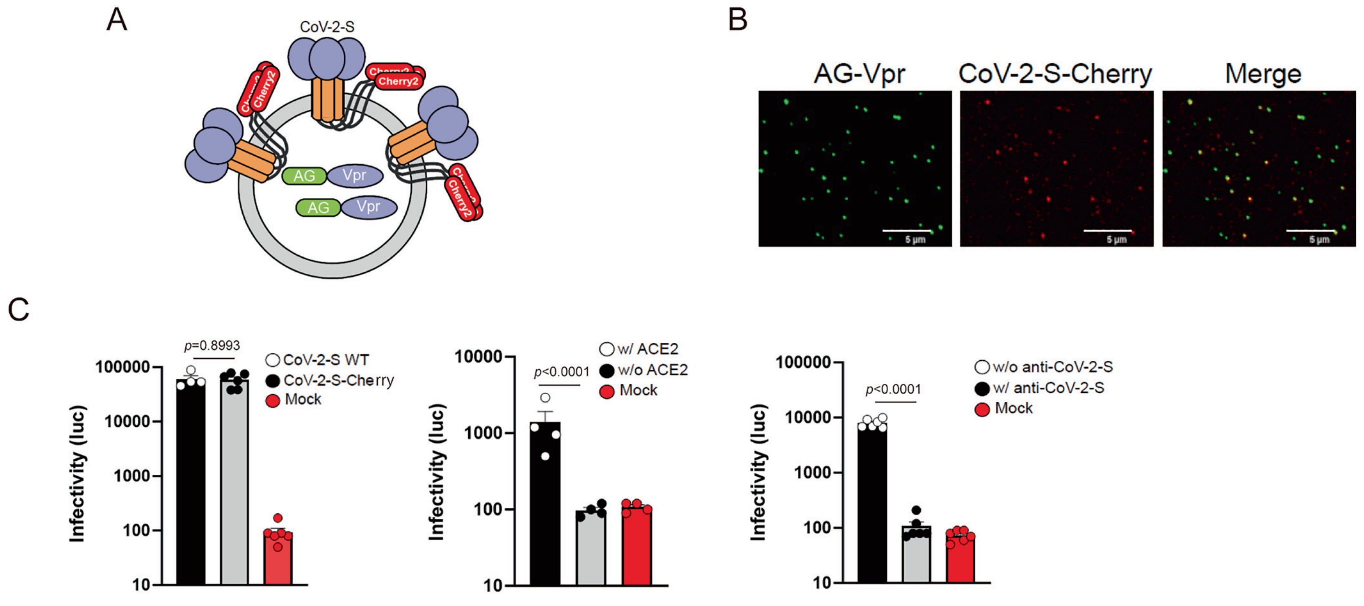

Figure EV2. Pseudoviruses carrying CoV-2-S labeled with fluorescent protein retain infectivity.

(**A**, **B**) Schematic model of CoV-2-S-mCherry carrying a virion labeled with Vpr-Azami Green (AG) (**A**) and immunofluorescence analysis of virions (**B**). (**C**) Infectivity of unlabeled CoV-2-S or CoV-2-S-Cherry carrying pseudotyped lentivirus in 293T cells, expressing ACE2 and TMPRSS2 (left) without ACE2 (middle) or with anti-CoV-2-S antibody (right); the quantified results from a representative experiment in the two experiments independently conducted are expressed as mean ± SEM, $n = 4$–6. The results of Student's t tests are shown above the bars.

 © The Author(s)

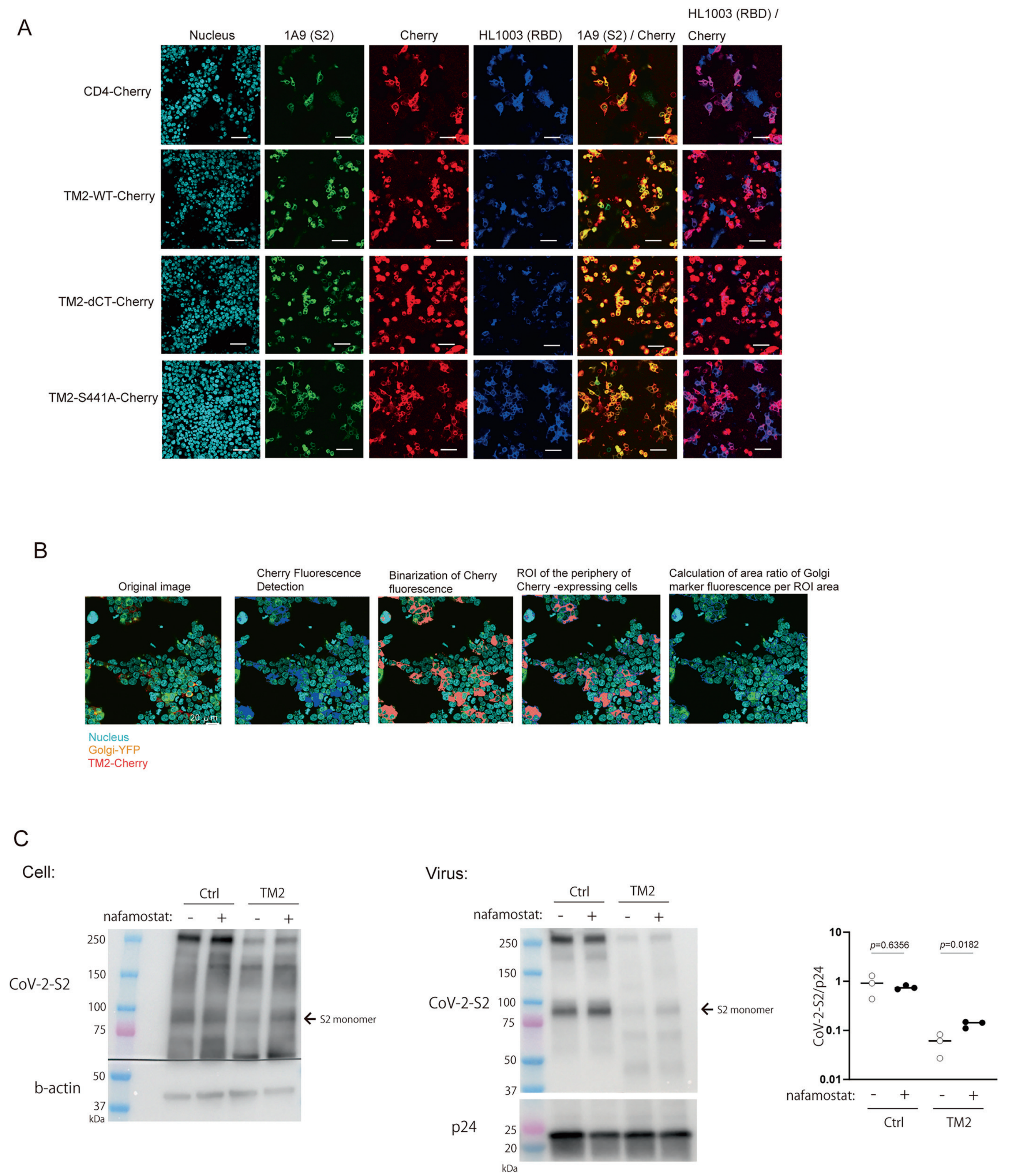
A
Nucleus
1A9 (S2)
Cherry
HL1003 (RBD)
1A9 (S2) / Cherry
HL1003 (RBD) / Cherry
CD4-Cherry
TM2-WT-Cherry
TM2-dCT-Cherry
TM2-S441A-Cherry
B
Original image
Cherry Fluorescence Detection
Binarization of Cherry fluorescence
ROI of the periphery of Cherry -expressing cells
Calculation of area ratio of Golgi marker fluorescence per ROI area
20 μm
Nucleus
Golgi-YFP
TM2-Cherry
C
Cell:
Ctrl
TM2
nafamostat: - + - +
250
150
100
75
50
37
kDa
CoV-2-S2
b-actin
S2 monomer
Virus:
Ctrl
TM2
nafamostat: - + - +
250
150
100
75
50
37
25
20
kDa
CoV-2-S2
p24
S2 monomer
p=0.6356
p=0.0182
10
1
0.1
0.01
CoV-2-S2/p24
nafamostat: - + - +
Ctrl
TM2

Figure EV3. Antibody accessibility to CoV-2-S in TM2-expressing cells.

(**A**) Low-magnification image of Fig. 3E. Immunofluorescence analysis of CoV-2-S structural changes using anti-S1 (HL1003) and S2 (1A9) antibodies in CD4-mCherry-, TMPRSS2-WT-mCherry- (TM2-WT)-, TMPRSS2-dCT-mCherry- (TM2-dCT)-, or TMPRSS2-S441A-mCherry- (TM2-S441A)-expressing 293T cells 24 h post-transfection. (**B**) Quantitative analysis of Golgi disruption. The region of interest (ROI) was determined by binarizing the mCherry fluorescence signal and detecting its perimeter to determine the area per TM2-Cherry-expressing cell. Golgi disruption was quantified by calculating the area occupied by Golgi marker fluorescence relative to the ROI area. (**C**) Nafamostat partially rescues the CoV-2-S incorporation into virions. Immunoblotting analysis of cell lysate (left) and CoV-2-S pseudotyped lentiviruses (middle) from 293T cells expressing CD4 (Ctrl), or TM2 with or without 100 µM of nafamostat. The ratio of CoV-2-S2 to p24 was calculated based on band density (right, the quantified results are expressed as mean ± SEM of three separate experiments, $n = 3$). The results of Student's t tests are shown above the bars.

 © The Author(s)

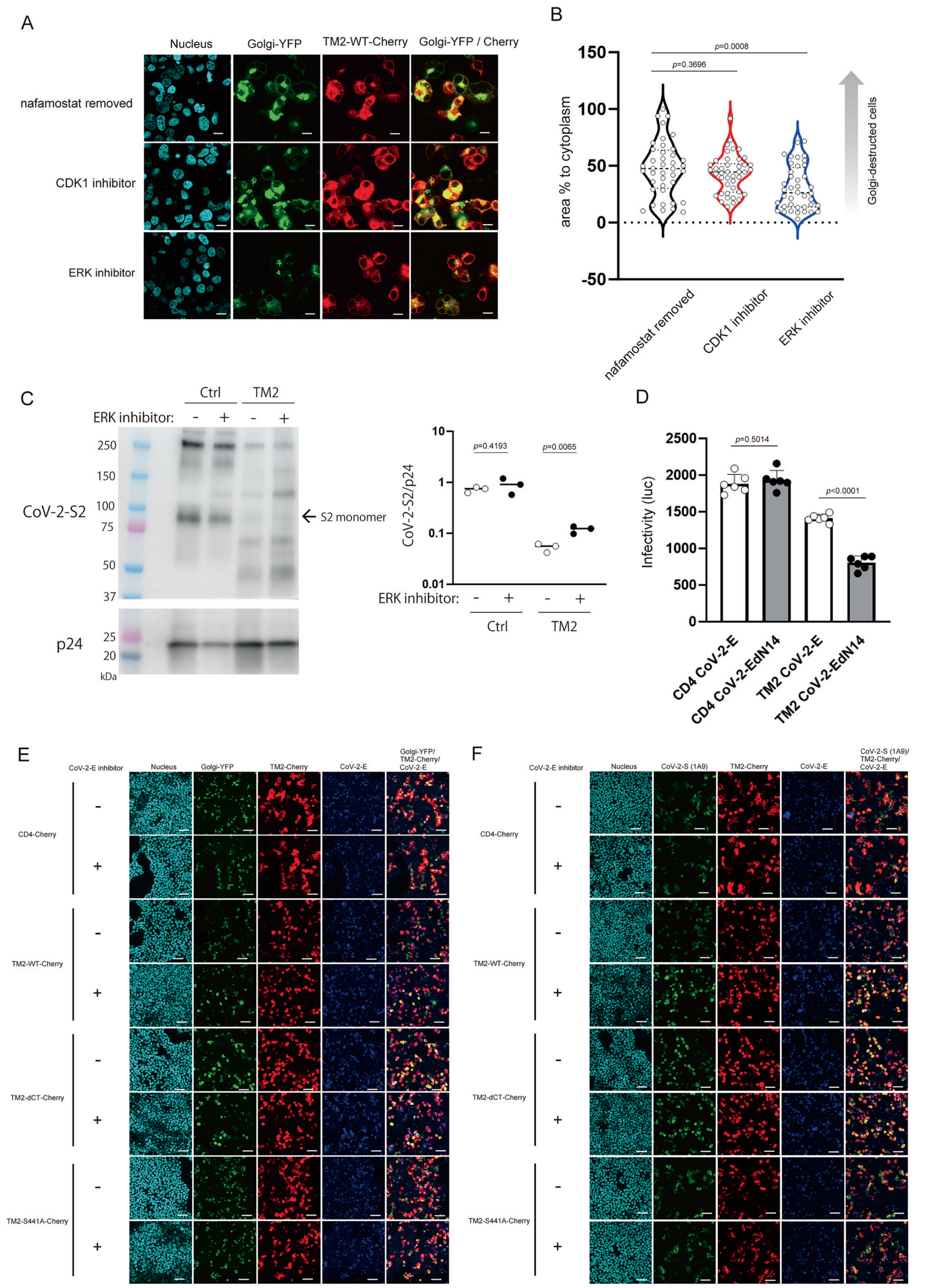
A
Nucleus
Golgi-YFP
TM2-WT-Cherry
Golgi-YFP / Cherry
nafamostat removed
CDK1 inhibitor
ERK inhibitor
B
p=0.0008
p=0.3696
area % to cytoplasm
Golgi-destructed cells
nafamostat removed
CDK1 inhibitor
ERK inhibitor
C
Ctrl
TM2
ERK inhibitor:
CoV-2-S2
S2 monomer
p24
kDa
CoV-2-S2/p24
p=0.4193
p=0.0065
D
p=0.5014
p<0.0001
Infectivity (luc)
CD4 CoV-2-E
CD4 CoV-2-EdN14
TM2 CoV-2-E
TM2 CoV-2-EdN14
E
CoV-2-E inhibitor
Nucleus
Golgi-YFP
TM2-Cherry
CoV-2-E
Golgi-YFP/ TM2-Cherry/ CoV-2-E
CD4-Cherry
TM2-WT-Cherry
TM2-dCT-Cherry
TM2-S441A-Cherry
F
CoV-2-S (1A9)
CoV-2-S (1A9)/ TM2-Cherry/ CoV-2-E

Figure EV4. Inhibition of TM2-induced Golgi disruption by an ERK inhibitor.

(**A**) Inhibition of TMPRSS2-induced Golgi disruption using ERK or CDK inhibitors, analyzed in 293T cells, transfected with Golgi-YFP (green) and TMPRSS2-WT-mCherry (TM2-WT-Cherry, red) and cultured for 24 h with 200 μM nafamostat. After removing nafamostat, cells were cultured with 20 μM ERK (20-233) or CDK1 (Ro 3306) inhibitors for 180 min before fixation. Scale bars indicate 10 μm. (**B**) Quantitative analysis of Golgi disruption in the images. Forty cells were randomly selected from at least three independent images and plotted. Representative data from two independent experiments are shown. (**C**) Immunoblotting analysis of CoV-2-S pseudotyped lentiviruses in 293T cells expressing CD4 (Ctrl), or TMPRSS2 (TM2) with or without 50 μM of ERK inhibitor. The ratio of CoV-2-S2 to p24 was calculated based on band density (right, the quantified results are expressed as mean ± SEM of three separate experiments, $n = 3$). (**D**) Inhibition of TM2 function by CoV-2-E. Luciferase activity for the infectivity of CoV-2-S pseudotyped lentiviruses produced in CD4-mCherry- (CD4)-, or TMPRSS2-mCherry- (TM2)-, and CoV-2-E or CoV-2-EdN14 expressing 293 T cells (the quantified results from a representative experiment in the two experiments independently conducted are expressed as mean ± SEM, $n = 6$). (**E**) Immunofluorescence analysis of Golgi-YFP (green) and CoV-2-E (blue) in CD4-mCherry (CD4-Cherry)-, TMPRSS2-WT-mCherry (TM2-WT-Cherry)-, TMPRSS2-dCT-mCherry (TM2-dCT-Cherry)-, or TMPRSS2-S441A-mCherry (TM2-S441A-Cherry)-expressing 293T cells cultured with CoV-2-E inhibitory peptide (+) or control peptide (−) at 24 h post-transfection. (**F**) Immunofluorescence analysis for CoV-2-S structural changes using anti-CoV-2-S2 (1A9, green) antibodies in CoV-2-S and CoV-2-E (blue) in CD4-Cherry-, TM2-WT-Cherry, TM2-dCT-Cherry, or TM2-S441A-Cherry (red) -expressing 293 T cells cultured with CoV-2-E inhibitory peptide (+) or control peptide (−) at 24 h post-transfection. Scale bars indicate 50 μm. The results of Student's *t* tests are shown above the bars.

 © The Author(s)
