# Supplementary material for: TMPRSS2-induced Golgi disruption restricts the incorporation of virus envelope glycoproteins into virions
Source: EMBO Rep. 2026 May 19;27(12):3424–53. doi: 10.1038/s44319-026-00797-2 (PMC13303877; doi:10.1038/s44319-026-00797-2)
Supplement: Supplementary file 1 — Peer Review File [file 44319_2026_797_MOESM1_ESM.pdf]

EMBO reports

TMPRSS2-induced Golgi disruption restricts the incorporation of virus envelope glycoproteins into virions

Sayuri Seki, Shigeyoshi Harada, Akiko Sugimoto-Ishige, Midori Unno, Machie Sakuma, Shinsuke Ito, Hiroyuki Yamamoto, Aki Tanabe, Saori Matsuoka, Chieko Makino-Okamura, Sewon Ki, Hidehiro Fukuyama, Michishige Harada, Kazuya Tsumagari, Koshi Imami, Takashi Saito, Masato Kubo, Tadaki Suzuki, Haruhiko Koseki, Tetsuro Matano, and Kosuke Miyauchi

Corresponding author(s): Kosuke Miyauchi (kosuke.miyauchi@riken.jp)

Review Timeline:

Submission Date:	29th Sep 25
Editorial Decision:	24th Oct 25
Revision Received:	20th Mar 26
Editorial Decision:	14th Apr 26
Revision Received:	17th Apr 26
Accepted:	27th Apr 26

Editor: Achim Breiling

Transaction Report:

(Note: With the exception of the correction of typographical or spelling errors that could be a source of ambiguity, letters and reports are not edited. Depending on transfer agreements, referee reports obtained elsewhere may or may not be included in this compilation. Referee reports are anonymous unless the Referee chooses to sign their reports.)

Dear Dr. Miyauchi,

Thank you for the submission of your manuscript to EMBO reports. I have now received reports from the three referees that were asked to evaluate your study, which can be found at the end of this email. As you will see, the referees think that these findings are of interest. However, they have several comments, concerns, and suggestions, indicating that a major revision of the manuscript is necessary to allow publication of the study in EMBO reports. As the reports are below, and all the referee concerns need to be addressed, I will not detail them here.

Given the constructive referee comments, I would thus like to invite you to revise your manuscript with the understanding that the concerns of the referees must be addressed in the revised manuscript and/or in a detailed point-by-point response. Acceptance of your manuscript will depend on a positive outcome of a second round of review. It is EMBO reports policy to allow a single round of revision only and acceptance of the manuscript will therefore depend on the completeness of your responses included in the next, final version of the manuscript.

Revised manuscripts should be submitted within three months of a request for revision. Please contact me to discuss the revision (also by video chat) if you have questions or comments regarding the revision, or should you need additional time.

When submitting your revised manuscript, please also carefully review the instructions that follow below.

PLEASE NOTE THAT upon resubmission revised manuscripts are subjected to an initial quality control prior to exposition to re-review. Upon failure in the initial quality control, the manuscripts are sent back to the authors, which may lead to delays. Frequent reasons for such a failure are the lack of the data availability section (please see below) and the presence of statistics based on n=2 (the authors are then asked to present scatter plots or provide more data points).

When submitting your revised manuscript, we will require:

1) a .docx formatted version of the final manuscript text (including legends for main figures, EV figures and tables), but without the figures included. Figure legends should be compiled at the end of the manuscript text.

2) individual production quality figure files as .eps, .tif, .jpg (one file per figure), of main figures and EV figures. Please upload these as separate, individual files upon re-submission.

The Expanded View format, which will be displayed in the main HTML of the paper in a collapsible format, has replaced the Supplementary information. You can submit up to 5 images as Expanded View. Please follow the nomenclature Figure EV1, Figure EV2 etc. The figure legend for these should be included in the main manuscript document file in a section called Expanded View Figure Legends after the main Figure Legends section. Additional Supplementary material should be supplied as a single pdf file labeled Appendix. The Appendix should have page numbers and needs to include a table of content on the first page (with page numbers) and legends for all content. Please follow the nomenclature Appendix Figure Sx, Appendix Table Sx etc. throughout the text, and also label the figures and tables according to this nomenclature.

For more details, please refer to our guide to authors:
http://www.embopress.org/page/journal/14693178/authorguide#manuscriptpreparation

Please consult our guide for figure preparation:
http://wol-prod-cdn.literatumonline.com/pb-assets/embo-site/EMBOPress_Figure_Guidelines_061115-1561436025777.pdf

See also the guidelines for figure legend preparation:
https://www.embopress.org/page/journal/14693178/authorguide#figureformat

3) a .docx formatted letter INCLUDING the reviewers' reports and your detailed point-by-point responses to their comments. As part of the EMBO Press transparent editorial process, the point-by-point response is part of the Review Process File (RPF), which will be published alongside your paper.

4) a complete author checklist, which you can download from our author guidelines (https://www.embopress.org/page/journal/14693178/authorguide). Please insert page numbers in the checklist to indicate where the requested information can be found in the manuscript. The completed author checklist will also be part of the RPF.

Please also follow our guidelines for the use of living organisms, and the respective reporting guidelines:
http://www.embopress.org/page/journal/14693178/authorguide#livingorganisms

5) that primary datasets produced in this study (e.g. RNA-seq, ChIP-seq, structural and array data) are deposited in an appropriate public database. If no primary datasets have been deposited, please also state this in a dedicated section (e.g. 'No

primary datasets have been generated and deposited'), see below.

See also: http://embor.embopress.org/authorguide#datadeposition

Please remember to provide a reviewer password if the datasets are not yet public.

The accession numbers and database should be listed in a formal "Data Availability" section that follows the model below. This is now mandatory (like the COI statement). Please note that the Data Availability Section is restricted to new primary data that are part of this study. This section is mandatory. As indicated above, if no primary datasets have been deposited, please state this in this section

Data availability

The datasets produced in this study are available in the following databases:

- RNA-Seq data: Gene Expression Omnibus GSE46843 (https://www.ncbi.nlm.nih.gov/geo/query/acc.cgi?acc=GSE46843)
- [data type]: [name of the resource] [accession number/identifier/doi] ([URL or identifiers.org/DATABASE:ACCESSION])

*** Note - All links should resolve to a page where the data can be accessed. ***

Moreover, I have these editorial requests:

6) We now request the publication of original source data with the aim of making primary data more accessible and transparent to the reader. You will receive a separate email with instructions for providing source data with your revised manuscript, including information how to upload and organize the files.

7) Our journal encourages inclusion of *data citations in the reference list* to directly cite datasets that were re-used and obtained from public databases. Data citations in the article text are distinct from normal bibliographical citations and should directly link to the database records from which the data can be accessed. In the main text, data citations are formatted as follows: "Data ref: Smith et al, 2001" or "Data ref: NCBI Sequence Read Archive PRJNA342805, 2017". In the Reference list, data citations must be labeled with "[DATASET]". A data reference must provide the database name, accession number/identifiers and a resolvable link to the landing page from which the data can be accessed at the end of the reference. Further instructions are available at: http://www.embopress.org/page/journal/14693178/authorguide#referencesformat

8) Regarding data quantification and statistics, please make sure that the number "n" for how many independent experiments were performed, their nature (biological versus technical replicates), the bars and error bars (e.g. SEM, SD) and the test used to calculate p-values is indicated in the respective figure legends (also for EV and Appendix figures). Please also check that all the p-values are explained in the legend, and that these fit to those shown in the figure. Please provide statistical testing where applicable. Please avoid the phrase 'independent experiment' but clearly state if these were biological or technical replicates. Please also indicate (e.g. with n.s.) if testing was performed, but the differences are not significant. In case n=2, please show the data as separate datapoints without error bars and statistics. See also: http://www.embopress.org/page/journal/14693178/authorguide#statisticalanalysis

If n<5, please show single datapoints for diagrams.

9) Please add scale bars of similar style and thickness to all microscopic images, using clearly visible black or white bars (depending on the background). Please place these in the lower right corner of the images themselves. Please do not write on or near the bars in the image but define the size in the respective figure legend.

10) Please also note our reference format:
http://www.embopress.org/page/journal/14693178/authorguide#referencesformat

11) We updated our journal's competing interests policy in January 2022 and request authors to consider both actual and perceived competing interests. Please review the policy https://www.embopress.org/competing-interests and update your competing interests if necessary. Please name this section 'Disclosure and Competing Interests Statement' and put it after the Acknowledgements section.

12) We now use CRediT to specify the contributions of each author in the journal submission system. CRediT replaces the author contribution section. Please use the free text box to provide more detailed descriptions and do NOT provide your final manuscript text file with an author contributions section. See also our guide to authors:
https://www.embopress.org/page/journal/14693178/authorguide#authorshipguidelines

13) All Materials and Methods need to be described in the main text using our 'Structured Methods' format, which is required for all research articles. According to this format, the Methods section should include a Reagents and Tools Table (listing key

reagents, experimental models, software, and relevant equipment and including their sources and relevant identifiers), uploaded as separate file, and a Methods section in which we encourage the authors to describe their methods using a step-by-step protocol format with bullet points, to facilitate the adoption of the methodologies across labs. More information on how to adhere to this format as well as downloadable templates (.doc) for the Reagents and Tools Table can be found in our author guidelines (section 'Structured Methods'):

https://www.embopress.org/page/journal/14693178/authorguide#manuscriptpreparation

14) Please order the manuscript sections like this, using only these names:
Title page - Abstract - Keywords - Introduction - Results - Discussion - Methods - Data availability section - Acknowledgements (please put here all the funding information) - Disclosure and Competing Interests Statement - References - Figure legends - Expanded View Figure legends

15) Please make sure that all the funding information is also entered into the online submission system and that it is complete and similar to the one in the acknowledgement section of the manuscript text file.

We would also welcome the submission of cover suggestions, or motifs to be used by our Graphics Illustrator in designing a cover.

As part of the EMBO publication's Transparent Editorial Process, EMBO reports publishes online a Review Process File (RPF) to accompany accepted manuscripts. This File will be published in conjunction with your paper and will include the referee reports, your point-by-point response and all pertinent correspondence relating to the manuscript.

You are able to opt out of this by letting the editorial office know (emboreports@embo.org). If you do opt out, the Review Process File link will point to the following statement: "No Review Process File is available with this article, as the authors have chosen not to make the review process public in this case."

I look forward to seeing the revised version of your manuscript when it is ready.

Please let me know if you have questions regarding the revision.

Yours sincerely,

Achim Breiling
Senior Editor
EMBO Reports

Referee #1:

Seki and colleagues examined the impact of TMPRSS2 expression in virus-producing cells. In brief, they report that TMPRSS2 reduces infectivity of progeny virus released from TMPRSS2 overexpressing cells. They further link this reduction in viral infectivity to reduced viral Spike or Env incorporation into the viral particles. They then show that TMPRSS2 overexpression induces trans-golgi disruption, likely through ERK signaling. TMPRSS2 mediated golgi disruption is counteracted by the E protein of SARS-CoV-2.

The results are of interest to the field, and the data look mostly sound. However, for certain figures only limited conclusions can be drawn based on the data presented and some additional controls or experiments would be needed to draw the conclusions stated in the text. The following points should be addressed.

Major points:

1 - The title reads "ERK-dependent TMPRSS2-induced Golgi disruption restricts the maturation of virus envelope proteins". In this work, two phenotypes are observed: (1) TMPRSS2 expression reduces viral progeny infectivity by reducing Spike incorporation. (2) TMPRSS2 disrupts trans-golgi through the ERK pathway.

No data shows that ERK-dependent TMPRSS2 disruption of the golgi causes the reduced infectivity of the viral progeny by reducing Spike incorporation. Showing that inhibiting ERK signaling rescues the infectivity of viral progeny in TMPRSS2+ cells is needed to draw the conclusion stated in the title.

2 - In the abstract, authors state "We found that TM2 inhibited biosynthesis of viral envelope proteins, resulting in lower

infectivity. We also found that the SARS-CoV-2 envelope protein (CoV-2-E) rescued the TM2-inhibited biosynthesis of CoV-2-S."

In Fig. 1D, and Fig. 3 C, the western blots show similar levels of Spike protein in the cell lysate between control and TMPRSS2 conditions, suggesting that TMPRSS2 does not act on protein biosynthesis but rather on incorporation of Spike into the virion, as seen in the blot (Fig. 1D). This conclusion should be reworded to closer reflect the data.

In addition, no data shows that inhibition of the ERK pathway results in an increased Spike incorporation into viral particles in TMPRSS2+ cells. Additional experiments showing that inhibition of the ERK pathway rescues Spike/Env incorporation into viral particles should be added.

Overall, the data are interesting and relevant to several fields of research, but the authors should review their writing to align it more closely with the data presented.

Point by point:

EV1. Expression levels of ACE2 and TMPRSS2 should be controlled. It would have been useful to include an ACE2 only control.

Fig. 1A:
- Expression levels of Spike and HIV-1 Env should be tested to determine if transfection of TMPRSS2 alters their expression level.
- P24 results showing overall production of lentiviral particles in the different conditions should be shown.
- The fact that infection is normalized by p24 ELISA, which is an important piece of information to interpret the graphs, is only mentioned in the materials and methods. This should be clearly stated in the results section or figure legend.

Line 109-110: Authors state: "These results indicate that even low levels of TM2 result in a marked inhibitory effect on viral infectivity." To conclude that A549-TM2+ cells express low levels of TMPRSS2, a staining comparing the expression with endogenous and transfected cells is necessary. Expression levels in the three models used (transfected 293T, stable A549 and Calu3) should be included.

Fig. 1C and E:
- Nafamostat is a broad serine protease inhibitor which does not only inhibit TMPRSS2 but many other serine proteases. TMPRSS2 may be involved but it does not exclude the action of other proteases (TMPRSS4, TMPRSS11D, TMPRSS13, etc). Knock-down or Knock-out of TMPRSS2 specifically are needed to make the conclusion line 115 and 122.
- A western blot showing the effect of Nafamostat on Spike processing and incorporation into viral particles is also missing.

Fig. 1D: Missing number of replicates of this blot. Missing molecular weights. Missing quantification of gag/S ratio.

Fig. 2D: This figure panel is very unconvincing for several reasons stated bellow, and should either be consolidated with strong evidence or removed.
(1) Same as previously, nafamostat is a broad-spectrum inhibitor. Knock-out or knock-down of TMPRSS2 would be necessary to draw the conclusion the authors state.
(2) Jurkat cells are not known to express TMPRSS2. A staining of TMPRSS2, to confirm its expression in their experimental model, rather than just a citation, is needed. In addition, the citation mentioned (Yao et al. 2022) shows that TMPRSS2 is expressed intracellularly in primary T cells rather than Jurkat.
(3) Infection levels of Jurkat cells are very low (0.24%), small variations thus lead to large fold change effects making interpreation difficult.

Fig. 3B: What does "background" stand for? A control with both antibodies on non-transfected cells and a control with cells transfected with only TMPRSS2 or Spike would have been nice to assess if the signal detected is specific.

Fig. 3C: Missing molecular weights.

Fig. 6. MG130 and TGN46 effect should be quantified.

Fig. 7. I don't understand why pERK is high at 14h post transfection in all conditions and lower at other timepoints? pERK/actin ratios should be quantified.

l. 45-47 - Authors write "SARS-CoV-2 infects host cells using angiotensin converting enzyme 2 (ACE2) and transmembrane protease serine 2 (TMPRSS2) as viral receptors during the viral entry phase.". TMPRSS2 is not a SARS-CoV-2 receptor but a priming protease.

Line 104 - Stable transduction of TMPRSS2 is still overexpression.

Line 106 - Figure EV2 C does not exist.

Referee #2:

Seki et al study the impact of TMPRSS2 on viral particle production. They find that TMPRSS2 reduces the infectivity of SARS-CoV-2 and HIV-1 during the viral production phase, which can be rescued by treatment of virus-producing cells with a TMPRSS2 inhibitor. They posit that TMPRSS2 disrupts the trans-Golgi by phosphorylating Golgi stacking proteins through ERK activation. This then prevents virion spike incorporation and structural maturation of the viral envelope proteins, causing lower viral infectivity. The viral E protein counteracts this TMPRSS2 activity to rescue Spike biosynthesis. Collectively, the authors show that viral proteins are regulated by host proteins and that viruses develop mechanisms to counteract this.

This is an interesting study and warrants publication, but there remains some aspects that the authors should address.

Major comments:
- All the experiments in Figure 1 require validation that equal amount of RNA/infection are in the producer cells. Additionally, the produced virions should be purified and split into two fractions: 1 fraction is preserved for the infection assay, while the second fraction is used for N protein level determination requiring viral particle disruption (i.e. N is inside the particle and inaccessible by ELISA antibodies). Currently, the authors state "Viruses in culture supernatant were evaluated by a SARS-CoV-2 nucleocapsid protein (NP) ELISA" without any other details.

- The role of the E protein in inhibiting TMPRSS2 needs to be validated in a viral production assay to support the authors' model. Currently both the pseudovirus (no E) and the virus (contains E) show similar phenotypes with regards to TMPRSS2, and the effect of E on inhibiting TMPRSS2 is only shown with regards to Golgi disruption.
- The authors show that ERK activation occurs post tranfection of even the control plasmid. Could they comment on why that is? And why is it transient?

Minor comments:
- The color of the dots, legends, and bars are confusing for all the figures. The bar should be colored in a similar color to the dots and the legend.

Referee #3:

In this manuscript, the authors investigated the impact of TMPRSS2 (TM2) expression in virion-producing cells on the infectivity of SARS-CoV-2 and HIV-1. Their findings revealed that TM2 expression broadly affects viral glycoprotein incorporation into virions, thereby reducing virion infectivity. Notably, the authors demonstrated that TM2 expression itself disrupts the trans-Golgi via the ERK pathway depending on its enzymatic activity, and SARS-CoV-2 E protein inhibits this TM2 effect on Golgi disruption.

The manuscript is comprehensive in summarizing their findings through multiple experiments, using different viruses and cell types. However, addressing certain points would be beneficial for potential readers.

Major points:
1. Most of the results are based on the TM2 overexpression system. Experiments using Calu-3 and Caco-2 with nafamostat suggest that physiological expression of TM2 can inhibit the production of infectious virions. However, nafamostat is a broad-spectrum serine protease inhibitor, rather than a TM2-specific inhibitor. The authors should clarify whether TM2 knockdown or knockout in Caco-2 or Calu-3 cells increases S-protein incorporation and virion infectivity.
2. The authors demonstrate that SARS-CoV-2 E protein inhibits TM2-induced Golgi disruption. Does the E protein rescue SARS-CoV-2 S incorporation into the virion and infectivity when the virus is produced in cells expressing TM2?
3. Related to point 2, experiments using authentic SARS-CoV-2 viruses with Caco-2 suggest that the E protein does not fully antagonize TM2 functions in virus-producing cells. Do Caco-2 cells express higher levels of TM2 compared with primary target cells? If there is any information and thoughts on this, it would be valuable to discuss this point.
4. Figure 3C shows that TM2 inhibits proteolytic cleavage and multimerization of the S protein in an enzymatic-activity-dependent manner. It is unclear whether similar effects occur in Figures 1D and 2B, but the bands below S2 may represent less-glycosylated forms. Previous studies (Nguyen et al., J Virol, 2021; Zhang et al., J Virol, 2021) from the Sodroski lab reported that uncleaved envelope glycoproteins (SARS-CoV-2 S and HIV-1 Env) tend to adopt more "open" conformations and are inefficiently incorporated into virions. Because cleaved Env/S species depend on Golgi-mediated processing for maturation and transport to the plasma membrane, Golgi disruption would be expected to reduce the surface expression of cleaved forms while allowing uncleaved, open-conformation species that bypass the Golgi to accumulate. Therefore, the increased detection of the "open" conformation may reflect a shift in the population of surface glycoproteins rather than an induced structural change. The authors should discuss whether the reduced incorporation is a consequence of this altered distribution caused by impaired

Golgi-dependent processing and trafficking or a direct conformational effect of TM2 expression.
5. Most microscopy analyses were conducted 24 hours post-transfection, whereas the Western blot showed only low-level ERK phosphorylation. Does Golgi disruption occur after a temporal delay following ERK phosphorylation, or does TM2 cause an irreversible disruption of the Golgi (e.g., through phosphorylation of stacking proteins) independent of transient pERK expression? The authors should clarify their interpretation of this temporal relationship.

Minor points:
1. For Western blotting experiments, molecular weight markers should be indicated. Quantification and statistical analysis should be provided for some blots (e.g., Figure 7A).
2. Please clarify the details of experimental settings in the material sections and/or figure legends. The plasmid ratios used for co-transfection were not specified except in Figure 2C.
3. In several sentences, the author describes HIV-1 Env and SARS-CoV-2 S as viral envelope proteins. To avoid confusion with SARS-CoV-2 E protein (envelope protein), the authors should use the term "viral envelope glycoprotein."
4. I suggest rephrasing "a non-overexpressed TM2 state" unless the authors confirm that the expression level is within the physiologically relevant range.
5. In Figure 2A, please specify whether TZM-bl and PM1R5 cells were used for SIV and HIV, respectively, or if Jurkat cells were employed as indicated in the scheme.

Referee #1:

Seki and colleagues examined the impact of TMPRSS2 expression in virus-producing cells. In brief, they report that TMPRSS2 reduces infectivity of progeny virus released from TMPRSS2 overexpressing cells. They further link this reduction in viral infectivity to reduced viral Spike or Env incorporation into the viral particles. They then show that TMPRSS2 overexpression induces trans-golgi disruption, likely through ERK signaling. TMPRSS2 mediated golgi disruption is counteracted by the E protein of SARS-CoV-2.

The results are of interest to the field, and the data look mostly sound. However, for certain figures only limited conclusions can be drawn based on the data presented and some additional controls or experiments would be needed to draw the conclusions stated in the text. The following points should be addressed.

> Thank you for the positive and valuable comments. We did additional experiments regarding the point you raised and have revised the descriptions accordingly.

Major points:

1 - The title reads "ERK-dependent TMPRSS2-induced Golgi disruption restricts the maturation of virus envelope proteins". In this work, two phenotypes are observed: (1) TMPRSS2 expression reduces viral progeny infectivity by reducing Spike incorporation. (2) TMPRSS2 disrupts trans-golgi through the ERK pathway.

No data shows that ERK-dependent TMPRSS2 disruption of the golgi causes the reduced infectivity of the viral progeny by reducing Spike incorporation. Showing that inhibiting ERK signaling rescues the infectivity of viral progeny in TMPRSS2+ cells is needed to draw the conclusion stated in the title.

> ERK activation is essential for SARS-CoV-2 replication, and ERK inhibitors have been reported to reduce SARS-CoV-2 infectivity (Hoffmann H et al 2023 *Front. Cell. Infect. Microbiol.*). On the other hand, we observed the partial rescue of the spike incorporation into virion by the ERK inhibitor in the additional experiment (Figure EV4C). Based on these results, we think that localized ERK phosphorylation in cells is required for TMPRSS2-mediated Golgi disassembly. It is widely known that ERK phosphorylation is required for the dissociation of Grasp55, which anchors the trans-Golgi network (Jesch SA et al 2001 *Mol. Biol. Cell*, Feinstein

TN et al 2008 *Mol. Biol. Cell*), thus, we believe this idea is reasonable. However, as Referee#1 pointed out, the original description could be misinterpreted as suggesting that the ERK activation inhibits SARS-CoV-2 replication. Therefore, we have revised the title as "TMPRSS2-induced Golgi disruption restricts the incorporation of virus envelope glycoproteins into virion".

2 - In the abstract, authors state "We found that TM2 inhibited biosynthesis of viral envelope proteins, resulting in lower infectivity. We also found that the SARS-CoV-2 envelope protein (CoV-2-E) rescued the TM2-inhibited biosynthesis of CoV-2-S."

In Fig. 1D, and Fig. 3 C, the western blots show similar levels of Spike protein in the cell lysate between control and TMPRSS2 conditions, suggesting that TMPRSS2 does not act on protein biosynthesis but rather on incorporation of Spike into the virion, as seen in the blot (Fig. 1D). This conclusion should be reworded to closer reflect the data.

The points have been corrected (Line 54-55, 89, 91, 269, 282, 296).

In addition, no data shows that inhibition of the ERK pathway results in an increased Spike incorporation into viral particles in TMPRSS2+ cells. Additional experiments showing that inhibition of the ERK pathway rescues Spike/Env incorporation into viral particles should be added.

As mentioned earlier, we conducted the experiment (Figure EV4C) and found the partial rescue of Spike incorporation into viral particles with ERK inhibitor. We added the related description (Line 244).

Overall, the data are interesting and relevant to several fields of research, but the authors should review their writing to align it more closely with the data presented.

Point by point:

EV1. Expression levels of ACE2 and TMPRSS2 should be controlled. It would have been useful to include an ACE2 only control.

We added data for hACE2 without TMPRSS2 control and different ratio of TMPRSS2 to hACE2 in Figure EV1A.

Fig. 1A:

- Expression levels of Spike and HIV-1 Env should be tested to determine if transfection of TMPRSS2 alters their expression level.

We tested the effect of TMPRSS2 to expression of CoV-2-S and HIV-1 env and found the effect is minimal (Figure EV1B). We added related descriptions (Line 103).

- P24 results showing overall production of lentiviral particles in the different conditions should be shown.

We added graphs for results of p24, p27 or NP ELISA into the figures (Figure 1A, B, C, E and F, Figure 2A and C).

- The fact that infection is normalized by p24 ELISA, which is an important piece of information to interpret the graphs, is only mentioned in the materials and methods. This should be clearly stated in the results section or figure legend.

We added information of p24, p27 or NP amount used for infection into the figure legends (Line 677, 700, 701, and 711).

Line 109-110: Authors state: "These results indicate that even low levels of TM2 result in a marked inhibitory effect on viral infectivity." To conclude that A549-TM2+ cells express low levels of TMPRSS2, a staining comparing the expression with endogenous and transfected cells is necessary. Expression levels in the three models used (transfected 293T, stable A549 and Calu3) should be included.

Thank you for pointing out this important issue. Since endogenous TMPRSS2 was difficult to detect by immunoblotting analysis (see below), we measured mRNA amount of TMPRSS2 in these cells by q-PCR (Figure EV1E).

Figure for referee with unpublished data and its description has been removed upon request by the authors.

Fig. 1C and E:

- Nafamostat is a broad serine protease inhibitor which does not only inhibit TMPRSS2 but many other serine proteases. TMPRSS2 may be involved but it does not exclude the action of other proteases (TMPRSS4, TMPRSS11D, TMPRSS13, etc). Knock-down or Knock-out of TMPRSS2 specifically are needed to make the conclusion line 115 and 122.

To answer this critical comment, we first checked mRNA amount of TMPRSS1, 4, 11D and 13 in Caco-2 cells, and detected mRNA of TMPRSS1, 4 and 13 as Reviewer#1 expected (see below). To evaluate impact of TMPRSS2 in production of infectious SARS-CoV-2, we developed TMPRSS2 knockdown Caco-2 cells. The replication efficacy of SARS-CoV-2 was significantly reduced with the reduction of TMPRSS2 expression (Figure 1F and EV1F). On the other hand, the infectivity of viruses after normalized with NP amount was marked increased (Figure 1F). This data suggests that TMPRSS2 has negative effects in production of infectious SARS-CoV-2 in Caco-2 cells. We added related descriptions (Line 124-130).

Figure for referee with unpublished data and its description has been removed upon request by the authors.

- A western blot showing the effect of Nafamostat on Spike processing and incorporation into viral particles is also missing.

We did this experiment and observed the partial rescue of Spike incorporation into virions with the nafamostat treatment (Figure EV3C).

Fig. 1D: Missing number of replicates of this blot. Missing molecular weights. Missing quantification of gag/S ratio.

We added quantitative data and molecular weights (Figure 1D).

Fig. 2D: This figure panel is very unconvincing for several reasons stated bellow, and should either be consolidated with strong evidence or removed.
(1) Same as previously, nafamostat is a broad-spectrum inhibitor. Knock-out or knock-down of TMPRSS2 would be necessary to draw the conclusion the authors state.
(2) Jurkat cells are not known to express TMPRSS2. A staining of TMPRSS2, to confirm its expression in their experimental model, rather than just a citation, is needed. In addition, the citation mentioned (Yao et al. 2022) shows that TMPRSS2 is expressed intracellularly in primary T cells rather than Jurkat.
(3) Infection levels of Jurkat cells are very low (0.24%), small variations thus lead to large fold change effects making interpreation difficult.

Thank you very much for pointing out this issue. For the reasons listed below, we wish to

follow Referee#1's advice and remove the original Figure 2D data.

(1) We measured mRNA amount of TMPRSS1, 2, 4 and 13 in Jurkat cells, and detected mRNA of TMPRSS1 and 13 but did not TMPRSS2 and 4 (Figure A below). We obtained TMPRSS2-Knock-out Jurkat cells (Ubigene, Austin, TX, USA) and observed the increasing of HIV-1 replication efficiency in compared with that in original Jurkat cells (Figure B below).

Figure for referee with unpublished data and its description has been removed upon request by the authors.

Figure for referee with unpublished data and its description has been removed upon request by the authors.

(2) We attempted LPS treatment in Jurkat cells, but we did not find stable elevation of TMPRSS2 mRNA after the LPS treatment (Figure C below).

Figure for referee with unpublished data and its description has been removed upon request by the authors.

(3) We revisited the culture and infection conditions and improved the infection level up to 0.85% (Figure B above).

Although we managed to show that the TMPRSS2 Knock-out in the Jurkat cell increases HIV-1 replication efficiency, we cannot exclude the possibility that other TMPRSS family proteins were involved given the low level of TMPRSS2 mRNA in Jurkat cells. Thus, we would like to remove original Figure 2D following Referee#1's suggestion. We believe this change did not affect the overall state of this paper.

Fig. 3B: What does "background" stand for? A control with both antibodies on non-transfected cells and a control with cells transfected with only TMPRSS2 or Spike would have been nice to assess if the signal detected is specific.

We have added controls Referee#1 pointed out and rearranged the graph (Figure 3B).

Fig. 3C: Missing molecular weights.

We added molecular weights (Figure 3C).

Fig. 6. MG130 and TGN46 effect should be quantified.

We added quantified data in Figure 6B and D.

Fig. 7. I don't understand why pERK is high at 14h post transfection in all conditions and lower at other timepoints? pERK/actin ratios should be quantified.

We revisited the experimental condition and improved the transfection efficiency of TMPRSS2 close to 90%. In this condition, the ratio of pERK to total ERK was continuously increased by TMPRSS2 expression (Figure 7A). We also quantified this data. We quantified pERK/actin ratios (See below).

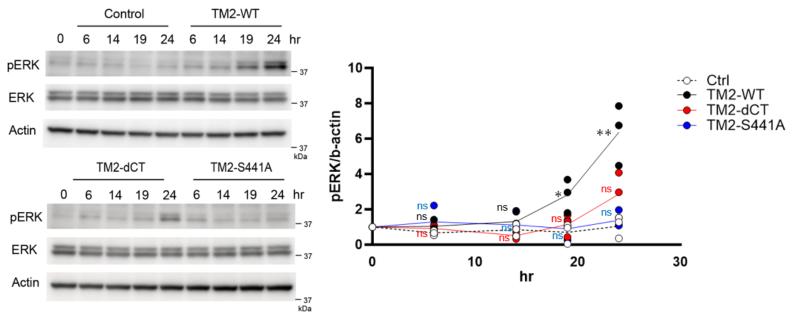

Phosphorylated and total ERK (pERK and ERK, respectively), and b-actin were detected in 293T cells expressing CD4-mCherry (Control), TMPRSS2-mCherry (TM2-WT), TMPRSS2-dCT-mCherry (TM2-dCT), or TMPRSS2-S441A-mCherry (TM2-S441A) at the indicated time (hr) post-transfection by immunoblotting analysis. The ratio of pERK to b-actin at 0 hr was set as 1, and the ratio of pERK to b-actin at each time point to that is shown in the graph (n=3). The results of Student's t-tests to value of Ctrl at each time point are shown above the points. ns, not significant; *, $p<0.05$; **, $p<0.01$

l. 45-47 - Authors write "SARS-CoV-2 infects host cells using angiotensin converting enzyme 2 (ACE2) and transmembrane protease serine 2 (TMPRSS2) as viral receptors during the viral entry phase.". TMPRSS2 is not a SARS-CoV-2 receptor but a priming protease.

Thank you for pointing that out. We have corrected the description (Line 47).

Line 104 - Stable transduction of TMPRSS2 is still overexpression.

We have corrected the description (Line 106).

Line 106 - Figure EV2 C does not exist.

We have corrected this.

Referee #2:

Seki et al study the impact of TMPRSS2 on viral particle production. They find that TMPRSS2 reduces the infectivity of SARS-CoV-2 and HIV-1 during the viral production phase, which can be rescued by treatment of virus-producing cells with a TMPRSS2 inhibitor. They posit that TMPRSS2 disrupts the trans-Golgi by phosphorylating Golgi stacking proteins through ERK activation. This then prevents virion spike incorporation and structural maturation of the viral envelope proteins, causing lower viral infectivity. The viral E protein counteracts this TMPRSS2 activity to rescue Spike biosynthesis. Collectively, the authors show that viral proteins are regulated by host proteins and that viruses develop mechanisms to counteract this.

This is an interesting study and warrants publication, but there remains some aspects that the authors should address.

Thank you for your encouraging comments. We have made corrections to the points you kindly pointed out.

Major comments:

- All the experiments in Figure 1 require validation that equal amount of RNA/infection are in the producer cells. Additionally, the produced virions should be purified and split into two fractions: 1 fraction is preserved for the infection assay, while the second fraction is used for N protein level determination requiring viral particle disruption (i.e. N is inside the particle and inaccessible by ELISA antibodies). Currently, the authors state "Viruses in culture supernatant were evaluated by a SARS-CoV-2 nucleocapsid protein (NP) ELISA" without any other details.

Thank you for pointing out this important issue. We have added the graphs for NP, p24, and p27 and clearly described in figure legends that these are used for normalization of virus particles to use infection assay (Figure 1 and 2, Line 677, 700, 701 and 711). We treated virions with RIPA lysis buffer for the ELISA. We added the description into Material and Methods (Line 405).
We also tested vRNA amount of CoV-2-S pseudotyped viruses produced by TMPRSS2 expressing 293T cells. The TMPRSS2 expression did not affect vRNA amount (see below).

Figure for referee with unpublished data and its description has been removed upon request by the authors.

- The role of the E protein in inhibiting TMPRSS2 needs to be validated in a viral production assay to support the authors' model. Currently both the pseudovirus (no E) and the virus (contains E) show similar phenotypes with regards to TMPRSS2, and the effect of E on inhibiting TMPRSS2 is only shown with regards to Golgi disruption.

We did infectivity assay for CoV-2-S pseudotyped virus with additional transfection of CoV-2-E protein and observed partial rescue of infectivity of the pseudotyped virus produced in TM2 expressing 293T cells (Figure EV4D). The effect of E protein on Spike incorporation could not be verified due to the low quantitative accuracy of immunoblotting analysis (see below). We believe that the simultaneous transfection of five plasmids, including the E protein expression plasmid, reduces the proportion of E protein expressing cells, making the experiment difficult.

Figure for referee with unpublished data and its description has been removed upon request by the authors.

- The authors show that ERK activation occurs post tranfection of even the control plasmid. Could they comment on why that is? And why is it transient?

We revisited the experimental condition and improved the transfection efficiency of TMPRSS2 close to 90%. In this condition, the ratio of pERK to total ERK was continuously increased by TMPRSS2 expression (Figure 7A). We also quantified this data. The transient increase in pERK with control plasmid is now negligible.

Minor comments:

- The color of the dots, legends, and bars are confusing for all the figures. The bar should be colored in a similar color to the dots and the legend.

We have corrected the color of bars (Figure 1, 2 and 3).

Referee #3:

In this manuscript, the authors investigated the impact of TMPRSS2 (TM2) expression in virion-producing cells on the infectivity of SARS-CoV-2 and HIV-1. Their findings revealed that TM2 expression broadly affects viral glycoprotein incorporation into virions, thereby reducing virion

infectivity. Notably, the authors demonstrated that TM2 expression itself disrupts the trans-Golgi via the ERK pathway depending on its enzymatic activity, and SARS-CoV-2 E protein inhibits this TM2 effect on Golgi disruption.

The manuscript is comprehensive in summarizing their findings through multiple experiments, using different viruses and cell types. However, addressing certain points would be beneficial for potential readers.

Thank you for your insightful and encouraging comments. We did additional experiments regarding the point you raised and have revised the descriptions accordingly.

Major points:

1. Most of the results are based on the TM2 overexpression system. Experiments using Calu-3 and Caco-2 with nafamostat suggest that physiological expression of TM2 can inhibit the production of infectious virions. However, nafamostat is a broad-spectrum serine protease inhibitor, rather than a TM2-specific inhibitor. The authors should clarify whether TM2 knockdown or knockout in Caco-2 or Calu-3 cells increases S-protein incorporation and virion infectivity.

To answer this point, we developed TMPRSS2 knockdown Caco-2 cells. The replication efficacy of SARS-CoV-2 was significantly reduced with the reduction of TMPRSS2 expression (Figure 1F and EV1F). On the other hand, the infectivity of viruses after normalized with NP amount was marked increased (Figure 1F). This data strongly suggests that TMPRSS2 has negative effects in production of infectious SARS-CoV-2 in Caco-2 cells. We added related descriptions (Line 124-130).

2. The authors demonstrate that SARS-CoV-2 E protein inhibits TM2-induced Golgi disruption. Does the E protein rescue SARS-CoV-2 S incorporation into the virion and infectivity when the virus is produced in cells expressing TM2?

We did infectivity assay for CoV-2-S pseudotyped virus with additional transfection of CoV-2-E protein and observed partial rescue of infectivity of the pseudotyped virus produced in TM2 expressing 293T cells (Figure EV4D). The effect of E protein on Spike incorporation could not be verified due to the low quantitative accuracy of immunoblotting analysis (see below). We believe that the simultaneous transfection of five plasmids, including the E protein expression plasmid, reduces the proportion of E protein expressing cells, making the experiment difficult.

Figure for referee with unpublished data and its description has been removed upon request by the authors.

3. Related to point 2, experiments using authentic SARS-CoV-2 viruses with Caco-2 suggest that the E protein does not fully antagonize TM2 functions in virus-producing cells. Do Caco-2 cells express higher levels of TM2 compared with primary target cells? If there is any information and thoughts on this, it would be valuable to discuss this point.

Thank you for mentioning this important point. Since endogenous TMPRSS2 was difficult to detect by immunoblotting analysis (Figure A below), we measured TMPRSS2 mRNA amount in these cells by the q-PCR analysis. As Referee#3 predicted, TMPRSS2 expression level in Caco-2 is higher than that in Calu-3 (Figure B below). We added related descriptions (Line 124-127).

Figure for referee with unpublished data and its description has been removed upon request by the authors.

Figure for referee with unpublished data and its description has been removed upon request by the authors.

4. Figure 3C shows that TM2 inhibits proteolytic cleavage and multimerization of the S protein in an enzymatic-activity-dependent manner. It is unclear whether similar effects occur in Figures 1D and 2B, but the bands below S2 may represent less-glycosylated forms. Previous studies (Nguyen et al., J Virol, 2021; Zhang et al., J Virol, 2021) from the Sodroski lab reported that uncleaved envelope glycoproteins (SARS-CoV-2 S and HIV-1 Env) tend to adopt more "open" conformations and are inefficiently incorporated into virions. Because cleaved Env/S species depend on Golgi-mediated processing for maturation and transport to the plasma membrane, Golgi disruption would be expected to reduce the surface expression of cleaved forms while allowing uncleaved, open-conformation species that bypass the Golgi to accumulate. Therefore, the increased detection of the "open" conformation may reflect a shift in the population of surface glycoproteins rather than an induced structural change. The authors should discuss whether the reduced incorporation is a consequence of this altered distribution caused by impaired Golgi-dependent processing and trafficking or a direct conformational effect of TM2 expression.

Thank you for pointing out this important issue. To investigate the effect of TM2 on CoV-2-S intracellular transport, we performed cellular fractionation analysis. Compared to the S441A mutant, TM2-WT showed a significant reduction in CoV-2-S transport to the plasma membrane fraction (Figure 6A). We also performed an analysis for glycan modifications using Endo H and PNGase, but no significant changes due to TM2 expression were observed (see below). Based on these results we have added related descriptions (Line 210-216).

Figure for referee with unpublished data and its description has been removed upon request by the authors.

5. Most microscopy analyses were conducted 24 hours post-transfection, whereas the Western blot showed only low-level ERK phosphorylation. Does Golgi disruption occur after a temporal delay following ERK phosphorylation, or does TM2 cause an irreversible disruption of the Golgi (e.g., through phosphorylation of stacking proteins) independent of transient pERK expression? The authors should clarify their interpretation of this temporal relationship.

We revisited the experimental condition and improved the transfection efficiency of TMPRSS2 close to 90%. In this condition, the ratio of pERK to total ERK was continuously increased by TMPRSS2 expression (Figure 7A). We also quantified this data. The transient increase in pERK with control plasmid is now negligible.

Minor points:

1. For Western blotting experiments, molecular weight markers should be indicated. Quantification and statistical analysis should be provided for some blots (e.g., Figure 7A).

We have added molecular weights (Figure 1D, 2B, 3C, 7A and 8D: marker lanes can be verified in the original source data) and quantified data (Figure 1D, 2B and 7A).

2. Please clarify the details of experimental settings in the material sections and/or figure legends. The plasmid ratios used for co-transfection were not specified except in Figure 2C.

We have added the plasmid ratio (Figure 1A, 1C and 2A).

3. In several sentences, the author describes HIV-1 Env and SARS-CoV-2 S as viral envelope proteins. To avoid confusion with SARS-CoV-2 E protein (envelope protein), the authors should use the term "viral envelope glycoprotein."

We have corrected the description (Line 52, 55, 90, 174, 191, 280, 282, 296 and 306).

4. I suggest rephrasing "a non-overexpressed TM2 state" unless the authors confirm that the expression level is within the physiologically relevant range.

We have changed the description (Line 139).

5. In Figure 2A, please specify whether TZM-bl and PM1R5 cells were used for SIV and HIV, respectively, or if Jurkat cells were employed as indicated in the scheme.

We have modified the Figure 2A.

Dear Dr. Miyauchi,

Thank you for the submission of your revised manuscript to our editorial offices. I have now received the reports from the three referees that were asked to re-evaluate the study, you will find below. As you will see, the referees now fully support the publication of the study in EMBO reports.

Before we can proceed with formal acceptance, I have the editorial requests below I ask you to address in a final revised manuscript. Please also provide a final p-b-p-response to the editorial requests.

Editorial requests:

- Please provide the abstract written in present tense throughout.

- It seems you used live virus strains in this study. Please add a paragraph to the methods section (titled 'Biosafety') providing details on where and how biosafety-relevant experiments were performed and that these were approved, and by whom (institution, government).

- Please check again that the number "n" for how many independent experiments were performed, their nature (biological versus technical replicates), the bars and error bars (e.g. SEM, SD) and the test used to calculate p-values is indicated in the respective figure legends (main and EV figures). Please also check that all the p-values are explained in the legend, and that these fit to those shown in the figure. Please provide statistical testing where applicable. Please avoid the phrase 'independent experiment' but clearly state if these were biological or technical replicates. Please also indicate (e.g. with n.s.) if testing was performed, but the differences are not significant. In case n=2, please show the data as separate datapoints without error bars and statistics. See also:

https://link.springer.com/journal/44319/submission-guidelines#cms-Figure-and-data-presentation

If n<5, please show single datapoints for diagrams. Moreover:
- Please note that the exact p values are not provided in the legends of figures 1A, C, D, E, F; 2A-C; 3B, F, H; 4E, 5B, G; 6B, D; 7A, E; 8B, F; EV1 A, D, E, F; EV2 C, EV3 C, EV4 B-D
- Please note that the error bars are not defined in the legends of figures 1A-F; 2A-C; 3B, H; EV2 C; EV1A, B, D, E, F; EV4 D.

- Please remove now the referee access information from the data availability section. Please make sure that the dataset is public latest upon online publication of the study and add a final direct link to the dataset.

- Please make sure that each figure panel is called out separately and that the panels are called out sequentially. Presently, it seems there are no callout for Fig. EV5. Please check.

- Please add the primer information presently shown in the Appendix (Tables S1 and S2) directly to the Reagents & Tools Table. Please add callouts for the Reagents & Tools table where applicable. Please remove the mention of EV Tables 1 and 2 from the Reagents and Tools Table. Finally, please remove the Appendix file from the submission.

- Please make sure that all the funding information is also entered into the online submission system and that it is complete and similar to the one in the acknowledgement section of the manuscript text file. Presently, there is a discrepancy between grant number 21K06570 in the manuscript text file and 21K0657 in the submission system. Please check.

- Please confirm that for all Western blot panels the lanes shown (including the loading control) were run on the same gel as the other proteins detected. I am not sure this is always the case. Please note that we discourage comparisons between samples on different gels/blots, even if the samples derive from one experiment, as confounding factors reduce comparability. If unavoidable, the figure legend must state that the samples derive from the same experiment and that gels/blots were processed in parallel. If a 'representative' loading control is shown for multiple gels/blots, the intra-gel controls should be shown in the source data files, and the figure legends should describe the data displayed accurately. See our author guidelines:

https://link.springer.com/journal/44319/submission-guidelines#cms-Figure-and-data-presentation (section 'Electrophoretic gels and blots').

- We noted an image reuse between several panels of Figure 3E and Figure EV3A. It seems panels in 3E show enlargements of a subset of panels in EV3A. Please clearly indicate this in the respective figure legend.

- Thanks for providing the source data. Please upload the source data as one folder per final main figure, grouping together the separate files (in excel format) for the panels, and ZIPed together. For the numerical data each Excel file should be labelled with the panels it contains, e.g. "Figure 1ABCDEF". Please do that for all files where applicable.

In addition, I would need from you uploaded separately:
- a short, two-sentence summary of the manuscript (not more than 35 words).
- two to four short (!) bullet points highlighting the key findings of your study (two lines each).
- a schematic summary figure as separate file that provides a sketch of the major findings (not a data image) in jpeg or tiff format (with the exact width of 550 pixels and the exact height of 300 pixels) that can be used as a visual synopsis on our website.

I look forward to seeing the further revised version of your manuscript when it is ready. Please let me know if you have questions regarding the revision.

Best,

Achim Breiling
Senior Editor
EMBO Reports

Referee #1:

The authors have addressed all my comments and provided new data where requested.

Minor comment regarding the point-by-point format: The line numbers are sometimes incorrect, which makes checking corrections tedious. A version with tracked changes (highlighted in color) for major text revisions would have been appreciated. Additionally, embedding the new figures directly within the point-by-point response would have streamlined the review process.

Referee #2:

The authors have addressed all my concerns.

Referee #3:

The authors have addressed my points and accordingly revised the manuscript.

The authors have addressed all minor editorial requests.

Dr. Kosuke Miyauchi
RIKEN
IMS
Japan

Dear Dr. Miyauchi,

I am very pleased to accept your manuscript for publication in the next available issue of EMBO reports. Thank you for your contribution to our journal.

Your manuscript will be processed for publication by EMBO Press. It will be copy edited and you will receive page proofs prior to publication. Please note that you will be contacted by Springer Nature Author Services to complete licensing and payment information.

You may qualify for financial assistance for your publication charges - either via a Springer Nature fully open access agreement or an EMBO initiative. Check your eligibility: https://link.springer.com/journal/44319/how-to-publish-with-us

Should you be planning a Press Release on your article, please get in contact with embo_production@springernature.com as early as possible in order to coordinate publication and release dates.

If you have any questions, please do not hesitate to contact the Editorial Office. Thank you for your contribution to EMBO Reports.

Yours sincerely,

Achim Breiling
Senior Editor
EMBO Reports

>>> Please note that it is EMBO Reports policy for the transcript of the editorial process (containing referee reports and your response letter) to be published as an online supplement to each paper. If you do NOT want this, you will need to inform the Editorial Office via email immediately. More information is available here: https://link.springer.com/partners/embo-press/editorial-policies#Peer%20review

EMBO Press Author Checklist

Corresponding Author Name: Kosuke Miyauchi	
Journal Submitted to: EMBO Reports	
Manuscript Number: EMBOR-2025-62850	

USEFUL LINKS FOR COMPLETING THIS FORM
The EMBO Journal - Author Guidelines
EMBO Reports - Author Guidelines
Molecular Systems Biology - Author Guidelines
EMBO Molecular Medicine - Author Guidelines

Reporting Checklist for Life Science Articles (updated January
This checklist is adapted from Materials Design Analysis Reporting (MDAR) Checklist for Authors. MDAR establishes a minimum set of requirements in transparent reporting in the life sciences (see Statement of Task: 10.31222/osf.io/9sm4x). Please follow the journal's guidelines in preparing your
Please note that a copy of this checklist will be published alongside your article.

Abridged guidelines for figures
1. Data
The data shown in figures should satisfy the following conditions:
- the data were obtained and processed according to the field's best practice and are presented to reflect the results of the experiments in an accurate and unbiased manner.
- ideally, figure panels should include only measurements that are directly comparable to each other and obtained with the same assay.
- plots include clearly labeled error bars for independent experiments and sample sizes. Unless justified, error bars should not be shown for technical
- if n<5, the individual data points from each experiment should be plotted. Any statistical test employed should be justified.
- Source Data should be included to report the data underlying figures according to the guidelines set out in the authorship guidelines on Data

2. Captions
Each figure caption should contain the following information, for each panel where they are relevant:
- a specification of the experimental system investigated (eg cell line, species name).
- the assay(s) and method(s) used to carry out the reported observations and measurements.
- an explicit mention of the biological and chemical entity(ies) that are being measured.
- an explicit mention of the biological and chemical entity(ies) that are altered/varied/perturbed in a controlled manner.
- the exact sample size (n) for each experimental group/condition, given as a number, not a range;
- a description of the sample collection allowing the reader to understand whether the samples represent technical or biological replicates (including how many animals, litters, cultures, etc.).
- a statement of how many times the experiment shown was independently replicated in the laboratory.
- definitions of statistical methods and measures:
  - common tests, such as t-test (please specify whether paired vs. unpaired), simple χ^2 tests, Wilcoxon and Mann-Whitney tests, can be unambiguously identified by name only, but more complex techniques should be described in the methods section;
  - are tests one-sided or two-sided?
  - are there adjustments for multiple comparisons?
  - exact statistical test results, e.g., P values = x but not P values < x;
  - definition of 'center values' as median or average;
  - definition of error bars as s.d. or s.e.m.

Please complete ALL of the questions below.
Select "Not Applicable" only when the requested information is not relevant for your study.

Materials

Newly Created Materials	Information included in the manuscript?	In which section is the information available? (Reagents and Tools Table, Materials and Methods, Figures, Data Availability Section)
New materials and reagents need to be available; do any restrictions apply?	Not Applicable	

Antibodies	Information included in the manuscript?	In which section is the information available? (Reagents and Tools Table, Materials and Methods, Figures, Data Availability Section)
For **antibodies** provide the following information:<br>- Commercial antibodies: RRID (if possible) or supplier name, catalogue number and or/clone number<br>- Non-commercial: RRID or citation	Yes	Reagents and Tools Table

DNA and RNA sequences	Information included in the manuscript?	In which section is the information available? (Reagents and Tools Table, Materials and Methods, Figures, Data Availability Section)
**Short novel DNA or RNA including primers, probes**: provide the sequences.	Yes	Appendix

Cell materials	Information included in the manuscript?	In which section is the information available? (Reagents and Tools Table, Materials and Methods, Figures, Data Availability Section)
**Cell lines:** Provide species information, strain. Provide accession number in repository **OR** supplier name, catalog number, clone number, and/**OR** RRID.	Yes	Reagents and Tools Table
**Primary cultures:** Provide species, strain, sex of origin, genetic modification status.	Not Applicable	
Report if the cell lines were recently **authenticated** (e.g., by STR profiling) and tested for mycoplasma contamination.	Not Applicable	

Experimental animals	Information included in the manuscript?	In which section is the information available? (Reagents and Tools Table, Materials and Methods, Figures, Data Availability Section)
**Laboratory animals or Model organisms:** Provide species, strain, sex, age, genetic modification status. Provide accession number in repository **OR** supplier name, catalog number, clone number, **OR** RRID.	Not Applicable	
**Animal observed in or captured from the field:** Provide species, sex, and age where possible.	Not Applicable	
Please detail **housing and husbandry conditions**.	Not Applicable	

Plants and microbes	Information included in the manuscript?	In which section is the information available? (Reagents and Tools Table, Materials and Methods, Figures, Data Availability Section)
**Plants:** provide species and strain, ecotype and cultivar where relevant, unique accession number if available, and source (including location for collected wild specimens).	Not Applicable	
**Microbes:** provide species and strain, unique accession number if available, and source.	Yes	Reagents and Tools Table

Human research participants	Information included in the manuscript?	In which section is the information available? (Reagents and Tools Table, Materials and Methods, Figures, Data Availability Section)
If collected and within the bounds of privacy constraints report on age, sex and gender or ethnicity for all study participants.	Not Applicable	

Core facilities	Information included in the manuscript?	In which section is the information available? (Reagents and Tools Table, Materials and Methods, Figures, Data Availability Section)
If your work benefited from core facilities, was their service mentioned in the acknowledgments section?	Not Applicable	

Design

Study protocol	Information included in the manuscript?	In which section is the information available? (Reagents and Tools Table, Materials and Methods, Figures, Data Availability Section)
If study protocol has been **pre-registered, provide DOI in the manuscript**. For clinical trials, provide the trial registration number **OR** cite DOI.	Not Applicable	
Report the **clinical trial registration number** (at ClinicalTrials.gov or equivalent), where applicable.	Not Applicable	

Laboratory protocol	Information included in the manuscript?	In which section is the information available? (Reagents and Tools Table, Materials and Methods, Figures, Data Availability Section)
Provide DOI OR other citation details if **external detailed step-by-step protocols** are available.	Not Applicable	

Experimental study design and statistics	Information included in the manuscript?	In which section is the information available? (Reagents and Tools Table, Materials and Methods, Figures, Data Availability Section)
Include a statement about **sample size** estimate even if no statistical methods were used.	Not Applicable	
Were any steps taken to minimize the effects of subjective bias when allocating animals/samples to treatment (e.g. **randomization procedure**)? If yes, have they been described?	Not Applicable	
Include a statement about **blinding** even if no blinding was done.	Not Applicable	
Describe **inclusion/exclusion criteria** if samples or animals were excluded from the analysis. Were the criteria pre-established?<br><br>If sample or data points were omitted from analysis, report if this was due to attrition or intentional exclusion and provide justification.	Not Applicable	
For every figure, are **statistical tests** justified as appropriate? Do the data meet the assumptions of the tests (e.g., normal distribution)? Describe any methods used to assess it. Is there an estimate of variation within each group of data? Is the variance similar between the groups that are being statistically compared?	Not Applicable	

Sample definition and in-laboratory replication	Information included in the manuscript?	In which section is the information available? (Reagents and Tools Table, Materials and Methods, Figures, Data Availability Section)
In the figure legends: state number of times the experiment was **replicated** in laboratory.	Yes	Figure legends
In the figure legends: define whether data describe **technical or biological replicates**.	Yes	Figure legends

Ethics

Ethics	Information included in the manuscript?	In which section is the information available? (Reagents and Tools Table, Materials and Methods, Figures, Data Availability Section)
Studies involving **human participants**: State details of **authority granting ethics approval** (IRB or equivalent committee(s), provide reference number for approval.	Not Applicable	
Studies involving **human participants**: Include a statement confirming that **informed consent** was obtained from all subjects and that the experiments conformed to the principles set out in the WMA Declaration of Helsinki and the Department of Health and Human Services Belmont Report.	Not Applicable	
Studies involving **human participants:** For publication of **patient photos**, include a statement confirming that consent to publish was obtained.	Not Applicable	
Studies involving experimental **animals**: State details of **authority granting ethics approval** (IRB or equivalent committee(s), provide reference number for approval. Include a statement of compliance with ethical regulations.	Not Applicable	
Studies involving **specimen and field samples:** State if relevant **permits** obtained, provide details of authority approving study; if none were required, explain why.	Not Applicable	

Dual Use Research of Concern (DURC)	Information included in the manuscript?	In which section is the information available? (Reagents and Tools Table, Materials and Methods, Figures, Data Availability Section)
Could your study fall under dual use research restrictions? Please check biosecurity documents and list of **select agents and toxins** (CDC): https://www.selectagents.gov/sat/list.htm	Not Applicable	
If you used a select agent, is the security level of the lab appropriate and reported in the manuscript?	Not Applicable	
If a study is subject to dual use research of concern regulations, is the name of the **authority granting approval and reference number** for the regulatory approval provided in the manuscript?	Not Applicable	

Reporting

The MDAR framework recommends adoption of discipline-specific guidelines, established and endorsed through community initiatives. Journals have their own policy about requiring specific guidelines and recommendations to complement MDAR.

Adherence to community standards	Information included in the manuscript?	In which section is the information available? (Reagents and Tools Table, Materials and Methods, Figures, Data Availability Section)
State if relevant guidelines or checklists (e.g., **ICMJE, MIBBI, ARRIVE, PRISMA**) have been followed or provided.	Not Applicable	
For **tumor marker prognostic studies,** we recommend that you follow the **REMARK** reporting guidelines (see link list at top right). See author guidelines, under 'Reporting Guidelines'. Please confirm you have followed these guidelines.	Not Applicable	
For **phase II and III randomized controlled trials**, please refer to the **CONSORT** flow diagram (see link list at top right) and submit the CONSORT checklist (see link list at top right) with your submission. See author guidelines, under 'Reporting Guidelines'. Please confirm you have submitted this list.	Not Applicable	

Data Availability

Data availability	Information included in the manuscript?	In which section is the information available? (Reagents and Tools Table, Materials and Methods, Figures, Data Availability Section)
Have **primary datasets** been deposited according to the journal's guidelines (see 'Data Deposition' section) and the respective accession numbers provided in the Data Availability Section?	Yes	Data Availability Section
Were **human clinical and genomic datasets** deposited in a public access-controlled repository in accordance to ethical obligations to the patients and to the applicable consent agreement?	Not Applicable	
Are **computational models** that are central and integral to a study available without restrictions in a machine-readable form? Were the relevant accession numbers or links provided?	Not Applicable	
If publicly available data were reused, provide the respective **data citations in the reference list**.	Not Applicable	
